# Direct Transformation from Arylamines to Aryl Naphthalene-1,8-diamino Boronamides: A Metal-Free Sandmeyer-Type Process

**DOI:** 10.3390/molecules24030377

**Published:** 2019-01-22

**Authors:** Siyi Ding, Qiang Ma, Min Zhu, Huaping Ren, Shaopeng Tian, Yuzhen Zhao, Zongcheng Miao

**Affiliations:** Key Laboratory of Organic Polymer Photoelectric Materials, School of Science, Xijing University, Xi’an 710123, Shaanxi, China; dingsiyi2009@163.com (S.D.); coobi@163.com (Q.M.); zhum_588@163.com (M.Z.); hpren2034@163.com (H.R.); tspspirit@163.com (S.T.); zyz19870226@163.com (Y.Z.)

**Keywords:** boronamides, sandmeyer-type reaction, metal-free borylation

## Abstract

A direct metal-free transformation from arylamines to aryl naphthalene-1,8-diamino boronamides, a type of masked boronic acid, has been developed based on Sandmeyer-type reactions. A nonsymmetrical diboron reagent, B(pin)-B(dan), was utilized as the borylating reagent, and the B(dan) moiety was transferred to the aim products selectively. This conversion tolerated a series of functional groups, including chloro, bromo, fluoro, ester, hydroxy, cyano and amide.

## 1. Introduction

Organoboron compounds, because of their increasing utilization in synthetic chemistry, drug discovery and materials science, have attracted significant attention in recent years [1]. They can react with various functional groups to construct new carbon-carbon bonds or carbon-heteroatom bonds, which can rapidly construct the complex structures of target molecules [2,3]. Therefore, much effort has been devoted to the exploration of synthesizing organoboron compounds [4,5,6]. On the other hand, to take best advantage of their diverse reactivity, chemists have also focused on adjusting their reactivity by varying masking groups on the boron atoms [2]. When naphthalene-1,8-diaminato (dan) ligand is used as the masking group, which has two nitrogen atoms that may donate their lone pair electrons to the vacant *p*-orbital of the boron atoms, the formed naphthalene-1,8-diamino boronamide (Bdan) compounds are robust enough to avoid undesirable organic reactions, such as Suzuki-Miyaura coupling reactions. Moreover, such compounds can be easily transformed to their corresponding boronic acids by simple treatment under aqueous acidic conditions. These features enable their wide application as modular synthetical building blocks [4,7,8], especially in the iterative cross-coupling reactions [9,10,11,12] and the application of di-boron compounds [13,14,15,16,17,18].

**Scheme 1:**
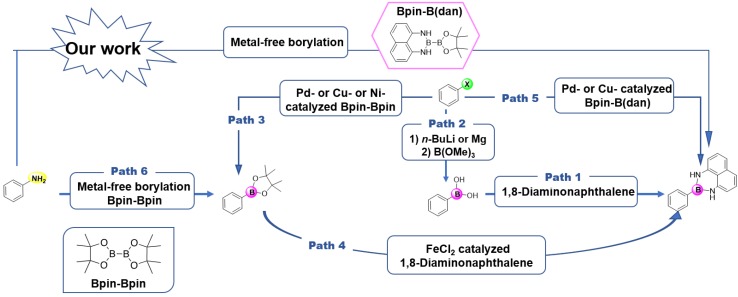
Different routes to synthesize aryl B(dan) derivatives.

When it comes to the synthesis of aryl B(dan) compounds, the most common strategy is condensation between commercially available aryl boronic acids and 1,8-diaminonaphthalene in refluxing toluene (Scheme 1, Path 1) [9]. If aryl halides are used as the starting materials, free boronic acids can be initially obtained via the traditional reactions between aryl magnesium, or –lithium, which derive from metal halogen exchanges, with trimethyl borate followed by hydrolytic workup (Scheme 1, Path 2). Then, aryl B(dan) compounds are accessible via the condensation process (Scheme 1, Path 1). The common products of Miyaura borylation reactions of aryl halides via the catalysis of transition metal such as Pd [19,20], Cu [21] and Ni [22,23] (Scheme 1, Path 3), aryl B(pin), can be converted to their B(dan) derivatives in the presence of FeCl_2_ [24] (Scheme 1, Path 4). In addition to these two indirect approaches, in 2015, Xu and Li reported a direct synthetical pathway of aryl B(dan) from aryl halides [25], which utilized a Pd-catalyzed selective boronyl transfer process of the non-symmetrical diboron reagent B(pin)-B(dan) (Scheme 1, Path 5). Later, similarly processed catalyzed Cu was also proved to be feasible by the Yoshida group [26,27]. As mentioned above, to the best of our knowledge, the synthesis of aryl B(dan) is largely dependent on the accessibility of aryl halides. Moreover, these approaches suffer from sensitivity to air and moisture, in view of the intermediation of aryl metallic compounds [28]. Such a situation inspired our exploration towards metal-free synthesis of aryl B(dan) compounds.

Arylamines, as cheap and abundant organic feedstock, can go through Sandmeyer-type reactions [29,30,31,32], being easily converted into various functional groups. Generally, two steps are required to complete the transformation from arylamines to aryl boronic acids or their derivatives. The amino groups can first be replaced by halides which are then utilized in the borylation reactions, as indicated in Scheme 1. Recently, Wang and co-workers reported a novel metal-free borylation method using arylamines as the starting materials [33,34,35,36] (Scheme 1, Path 6). In this process, symmetrical B(pin)-B(pin) was utilized as the borylating reagent [37,38,39]. In order to obtain aryl B(dan) from arylamines, an additional step is required to complete the conversion (Scheme 1, Path 4). So far, no one has ever realized the conversion from arylamines into their aryl B(dan) derivatives. It was envisaged that the nonsymmetrical B(pin)-B(dan) might be useful to achieve such a process [40,41,42,43,44,45,46]. Inspired by this idea, we sought to apply B(pin)-B(dan) in Sandmeyer-type reactions of arylamines. 

## 2. Results 

At the outset, we carried out the reactions under the reaction conditions that were similar to the previously reported Sandmeyer-type borylation reactions [47,48,49,50,51,52,53,54,55,56,57,58,59,60,61,62], with MeCN as the solvent. We chose 4-methoxyl aniline as the starting material, and no target product could be observed under the classical reaction conditions [*t*-BuONO, MeCN, room temperature]. However, by increasing the reaction temperature to 80 °C, a low yield of 28% was obtained. As the Sandmeyer reaction was well known to involve a radical mechanism, we added the radical initiator benzoyl peroxide (BPO) in subsequent experiments. Consequently, 13% of compound **1a** could be obtained at room temperature, compared with 31% at 80 °C (Table 1, entries 1–4). In view that a base could accelerate the trans-borylation of B_2_pin_2_, we expected that a base could also play the same role for B(pin)-B(dan). Then, our expectations were met. After extensive variation of bases, the yield of reaction was significantly improved by the addition of NaOAc (Table 1, entries 5 and 18–20). Further experiments were conducted to improve the yield and it was noted that the addition of phase-transfer catalyst tetrabutylammonium iodide (TBAI) could enhance the yield of **1a** to 75% (Table 1, entry 9). Lowering the loading of aryl amines was detrimental to this transformation, and 2.0 equivalent of 4-methoxyl aniline worked best in terms of reactivity (Table 1, entries 9, and 14–17). Finally, further assessment of the reaction temperature effect indicated that decreasing or increasing the temperature led to dramatically lower yields (Table 1, entries 9–13).

**Scheme 2:**
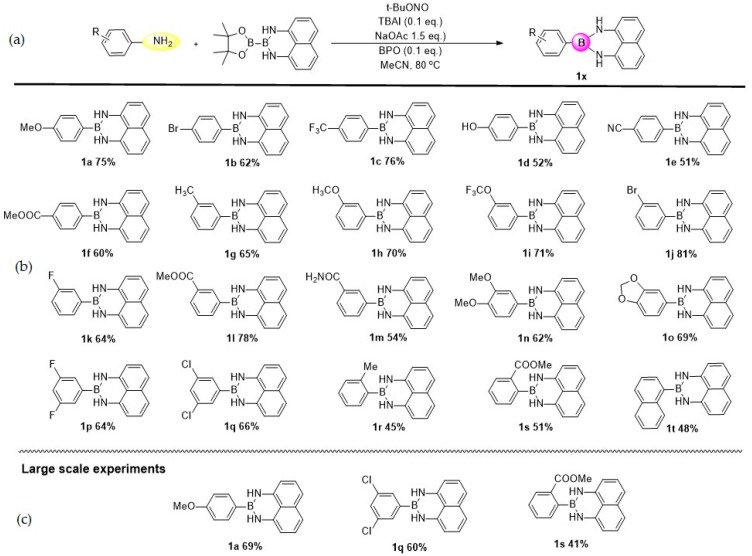
Different routes to synthesize aryl B(dan) derivatives. (**a**) Unless otherwise noted, the reaction conditions are as follows: aryl amine (0.2 mmol, 2.0 eq.), B(pin)-B(dan) (0.1 mmol, 1.0 eq.), *t*-BuONO (0.2 mmol, 2.0 eq.), TBAI (0.01 mmol, 0.1 eq.), NaOAc (0.15 mmol, 1.5 eq.), BPO (0.01 mmol, 0.1 eq.), MeCN (0.6 mL), 80 °C. (**b**) Yield of isolated product. (**c**) Large scale experiments, reaction conditions: aryl amine (2 mmol, 2.0 eq.), B(pin)-B(dan) (1 mmol, 1.0 eq.), *t*-BuONO (2 mmol, 2.0 eq.), TBAI (0.1 mmol, 0.1 eq.), NaOAc (1.5 mmol, 1.5 eq.), BPO (0.1 mmol, 0.1 eq.), MeCN (6.0 mL), 80 °C.

Having defined an appropriate set of reaction conditions, we briefly investigated the scope of arylamines with various functional groups (Scheme 2). For most of the *para*-substituted arylamines (compounds **1a**–**1f**) and *meta*-substituted substrates (compounds **1g**–**1q**), moderate yields were obtained. Then, relatively low yields of compounds **1r** or **1s** were obtained, in which *ortho*-methylphenylamine and *ortho*-(ethoxycarbonyl)aniline were used. These results hinted that this reaction was sensitive to the steric hindrance. Compared with electron-rich arylamines, the anilines with electron-withdrawing groups showed higher reactivity. This new strategy featured broad functional-group tolerance. The anilines bearing functional groups, such as ester (compounds **1f**, **1l** and **1s**), were suitable substrates. Additionally, the moieties with hydroxy (compound **2d**), cyano (compound **1e**) and amide (compound **1m**) were compatible. Halogen atoms of arylamines, such as bromo (compound **1b**, **1j**), chloro (compound **1q**) and fluoro (compound **1c**, **1i**, **1k** and **1p**) atoms, remained intact under the standard conditions, demonstrating the mild nature of the reaction condition. In order to confirm the practicality and potential application of this metal-free borylation process, the reaction was carried out on a 2.0 mmol scale under the standard reaction conditions for selected substrates (*ortho-*, *meta*- and *para*-substituted compounds). The reactions provided the desired derivatives in moderate yields. 

## 3. Discussion 

**Scheme 3:**
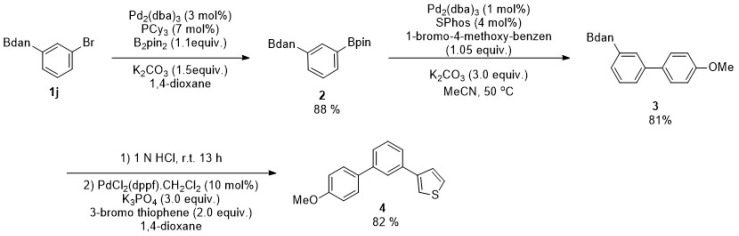
Successive and selective Suzuki-Miyaura cross-coupling reaction using 3-bromophenyl B(dan) **2j** as the starting material.

Based on our previous work on the synthesis and application of differentiated di-boron compounds [18], we applied this new strategy to the preparation of di-boron reagents containing the B(dan) group. Taking the di-functionalized compound **1j** as an example (Scheme 3), the potential application of this product was checked (The details were shown in Supplementary). Firstly, in the presence of Pd(II) catalyst, the Br atom could react with B_2_(pin)_2_ to synthesize the site-differentiated diboron arene **2**. As anticipated [9,14], the reactivities of B(pin) and B(dan), with two effective masking groups for boronic acids on **2**, could be differentiated in the Suzuki-Miyaura coupling reaction. The B(pin) group could be selectively transformed into the aryl while the B(dan) group remained intact. After the workup and purification process, the compound **3** was obtained in 81% yield. In agreement with previous experience, the B(dan) group on compound **3** could be activated by hydrolysis under aqueous condition to obtain its boronic acid, which can be used in the sequential Suzuki-Miyaura cross-coupling reaction to form compound **4**. Therefore, we have demonstrated the usefulness of the naphthalene-1,8-diamino borylation in the facile preparation of boron-differentiated di-boron compounds, which may serve as polyvalent nucleophiles for the modular construction of multifunctionalized poly(hetero)arenes by consecutive cross coupling reactions.

## 4. Materials and Methods

### 4.1. Methods and Material

#### 4.1.1. General Information 

Unless otherwise noted, all reactions were carried out in a flame-dried, sealed Schlenk reaction tube under an atmosphere of nitrogen. Analytical thin-layer chromatography (TLC) was performed on glass plates coated with 0.25 mm 230–400 mesh silica gel containing a fluorescent indicator. Preparative thin-layer chromatography (PTLC) was performed on pre-coated, glass-backed GF254 silica gel plates. Visualization was accomplished by exposure to a UV lamp. All the products in this article are compatible with standard silica gel chromatography. Column chromatography was performed on silica gel (200–300 mesh) using standard methods. 

#### 4.1.2. Structural Analysis 

NMR spectra were measured on a nuclear magnetic resonance apparatus (Avance III HD 400M, Bruker, Germany) and chemical shifts (δ) are reported in parts per million (ppm). ^1^H-NMR spectra were recorded at 400 MHz in NMR solvents and referenced internally to corresponding solvent resonance, and ^13^C-NMR spectra were recorded at 100 MHz and referenced to corresponding solvent resonance. Carbons bearing boron substituents were generally not observed due to quadrupolar relaxation. Coupling constants are reported in Hz with multiplicities denoted as s (singlet), d (doublet), t (triplet), q (quartet), m (multiplet) and br (broad). Infrared spectra were collected on a Thermo Fisher Nicolet 6700 FT-IR spectrometer (Waltham, MA, USA) using ATR (Attenuated Total Reflectance) method. Absorption maxima (ν max) are reported in wavenumbers (cm^−1^). High resolution mass spectra (HRMS) were acquired with an ESI source or APCI source (MTQ III q-TOP, Bruker, Germany).

#### 4.1.3. Materials

Commercial reagents and solvent were purchased from J&K, Energy, Sigma-Aldrich, Alfa Aesar, Acros Organics, Strem Chemicals, TCI and used as received unless otherwise stated. 

### 4.2. General Procedure for the Direct Transformation from Arylamines to Aryl Naphthalene-1,8-diamino Boronamides 

In air, Bpin-B(dan) (0.1 mmol, 1.0 eq.), aryl amide (0.2 mmol, 2.0 eq.), TBAI (0.01 eq.), NaOAc (0.15 eq.), and BPO (0.01 eq.) were sequentially weighed and added to a screw-capped Schenk tube containing a magnetic stir bar. The vessel was evacuated and refilled with nitrogen for three times. *t*-BuONO (0.2 eq.) and MeCN (0.6 mL) were added in turn under N_2_ atmosphere using syringes through a septum which was temporarily used to replace the screw cap. The reaction mixture was then vigorously stirred at 80 °C for the indicated time. The resulting mixture was filtered through a pad of Celite^®^, and the filter cake was washed with ethyl acetate (3 mL × 2). The combined filtrate was evaporated under vacuum to dryness and the residue was purified by column chromatography to yield the desired product.

### 4.3. Analytical Data of Products ***1a**–**1t***

2-(4-methoxyphenyl)-2,3-dihydro-1H-naphtho[1,8-de][1,3,2]diazaborinine (**1a**, CAS: 1159803-53-8) [25]. Yield: 20.6 mg (75%); white solid; m.p.: 163.2~165.6 °C; IR (cm^−1^): 3407, 1594, 1495, 1407, 1224, 1181, 1029; ^1^H-NMR (400 MHz, CDCl_3_) δ 7.59 (d, *J* = 8.8 Hz, 2H), 7.15 (t, *J* = 8.0 Hz, 2H), 7.05 (d, *J* = 8.0 Hz, 2H), 6.98 (d, *J* = 8.4 Hz, 2H), 6.42 (d, *J* = 7.2 Hz, 2H), 6.00 (s, 2H), 3.86 (s, 3H); ^13^C-NMR (100 MHz, CDCl_3_) δ 161.4, 141.2, 140.3, 136.4, 133.0, 127.6, 117.7, 113.9, 105.9, 55.2; ^11^B-NMR (128 MHz, CDCl_3_) δ 29.1; HRMS (APCI) *m*/*z* calcd for C_17_H_14_BN_2_O (M^−^): 273.1205, found: 273.1203. 

2-(4-bromophenyl)-2,3-dihydro-1H-naphtho[1,8-de][1,3,2]diazaborinine (**1b**) [9]. Yield: 20.0 mg (62%); white solid; m.p.: 135.6~136.2 °C; IR (cm^−1^): 3408.9, 2921.3, 2851.4, 2362.0, 2342.0, 1596.0, 1511.4, 1400.3, 1373.3, 817.5, 752.2, 690.0; ^1^H-NMR (400 MHz, CDCl_3_) δ 7.57 (d, *J *= 8.2 Hz, 2H), 7.48 (d, *J *= 8.2 Hz, 2H), 7.14 (t,* J *= 7.8 Hz, 2H), 7.06 (d, *J* = 8.0 Hz, 2H), 6.40 (d, *J *= 7.2 Hz, 2H), 5.96 (s, 2H); ^13^C-NMR (100 MHz, CDCl_3_) δ 140.8, 136.3, 133.0, 131.5, 127.7, 124.9, 119.9, 118.1, 106.2; HRMS (APCI) *m*/*z* calcd for C_16_H_12_BBrN_2_ (M^−^): 322.0282, found: 322.0279. 

2-(4-(trifluoromethyl)phenyl)-2,3-dihydro-1H-naphtho[1,8-de][1,3,2]diazaborinine (**1c**) [25]. Yield: 23.7 mg (76%); white solid; m.p.: 127.0~130.3 °C; IR (cm^−1^): 3414.2, 2365.0, 2341.9, 1527.7, 1493.5, 1398.9, 1166.0, 1035.0, 826.8, 747.1; ^1^H-NMR (400 MHz, CDCl_3_) δ 7.75 (d, *J *= 7.9 Hz, 2H), 7.69 (d, *J *= 8.0 Hz, 2H), 7.13 (t,* J *=7.8 Hz, 2H), 7.09 (d, *J* = 7.8 Hz, 2H), 6.44 (dd, *J* = 7.2, 0.8 Hz, 2H), 6.01 (s, 2H); ^13^C-NMR (100 MHz, CDCl_3_) δ 140.8, 136.5, 132.2 (q, *J* = 32 Hz), 131.9, 127.8, 125.1 (q,* J *= 4 Hz), 124.2 (q, *J* = 272 Hz), 120.1, 118.4, 103.8; ^11^B-NMR (128 MHz, CDCl_3_) δ 30.1; ^19^F-NMR (377 MHz, CDCl_3_) δ −62.88; HRMS (APCI) *m*/*z* calcd for C_17_H_11_BF_3_N_2_ (M^−^): 311.0973, found: 311.0974.

4-(1H-naphtho[1,8-de][1,3,2]diazaborinin-2(3H)-yl)phenol (**1d**) [25]. Yield: 13.5 mg (52%); white solid; m.p.: 222.6~225.7 °C; IR (cm^−1^): 3441.0, 3414.9, 3028.7, 2361.5, 2343.2, 1582.3, 1487.1, 1404.1, 1200.2, 1177.8, 813.5, 753.2; ^1^H-NMR (400 MHz, CDCl_3_) δ 7.55 (d,* J* = 8.2 Hz, 2H), 7.14 (t, *J* = 7.8 Hz, 2H), 7.05 (d, *J* = 8.2 Hz, 2H), 6.91 (d,* J *= 8.3 Hz, 2H), 6.41 (d,* J *= 7.3 Hz, 2H), 5.98 (s, 2H), 4.90 (s, 1H); ^13^C-NMR (100 MHz, CDCl_3_) δ 157.4, 141.2, 136.3, 133.3, 127.6, 119.7, 117.7, 115.3, 105.9; HRMS (APCI) *m*/*z* calcd for C_16_H_12_BN_2_O (M^−^): 259.1048, found: 259.1049.

4-(1H-naphtho[1,8-de][1,3,2]diazaborinin-2(3H)-yl)benzonitrile (**1e**) [25]. Yield: 13.7 mg (51%); white solid; m.p.: 220.4~225.1 °C; IR (cm^−1^): 3409.3, 3401.7, 3393.0, 3370.2, 2362.0, 2220.1, 1516.3, 1593.4, 1405.2, 1082.6, 818.4; ^1^H-NMR (400 MHz, CDCl_3_) δ 7.72 (dd,* J *= 8.0, 3.2 Hz, 4H), 7.14 (t, *J *= 7.7 Hz, 2H), 7.09 (d, *J *= 8.0 Hz, 2H), 6.43 (d, *J *= 7.2 Hz, 2H), 5.99 (s, 2H); ^13^C-NMR (100 MHz, CDCl_3_) δ 140.4, 136.3, 132.0, 131.7, 127.7, 120.0, 118.7, 118.5, 113.7, 106.4; ^11^B-NMR (128 MHz, CDCl_3_) δ 28.8; HRMS (ESI) *m*/*z* calcd for C_17_H_12_BN_3_Na (M^+^): 292.1022, found: 292.1014.

methyl 4-(1H-naphtho[1,8-de][1,3,2]diazaborinin-2(3H)-yl)benzoate (**1f**). Yield: 24.5 mg (81%); white solid; m.p.: 202.2~203.6 °C; IR (cm^−1^): 3387.9, 2920.4, 2849.8, 2364.4, 2342.4, 1705.0, 1592.7, 1397.3, 759.4, 704.7; ^1^H-NMR (400 MHz, CDCl_3_) δ 8.09 (d,* J* = 8.0 Hz, 2H), 7.71 (d, *J* = 7.9 Hz, 2H), 7.15 (t, *J* = 7.8 Hz, 2H), 7.07 (d, *J *= 8.2 Hz, 2H), 6.43 (d, *J* = 7.2 Hz, 2H), 6.05 (s, 2H). 3.95 (s, 3H); ^13^C-NMR (100 MHz, CDCl_3_) δ 167.0, 140.8, 136.4, 131.6, 131.5, 129.2, 127.6, 120.0, 118.2, 106.2, 52.3; HRMS (APCI) *m*/*z* calcd for C_18_H_15_BN_2_O_2_ (M^−^): 302.1232, found: 302.1230. 

2-(m-tolyl)-2,3-dihydro-1H-naphtho[1,8-de][1,3,2]diazaborinine (**1g**) [25]. Yield: 18.3 mg (71%); white solid; m.p.: 103.8~106.3 °C; IR (cm^−1^): 3409.1, 3050.8, 3029.9, 1593.0, 1581.1, 1326.2, 817.9, 762.0, 702.8; ^1^H-NMR (400 MHz, CDCl_3_) δ 7.44 (d, *J *= 8.2 Hz, 2H), 7.34 (m, 2H), 7.14 (t,* J* = 7.8 Hz, 2H), 7.05 (d, *J* = 8.2 Hz, 2H), 6.41 (d, *J *= 7.3 Hz, 2H), 6.02 (s, 2H), 2.42 (s, 3H); ^13^C-NMR (100 MHz, CDCl_3_) δ 141.2, 137.7, 136.4, 132.2, 131.1, 128.5, 128.2, 127.6, 119.9, 117.8, 106.0, 21.6; ^11^B NMR (128 MHz, CDCl_3_) δ 30.4; HRMS (APCI) *m*/*z* calcd for C_17_H_14_BN_2_ (M^−^): 257.1256, found: 257.1257.

2-(3-methoxyphenyl)-2,3-dihydro-1H-naphtho[1,8-de][1,3,2]diazaborinine (**1h**) [25]. Yield: 19.2 mg (70%); white solid; m.p.: 113.7~116.4 °C; IR (cm^−1^): 3452.9, 3409.8, 3049.7, 3032.7, 1593.0, 1515.4, 1478.6, 1406.1, 1243.3, 693.5, 658.1; ^1^H-NMR (400 MHz, CDCl_3_) δ 7.37 (t,* J *= 7.7 Hz, 1H), 7.21 (d, *J *= 7.2 Hz, 1H), 7.14 (m, 3H), 7.05 (d, *J *= 8.1 Hz, 2H), 7.00 (dd, *J *= 8.1, 2.0 Hz, 1H), 6.40 (d, *J *= 7.3 Hz, 2H), 6.00 (s, 2H), 3.86 (s, 3H); ^13^C-NMR (100 MHz, CDCl_3_) δ 159.5, 141.1, 136.4, 129.6, 127.7, 123.9, 119.9, 117.9, 117.0, 115.5, 106.1, 55.3; ^11^B-NMR (128 MHz, CDCl_3_) δ 30.1; HRMS (APCI) *m*/*z* calcd for C_17_H_14_BN_2_O (M^−^): 273.1205, found: 273.1207. 

2-(3-(trifluoromethoxy)phenyl)-2,3-dihydro-1H-naphtho[1,8-de][1,3,2]diazaborinine (**1i**). Yield: 21.3 mg (65%); brown oil; IR (cm^−1^): 3414.2, 2365.0, 2341.9, 1527.7, 1493.5, 1398.9, 1166.0, 1035.0, 826.8, 747.1; ^1^H-NMR (400 MHz, CDCl_3_) δ 7.56 (d, *J *= 7.4 Hz, 2H), 7.48 (m, 2H), 7.33 (d, *J* = 8.1 Hz, 1H), 7.16 (t, *J* = 7.7 Hz, 2H), 7.08 (d,* J* = 8.2 Hz, 2H), 6.43 (d, *J* = 7.2 Hz, 2H), 5.98 (s, 2H); ^13^C-NMR (100 MHz, CDCl_3_) δ 149.4, 140.7, 136.3, 129.8, 127.7, 123.7, 122.7, 119.9, 118.2, 106.1; HRMS (APCI) *m*/*z* calcd for C_17_H_12_BF_3_N_2_O (M^−^): 328.1000, found: 328.1002. 

2-(3-bromophenyl)-2,3-dihydro-1H-naphtho[1,8-de][1,3,2]diazaborinine (**1j**) [9]. Yield: 19.3 mg (60%); white solid; m.p.: 86.6~87.9 °C; IR (cm^−1^): 3408.9, 2921.3, 2851.4, 2362.0, 2342.0, 1596.0, 1511.4, 1400.3, 1373.3, 817.5, 752.2, 690.0; ^1^H-NMR (400 MHz, CDCl_3_) δ 7.73 (s, 1H), 7.57 (d,* J* = 8.0 Hz, 1H), 7.51 (d, *J *= 7.4 Hz, 1H), 7.29 (t, *J* = 7.7 Hz, 1H), 7.13 (t, *J *= 7.8 Hz, 2H), 7.05 (d, *J *= 7.9 Hz, 2H), 6.39 (d, *J *= 7.2 Hz, 2H), 5.94 (s, 2H); ^13^C-NMR (100 MHz, CDCl_3_) δ 140.7, 136.3, 134.4, 133.2, 130.1, 129.9, 127.7, 123.1, 119.9, 118.1, 106.3; HRMS (APCI) *m*/*z* calcd for C_16_H_12_BBrN_2_ (M^−^): 322.0282, found: 322.0280.

2-(3-fluorophenyl)-2,3-dihydro-1H-naphtho[1,8-de][1,3,2]diazaborinine (**1k**). Yield: 16.8 mg (64%); yellow solid; m.p.: 103.3~104.6 °C; IR (cm^−1^): 3442.7, 3435.4, 3032.2, 1595.8, 1520.5, 1371.4, 757.1, 749.4, 686.3; ^1^H-NMR (400 MHz, CDCl_3_) δ 7.41 (m, 2H), 7.33 (dd, *J *= 2.4, 9.2 Hz, 1H), 7.14 (m, 3H), 7.06 (d, *J* = 8.0 Hz, 2H), 6.41 (d,* J *= 7.3 Hz, 2H), 5.97 (s, 2H); ^13^C-NMR (100 MHz, CDCl_3_) δ 162.9 (d,* J* = 246 Hz), 140.8, 136.3, 130.1 (d, *J* = 7 Hz), 127.7, 127.0 (d,* J *= 3 Hz), 119.9, 118.1, 118.0 (d, *J* = 23 Hz), 117.1 (d, *J* = 20 Hz), 106.2; HRMS (APCI) *m*/*z* calcd for C_16_H_12_BFN_2_ (M^−^): 262.1083, found: 262.1080. 

Methyl 3-(1H-naphtho[1,8-de][1,3,2]diazaborinin-2(3H)-yl)benzoate (**1l**). Yield: 23.6 mg (78%); pink solid; m.p.: 178.9~179.8 °C; IR (cm^−1^): 3452.9, 3409.8, 2049.7, 1515.4, 1478.6, 1077.0, 816.1; ^1^H-NMR (400 MHz, CDCl_3_) δ 8.33 (s, 1H), 8.13 (dt,* J *= 8.8, 1.4 Hz, 1H), 7.83 (d, *J *= 7.4 Hz, 1H), 7.52 (t, *J *= 7.6 Hz, 1H), 7.15 (t,* J *= 7.8 Hz, 2H), 7.07 (d, *J *= 8.0 Hz, 2H), 6.45 (d,* J* = 7.2 Hz, 2H), 6.09 (s, 2H), 3.97 (s, 3H); ^13^C-NMR (100 MHz, CDCl_3_) δ 167.2, 140.8, 136.3, 135.9, 132.6, 131.3, 130.0, 128.4, 127.7, 119.9, 118.1, 106.2, 52.3; HRMS (APCI) *m*/*z* calcd for C_18_H_15_BN_2_O_2_ (M^−^): 302.1232, found: 302.1228. 

3-(1H-naphtho[1,8-de][1,3,2]diazaborinin-2(3H)-yl)benzamide (**1m**). Yield: 15.5 mg (54%); brown solid; m.p.: 168.4~169.5 °C; IR (cm^−1^): 3382.8, 3101.1, 3048.7, 2341.2, 2366.2, 1599.3, 1568.7, 1506.6, 816.3, 758.5, 668.6; ^1^H-NMR (400 MHz, CDCl_3_) δ 7.85 (s, 1H), 7.49 (m, 1H), 7.36 (m, 3H), 7.30 (s, 1H), 7.13 (t, *J* = 7.7 Hz, 2H), 7.05 (d, *J* = 8.2 Hz, 2H), 6.39 (d, *J* = 7.0 Hz, 2H), 6.05 (s, 2H); ^13^C-NMR (100 MHz, CDCl_3_) δ 168.6, 141.0, 137.8, 136.3, 129.0, 127.6, 127.5, 122.9, 121.7, 119.9, 117.8, 106.1; HRMS (APCI) *m*/*z* calcd for C_17_H_14_BN_3_O (M^−^): 287.1235, found: 287.1233. 

2-(3,4-dimethoxyphenyl)-2,3-dihydro-1H-naphtho[1,8-de][1,3,2]diazaborinine (**1n**). Yield: 18.8 mg (62%); light pink solid; m.p.: 174.5~175.0 °C; IR (cm^−1^): 3407.2, 1594.4, 1495.9, 1407.7, 1224.1, 1181.2, 1029.0; ^1^H-NMR (400 MHz, CDCl_3_) δ 7.24 (d,* J* = 7.9 Hz, 1H), 7.13 (m, 3H), 7.06 (d, *J* = 8.2 Hz, 2H), 6.96 (d, *J *= 7.9 Hz, 1H), 6.43 (d, *J *= 7.2 Hz, 2H), 5.99 (s, 2H), 3.97 (s, 3H), 3.93 (s, 3H); ^13^C-NMR (100 MHz, CDCl3) δ 150.9, 148.9, 141.1, 136.4, 127.6, 124.9, 119.7, 117.8, 113.8, 111.1, 106.0, 56.1, 55.8; HRMS (APCI) *m*/*z* calcd for C_18_H_17_BN_2_O_2_ (M^−^): 304.1389, found: 304.1385. 

2-(benzo[d][1,3]dioxol-5-yl)-2,3-dihydro-1H-naphtho[1,8-de][1,3,2]diazaborinine (**1o**) [25]. Yield: 19.9 mg (69%); white solid; m.p.: 173.5~175.8 °C; IR (cm^−1^): 3399.2, 1594.3, 1478.2, 1402.7, 1232.1, 1034.0; ^1^H-NMR (400 MHz, CDCl_3_) δ 7.15 (t, *J* = 7.4 Hz, 3H), 7.06 (t, *J *= 6.6 Hz, 3H), 6.91 (d, *J *= 7.6 Hz, 1H), 6.40 (d,* J *= 7.2 Hz, 2H), 6.00 (s, 2H), 5.94 (s, 2H); ^13^C-NMR (100 MHz, CDCl_3_) δ 149.4, 147.8, 141.1, 136.3, 127.6, 125.8, 119.7, 117.8, 110.9, 108.8, 106.0, 101.0; HRMS (ESI) *m*/*z* calcd for C_17_H_14_BN_2_O_2_ (M^+^): 289.1148, found: 289.1147.

2-(3,5-difluorophenyl)-2,3-dihydro-1H-naphtho[1,8-de][1,3,2]diazaborinine (**1p**). Yield: 17.9 mg (64%); yellow solid; m.p.: 113.2~114.6 °C; IR (cm^−1^): 3414.2, 2365.0, 2341.9, 1398.8, 1317.8, 1105.6, 1082.3, 818.4; ^1^H-NMR (400 MHz, CDCl_3_) δ 7.75 (d,* J *= 7.9 Hz, 2H), 7.68 (d, *J *= 8.0 Hz, 2H), 7.16 (t, *J* = 7.8 Hz, 2H), 7.08 (d,* J *= 7.8 Hz, 2H), 6.43 (dd, *J* = 7.2, 0.6 Hz, 2H), 6.01 (s, 2H); ^13^C-NMR (100 MHz, CDCl_3_) δ 160.7 (dd, *J *= 249 Hz, 6 Hz), 137.9, 133.8, 125.1, 117.4, 115.8, 111.3 (dd, *J *= 17 Hz, 6 Hz), 103.8, 102.9 (t,* J *= 25 Hz); HRMS (APCI) *m*/*z* calcd for C_16_H_11_BN_2_F_2_ (M^−^): 280.0989, found: 280.0991. 

2-(3,5-dichlorophenyl)-2,3-dihydro-1H-naphtho[1,8-de][1,3,2]diazaborinine (**1q**) [63]. Yield: 20.6 mg (66%); green solid; m.p.: 164.2~165.8 °C; IR (cm^−1^): 3381.9, 2946.7, 2364.1, 1559.7, 1507.0, 1266.1, 1068.1, 746.4; ^1^H-NMR (400 MHz, CDCl_3_) δ 7.46 (dd,* J *= 8.3, 1.7 Hz, 3H), 7.15 (t, *J *= 8.0 Hz, 2H), 7.08 (d,* J* = 8.0 Hz, 2H), 6.42 (d*, J *= 7.2 Hz, 2H), 5.94 (s, 2H); ^13^C-NMR (100 MHz, CDCl_3_) δ 140.4, 136.3, 135.3, 130.1, 129.7, 127.7, 120.0, 118.4, 106.4; HRMS (APCI) *m*/*z* calcd for C_16_H_11_BN_2_Cl_2_ (M^−^): 312.0398, found: 312.0395. 

2-(*o*-tolyl)-2,3-dihydro-1H-naphtho[1,8-de][1,3,2]diazaborinine (**1r**) [25]. Yield: 11.6 mg (45%); white solid; m.p.: 73.2~75.1 °C; IR (cm^−1^): 3420.1, 3404.9, 2360.8, 2341.0, 1594.1, 1506.1, 1325.8, 1318.1, 1077.9, 818.4, 656.0; ^1^H-NMR (400 MHz, CDCl_3_) δ 7.44 (d, *J* = 7.3 Hz, 1H), 7.31 (t, *J *= 7.5 Hz, 1H), 7.21 (m, 2H), 7.12 (t,* J *= 7.8 Hz, 2H), 7.05 (d, *J *= 8.5 Hz, 2H), 6.33 (d, *J *= 7.2 Hz, 2H), 5.80 (s, 2H), 2.49 (s, 3H); ^13^C-NMR (100 MHz, CDCl_3_) δ 141.1, 140.7, 136.4, 132.3, 129.7, 129.3, 127.7, 125.3, 119.8, 117.9, 105.9, 22.4; ^11^B NMR (128 MHz, CDCl_3_) δ 30.0; HRMS (APCI) *m*/*z* calcd for C_17_H_14_BN_2_ (M^−^): 257.1256, found: 257.1257. 

Methyl 2-(1H-naphtho[1,8-de][1,3,2]diazaborinin-2(3H)-yl)benzoate (**1s**) [25]. Yield: 15.4 mg (51%); white solid; m.p.: 160.3~164.5 °C; IR (cm^−1^): 3381.9, 2946.7, 2364.1, 1699.4, 1507.6, 1134.7, 1266.1, 1068.1, 816.3, 746.4; ^1^H-NMR (400 MHz, CDCl_3_) δ 8.04 (d,* J *= 7.8 Hz, 1H), 7.54 (m, 2H), 7.47 (m, 1H), 7.11 (t, *J *= 7.8 Hz, 2H), 7.04 (d, *J *= 7.8 Hz, 2H), 6.31 (d, *J *= 7.2 Hz, 2H), 5.73 (s, 2H), 3.86 (s, 3H); ^13^C-NMR (100 MHz, CDCl_3_) δ 168.3, 141.3, 136.4, 133.2, 132.7, 132.1, 129.6, 128.9, 127.6, 119.6, 117.6, 105.8, 52.4; ^11^B NMR (128 MHz, CDCl_3_) δ 31.0; HRMS (APCI) *m*/*z* calcd for C_18_H_14_BN_2_O_2_ (M^−^): 301.1154, found: 301.1157. 

2-(naphthalen-1-yl)-2,3-dihydro-1H-naphtho[1,8-de][1,3,2]diazaborinine (**2t**) [25]. Yield: 14.1 mg (48%); white solid; m.p.: 140.2~143.6 °C; IR (cm^−1^): 3420.2, 3402.4, 1594.6, 1508.8, 1498.6, 1315.5, 1167.3; ^1^H-NMR (400 MHz, CDCl_3_) δ 8.22 (dd, *J* = 6.4, 3.2 Hz, 1H), 7.91 (m, 2H), 7.70 (d, *J *= 6.4 Hz, 1H), 7.52 (m, 3H), 7.17 (t, *J* = 8.0 Hz, 2H), 7.11 (d, *J* = 8.0 Hz, 2H), 6.38 (d. *J* = 7.2 Hz, 2H), 6.02 (s, 2H); ^13^C-NMR (100 MHz, CDCl_3_) δ 141.1, 136.4, 135.4, 133.3, 130.7, 129.6, 128.8, 127.9, 127.7, 126.3, 125.9, 125.4, 120.0, 118.0, 106.1; ^11^B-NMR (128 MHz, CDCl_3_) δ 30.7; HRMS (ESI) *m*/*z* calcd for C_20_H_16_BN_2_ (M^+^): 295.1407, found: 295.1399.

## 5. Conclusions

In conclusion, by employing the non-symmetrical di-boron compound, B(pin)-B(dan), as the borylating reagent, we have realized a metal-free Sandmeyer-type borylation reaction under relatively mild conditions and afforded various aryl B(dan) compounds in moderate yields. The procedure tolerates a series of functional groups, including chloro, bromo, fluoro, ester, hydroxy, cyano and amide groups. Further studies on the mechanism are in progress in our laboratory (The proposed mechanism was shown in the Supporting Information). We anticipate that this protocol described herein could serve as an important supplement to the existing strategies for preparing the aryl-B(dan) compounds and will then find wide application in organic synthesis and related fields.

## Figures and Tables

**Table 1:**
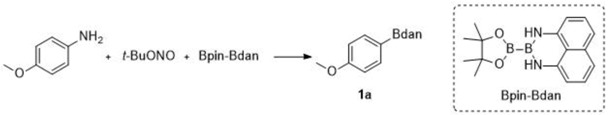
Optimization of reaction conditions.

Entry ^1^	ArNH_2_	Base	Additive	T (°C)	Yield ^2^ (%)
1	2.0 eq.	-	-	RT	-
2	2.0 eq.	-	-	80	28
3	2.0 eq.	-	BPO (benzoyl peroxide) (0.1 eq.)	RT	13
4	2.0 eq.	-	BPO (0.1eq.)	80	31
5	2.0 eq.	NaOAc (1.5 eq.)	BPO (0.1 eq.)	80	68
6	2.0 eq.	NaOAc (1.5 eq.)	-	80	-
7	2.0 eq.	-	BPO (0.1 eq.), TBAI (tetrabutylammonium iodide) (0.1 eq.)	80	64
8	2.0 eq.	-	TBAI (0.1 eq.)	80	22
**9** ^**3**^	**2.0 eq.**	**NaOAc (1.5 eq.)**	**BPO (0.1 eq.), TBAI (0.1 eq.)**	**80**	**75**
10	2.0 eq.	NaOAc (1.5 eq.)	BPO (0.1 eq.), TBAI (0.1 eq.)	RT	10
11	2.0 eq.	NaOAc (1.5 eq.)	BPO (0.1 eq.), TBAI (0.1 eq.)	50	35
12	2.0 eq.	NaOAc (1.5 eq.)	BPO (0.1 eq.), TBAI (0.1 eq.)	100	50
13	2.0 eq.	NaOAc (1.5 eq.)	BPO (0.1 eq.), TBAI (0.1 eq.)	120	20
14	1.0 eq.	NaOAc (1.5 eq.)	BPO (0.1 eq.), TBAI (0.1 eq.)	80	33
15	1.2 eq.	NaOAc (1.5 eq.)	BPO (0.1 eq.), TBAI (0.1 eq.)	80	41
16	1.5 eq.	NaOAc (1.5 eq.)	BPO (0.1 eq.), TBAI (0.1 eq.)	80	52
17	1.8 eq.	NaOAc (1.5 eq.)	BPO (0.1 eq.), TBAI (0.1 eq.)	80	58
18	2.0 eq.	*t*-BuOK (1.5 eq.)	BPO (0.1 eq.), TBAI (0.1 eq.)	80	29
19	2.0 eq.	CsCO_3_ (1.5 eq.)	BPO (0.1 eq.), TBAI (0.1 eq.)	80	trace
20	2.0 eq.	NaOCH_3_ (1.5 eq.)	BPO (0.1 eq.), TBAI (0.1 eq.)	80	32

^1^ Reaction conditions: aniline (0.2 mmol), *t*-BuONO (0.2 mmol), Bpin-B(dan) (0.1 mmol), MeCN (0.6 mL). The reaction was stirred in N_2_ atmosphere for 6 h. ^2^ Isolated yield. ^3^ Entries in bold represent optimized reaction conditions.

## Supplementary Materials

Experimental procedures and spectral data for the borylated products. This material is available free of charge via the Internet.
