# Supplementary material for: Direct Transformation from Arylamines to Aryl Naphthalene-1,8-diamino Boronamides: A Metal-Free Sandmeyer-Type Process
Source: Molecules. 2019 Jan 22;24(3):377. doi: 10.3390/molecules24030377 (PMC6384809; doi:10.3390/molecules24030377)
Supplement: Supplementary file 1 [file molecules-24-00377-s001.pdf]

Supporting Information

Direct Transformation from Arylamines to Aryl Naphthalene-1,8-diamino Boronamides: A Metal-Free Sandmeyer-type Process

Siyi Ding[a], Qiang Ma[a], Min Zhu[a], Huaping Ren[a], Shaopeng Tian[a], Yuzhen Zhao[a], Zongcheng Miao[a,†]

a. Department of Applied Science, Xijing University, Xi'an, Shaanxi 710000, China.

Email: miaozongcheng@xijing.edu.cn

1. Direct Transformation from Arylamines to Aryl Naphthalene-1,8-diamino Boronamides: A Metal-Free Sandmeyer-type Process 2

1.1 Proposed mechanism for this process 2

1.2 General Procedure for the synthesis of nonsymmetrical diboron reagent Bpin-Bdan[1,2] 2

1.3 General Procedure for the application of 3-bromophenyl B(dan) 1j in the selective Suzuki-Miyaura cross-coupling reaction 3

2. Copy of NMR spectra 6

1. Direct Transformation from Arylamines to Aryl Naphthalene-1,8-diamino Boronamides: A Metal-Free Sandmeyer-type Process

1.1 Proposed mechanism for this process

A radical mechanism has been proposed to account for this borylation reaction [47-61] as shown in the following Scheme. Firstly, the amino group is transformed to generate the diazonium salt **A** in the presence of t-BuONO through the Sandmeyer reaction, which was well documented as a radical mechanism [29-32]. On the other hand, the acetate anion reacts with Bpin-Bdan to form the tetra-coordinated boron complex **C**. Then a single-electron transformation (SET) process occurred between aryldiazonium ion **B** and complex **C** to generate aryl radical F through N_2 extrusion from radical **D** together with the formation of complex **E**. Finally, the reaction between aryl radical **F** with complex **E** gave the borylated product aryl-Bdan.

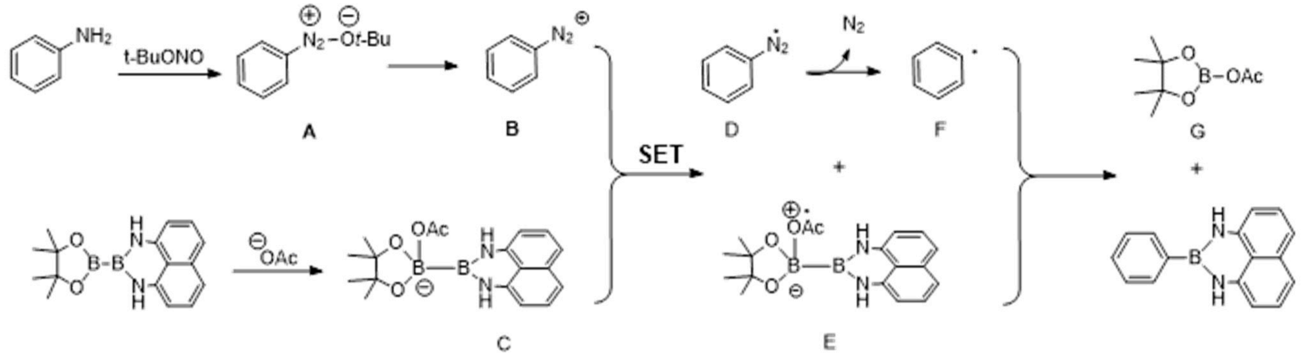

Scheme 1. Possible reaction mechanism.

1.2 General Procedure for the synthesis of nonsymmetrical diboron reagent Bpin-Bdan[1,2]

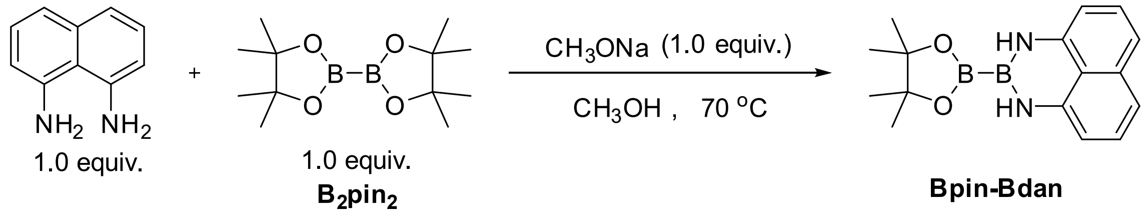

In air, B_2pin_2 (254.2 mg, 1.0 mmol, 1.0 eq.), 1,8-diaminonaphthalene (158.1 mg, 1.0 mmol, 1.0 eq.), and CH_3ONa (54.0 mg, 1.0 mmol, 1.0 eq.) were sequentially weighed and

added to a screw-capped Schenk tube containing a magnetic stir bar. The vessel was evacuated and refilled with nitrogen for three times. MeOH (10.0 mL) were added in turn under N_2 atmosphere using syringes through a septum which was temporarily used to replace the screw cap. The reaction mixture was then vigorously stirred at 70 ^oC for 4 hours. The resulting mixture was evaporated under vacuum to dryness and the residue was purified by column chromatography to yield the desired product Bpin-Bdan 235.4 mg, 80 %.

Melting point: 186.7–187.4 °C;

IR (cm^{-1}) 3382, 3053, 2975, 1627, 1603, 1372, 1316, 1291, 1267, 1250, 1212, 1165, 1144, 1106, 962, 862, 849, 821, 765, 635;

^1H NMR (400 MHz, $CDCl_3$) δ 1.29 (s, 12H), 6.19 (br s, 2H), 6.26 (dd, J = 7.2, 0.8 Hz, 2H), 6.97 (dd, J = 8.4, 0.8 Hz, 2H), 7.07 (dd, J = 8.4, 7.2, 2H);

^{13}C NMR (100 MHz, $CDCl_3$) δ 25.6, 83.8, 105.9, 118.1, 121.6, 128.0, 136.9, 141.0;

^{11}B NMR (128 MHz, $CDCl_3$) δ 32.7;

1.3 General Procedure for the application of 3-bromophenyl B(dan) 1j in the selective Suzuki-Miyaura cross-coupling reaction

2-(3-(4,4,5,5-tetramethyl-1,3,2-dioxaborolan-2-yl)phenyl)-2,3-dihydro-1*H*-naphtho[1,8-*de*][1,3,2]diazaborinine (2) [3]

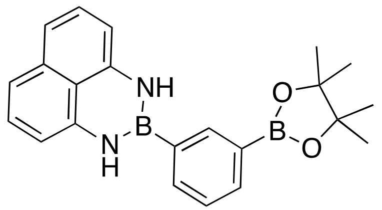

An oven dried test tube was charged with **2j** (151 mg, 0.47 mmol), B_2pin_2 (130 mg, 0.51 mmol), $Pd_2(dba)_3$ (8.0 mg, 0.014 mmol), PCy_3 (9.4 mg, 0.033 mmol) and K_2CO_3 (68.3 mg, 0.7 mmol). The reaction tube was evacuated and backfilled with argon, and 1,4-dioxane (1.3 mL) was added via a syringe. The reaction mixture was stirred for 5h at 80 ^oC. The reaction mixture was diluted with EtOAc, and filtered through a pad of celite, and the filtrate was concentrated under reduced pressure. The crude product was purified by column chromatography by PE/EA (10:1) to get the product as light grey solid (128.3 mg, 88 %).

Melting point: 106.1~107.2 ^oC;

IR (cm^{-1}): 3420.2, 3402.4, 1594.6, 1508.8, 1498.6, 1315.5, 1167.3;

^1H NMR (400 MHz, $CDCl_3$) δ 7.76 (s, 1H), 7.59 (d, *J* = 8.0 Hz, 1H), 7.55 (d, *J* = 7.4 Hz, 1H), 7.31 (t, *J* = 7.7 Hz, 1H), 7.14 (t, *J* = 7.8 Hz, 2H), 7.07 (d. *J* = 8.2 Hz, 2H), 6.42 (d, *J* = 7.2 Hz, 2H), 5.98 (s, 2H), 1.26 (s, 12H);

^{13}C NMR (100 MHz, $CDCl_3$) δ 140.7, 136.3, 134.4, 133.2, 131.4, 130.0, 129.9, 127.6, 123.1, 119.9, 118.2, 106.2, 83.5, 25.0;

HRMS (APCI) m/z calcd for $C_{22}H_{24}B_2N_2O_2$ (M^-): 370.2029, found: 370.2026.

2-(4'-methoxy-[1,1'-biphenyl]-3-yl)-2,3-dihydro-1*H*-naphtho[1,8-*de*][1,3,2]diazaborinine (3)[3]

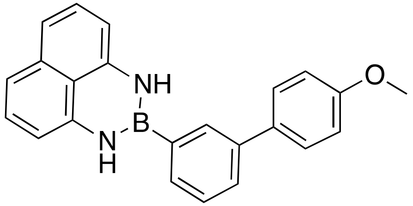

An oven dried test tube was charged with **2j** (80.5 mg, 0.25 mmol), p-methoxylphenylboronic acid (77 mg, 0.50 mmol), $PdCl_2$ (dppf)·CH_2Cl_2 (21 mg, 0.025 mmol) and K_3PO_4 (160 mg, 0.76 mmol). The reaction tube was evacuated and backfilled with argon, and 1,4-dioxane (1.3 mL) was added via a syringe. The reaction mixture was stirred for 5h at 80 ^{o}C. The reaction mixture was diluted with EtOAc, and filtered through a pad of celite, and the filtrate was concentrated under reduced pressure. The crude product was purified by column chromatography by PE/EA (10:1) to get the product (78.8 mg, 90 %).

Melting point: 163.2~165.6 ^{o}C;

IR (cm^{-1}): 3407.2, 1594.4, 1495.9, 1407.7, 1224.1, 1181.2, 1029.0;

^{1}H NMR (400 MHz, $CDCl_3$) δ 7.80 (s, 1H), 7.65 (d, *J* = 7.1 Hz, 1H), 7.57 (m, 3H), 7.50 (t, *J* = 7.4 Hz, 1H), 7.15 (t, *J* = 7.7 Hz, 2H), 7.07 (d. *J* = 8.2 Hz, 2H), 7.02 (d, *J* = 8.4 Hz, 2H), 6.44 (d, *J* = 7.2 Hz, 2H), 6.08 (s, 2H), 3.88 (s, 3H);

^{13}C NMR (100 MHz, $CDCl_3$) δ 159.3, 141.1, 140.8, 136.4, 133.7, 129.9, 129.8, 128.8, 128.7, 128.3, 127.6, 119.9, 117.9, 114.3, 106.1, 55.4;

HRMS (APCI) m/z calcd for $C_{23}H_{19}BN_2O$ (M^-): 350.1596, found: 350.1599.

4-methoxy-4''-(trifluoromethyl)-1,1'3',1''-terphenyl (4)[4]

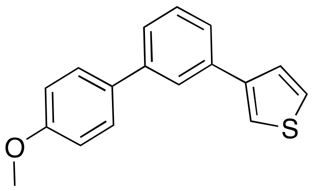

A round-bottomed flask was charged with 2-(4'-methoxy-[1,1'-biphenyl]-3-yl)-2,3-dihydro-1*H*-naphtho[1,8-*de*][1,3,2]diazaborinine (140.0 mg, 0.4 mmol) and THF (4 mL), and the reaction mixture was stirred for 5 min at room temperature. Then 1N HCl (4.0 mL, 0.4 mmol) was added. The mixture was stirred for another 12 h at room temperature and washed with Et_2O. the aqueous layer was washed twice with Et_2O. The aqueous layer was adjusted to pH 12 by aqueous NaOH and washed by Et_2O to remove 1,8-diaminonaphthalene. The aqueous layer was neutralized with HCl and extracted with EtOAc three times. The combined EtOAc layers were dried over anhydrous Na_2SO_4, and

the solvent was removed under reduced pressure to give the corresponding boronic acid, which was used for the next reaction without further purification.

An oven dried test tube was charged with the corresponding boronic acid (57.0 mg, 0.25 mmol), 3-bromothiophene (81.0 mg, 0.50 mmol), $PdCl_2$ (dppf)·CH_2Cl_2 (21 mg, 0.025 mmol) and K_3PO_4 (160 mg, 0.76 mmol). The reaction tube was evacuated and backfilled with argon, and 1,4-dioxane (1.3 mL) was added via a syringe. The reaction mixture was stirred for 5h at 80 °C. The reaction mixture was diluted with EtOAc, and filtered through a pad of celite, and the filtrate was concentrated under reduced pressure. The crude product was purified by column chromatography by PE/EA (10:1) to get the target product (54.5 mg, 82%).

Melting point: 126.4~127.8 °C;

IR (cm^{-1}): 3096.2, 3004.0, 2954.8, 2837.2, 1603.2, 1513.4, 1441.4;

^{1}H NMR (400 MHz, $CDCl_3$) δ 7.78 (s, 1H), 7.47 (m, 8H), 7.01 (d, *J* = 8.6 Hz, 2H), 3.87 (s, 3H);

^{13}C NMR (100 MHz, $CDCl_3$) δ 159.3, 142.4, 141.5, 136.4, 133.7, 129.2, 128.3, 126.5, 126.2, 125.7, 125.0, 124.9, 120.5, 114.3, 55.4;

HRMS (APCI) m/z calcd for $C_{17}H_{15}OS$ [$(M+H)^+$]: 267.0838, found: 267.0841.

References:

1) Iwadate, N.; Suginome, M. Differentially Protected Diboron for Regioselective Diboration of Alkynes: Internal-Selective Cross-Coupling of 1-Alkene-1,2-diboronic Acid Derivatives. *J. Am. Chem. Soc.* **2010**, *132*, 2548-2549.
2) Yoshida, H.; Takemotoa, Y.; Takakia K. A masked diboron in Cu-catalysed borylation reaction: highly regioselective formal hydroboration of alkynes for synthesis of branched alkenylborons. *Chem. Commun.* **2014**, *50*, 8299-8302.
3) Noguchi, H.; Shioda, T.; Chou, C.; Suginome, M. Differentially Protected Benzenediboronic Acids: Divalent Cross-Coupling Modules for the Efficient Synthesis of Boron-Substituted Oligoarenes. *Org. Chem.* **2008**, *10*, 377-380.
4) Seath, C. P.; Fyfe, J. W. B.; Molloy, J. J.; Watson, A. J. B. Tandem Chemoselective Suzuki-Miyaura Cross-Coupling Enabled by Nucleophile Speciation Control. *Angew. Chem. Int. Ed.* **2015**, *54*, 9976-9979.

2. Copy of NMR spectra

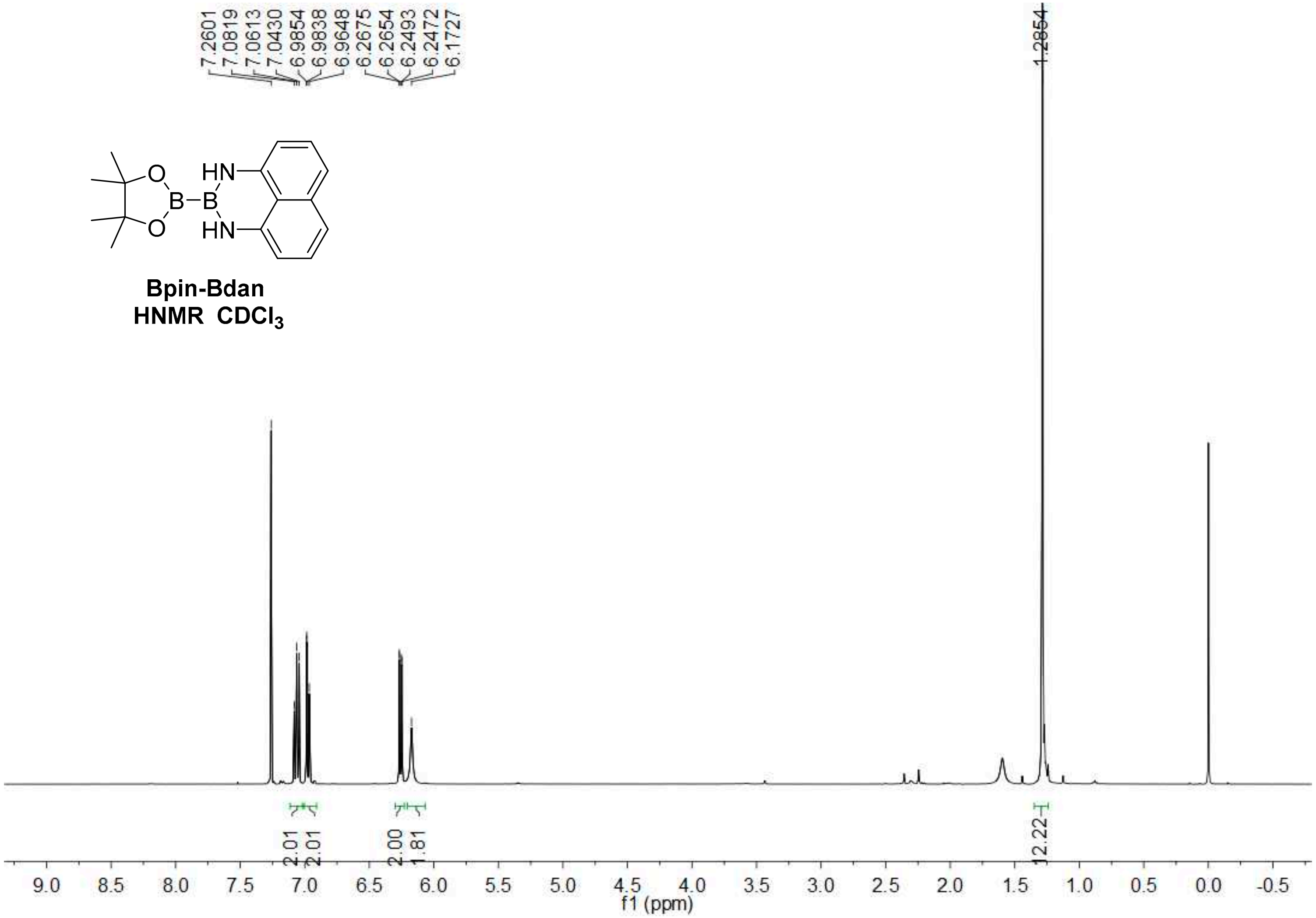

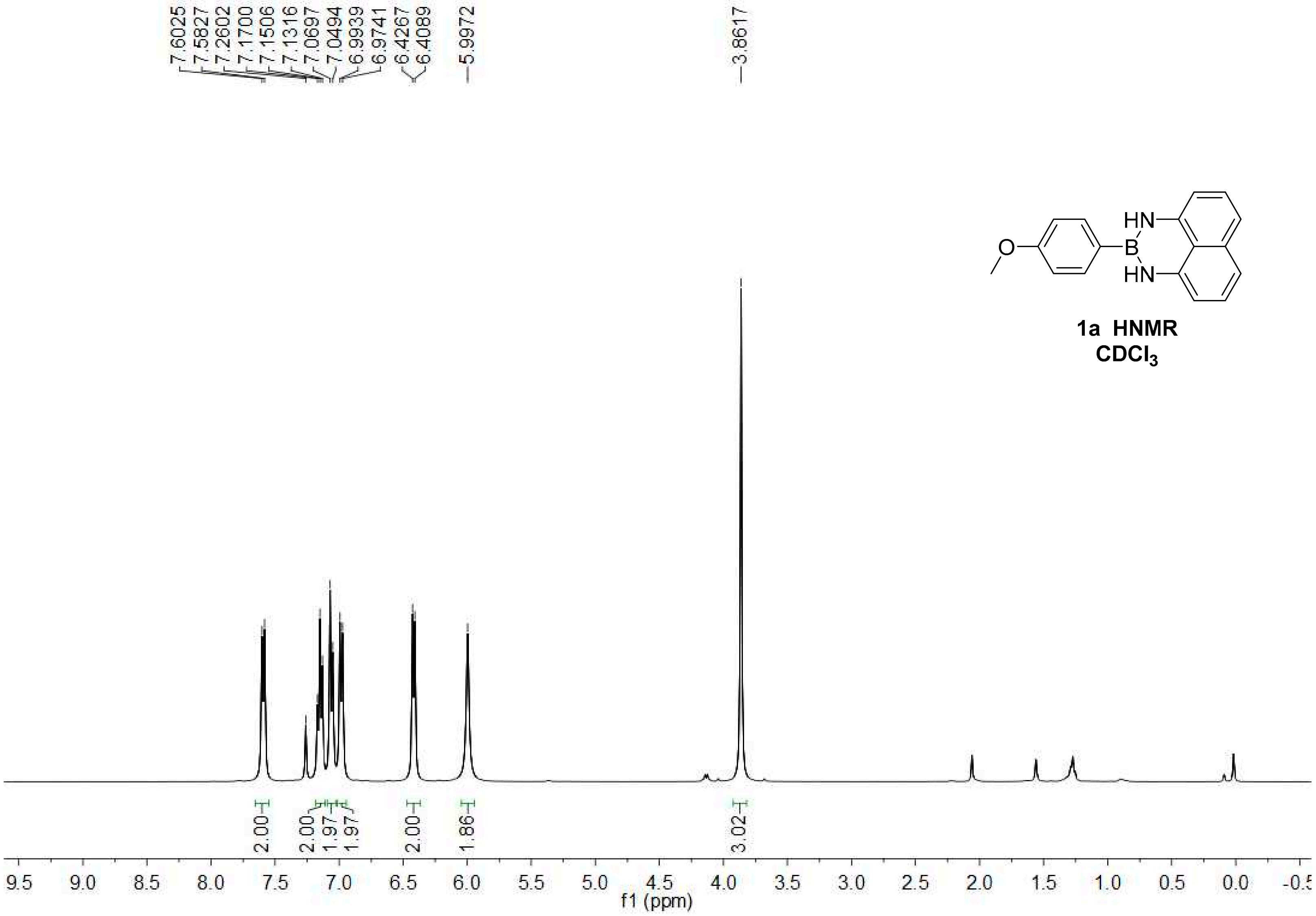
7.6025
7.5827
7.2602
7.1700
7.1506
7.1316
7.0697
7.0494
6.9939
6.9741
6.4267
6.4089
5.9972
3.8617
HN
B
HN
O
1a HNMR
$CDCl_3$
2.00
2.00
1.97
1.97
2.00
1.86
3.02
9.5 9.0 8.5 8.0 7.5 7.0 6.5 6.0 5.5 5.0 4.5 4.0 3.5 3.0 2.5 2.0 1.5 1.0 0.5 0.0 -0.5
f1 (ppm)

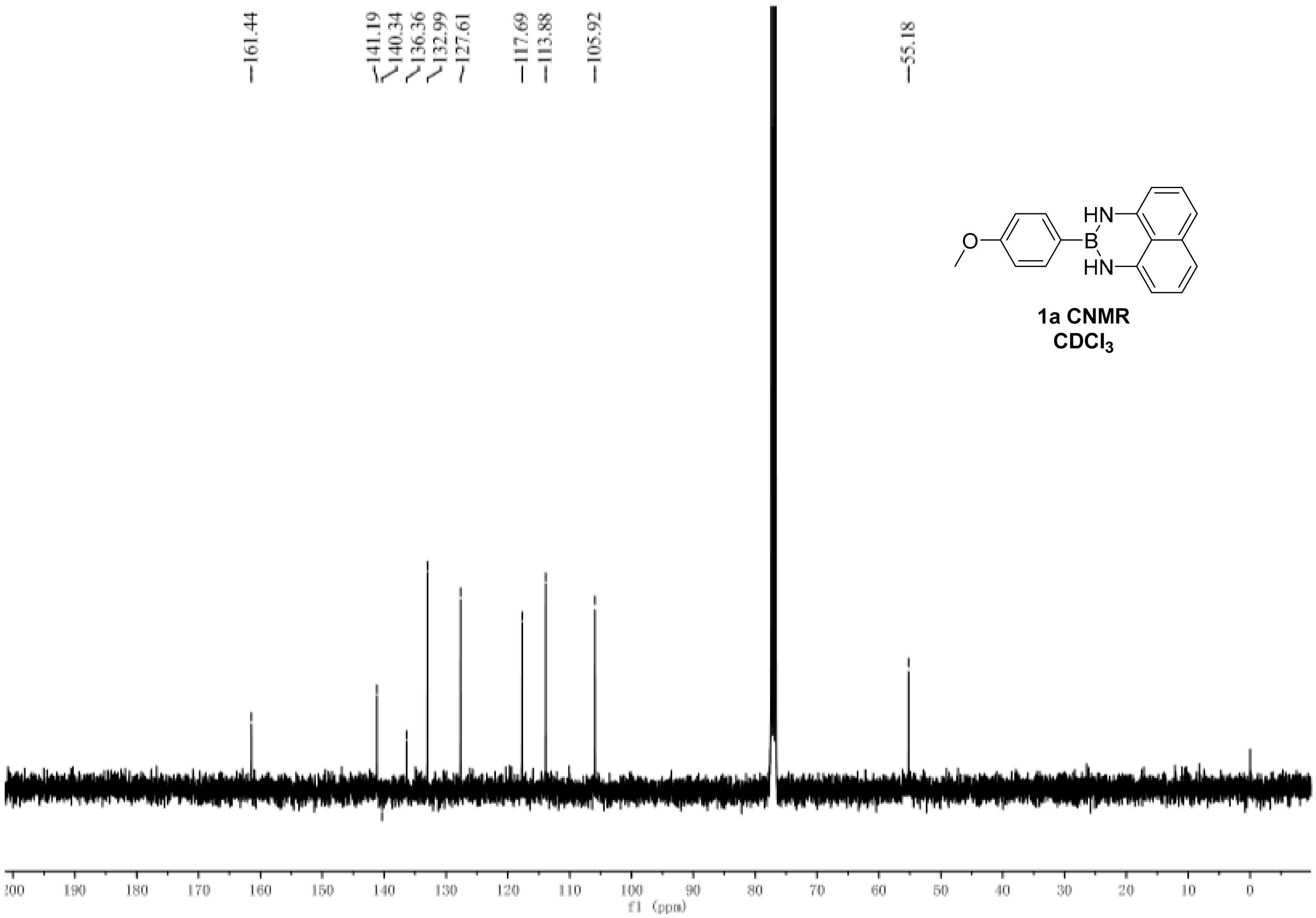

1a CNMR
$CDCl_3$

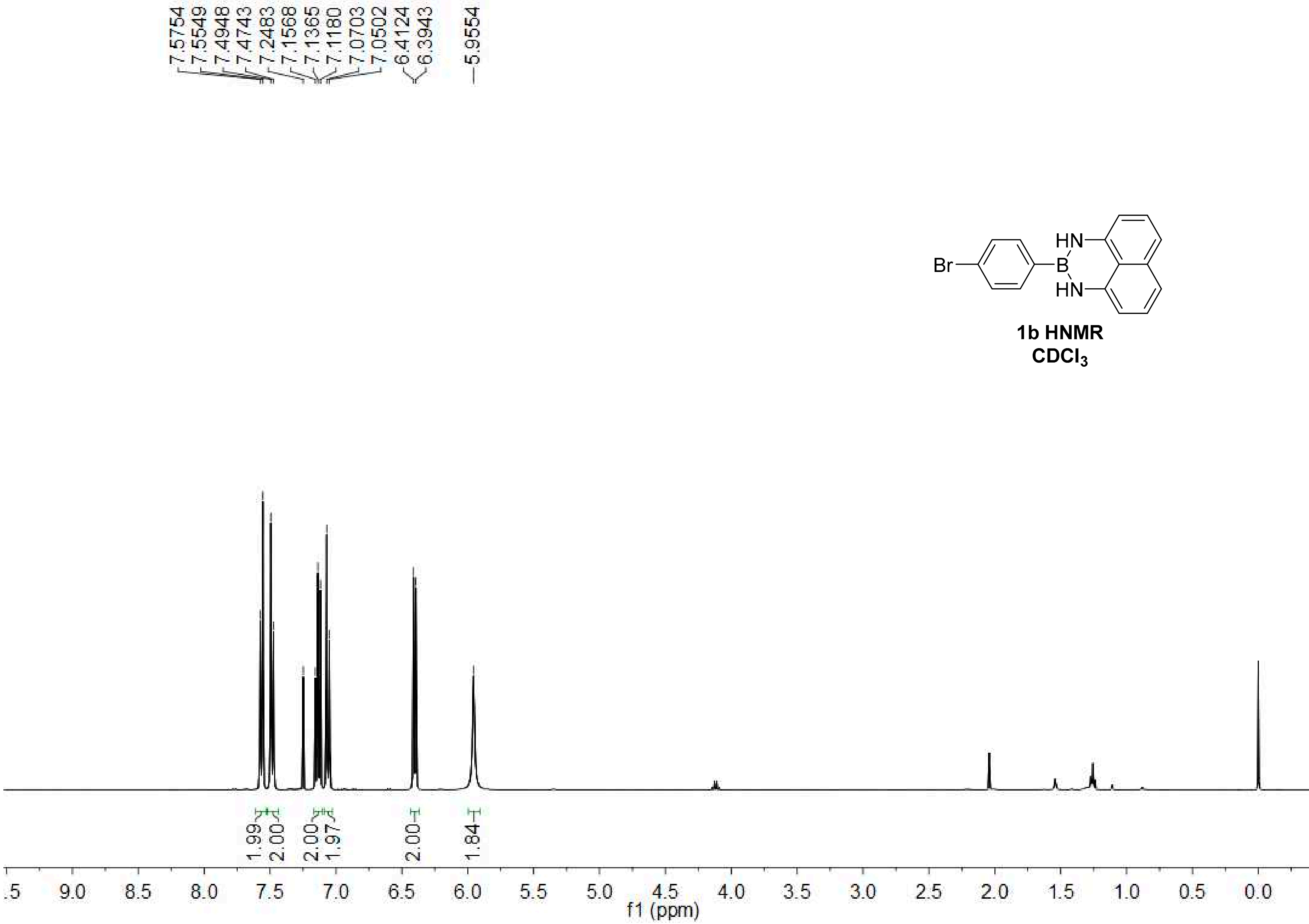

1b HNMR
$CDCl_3$

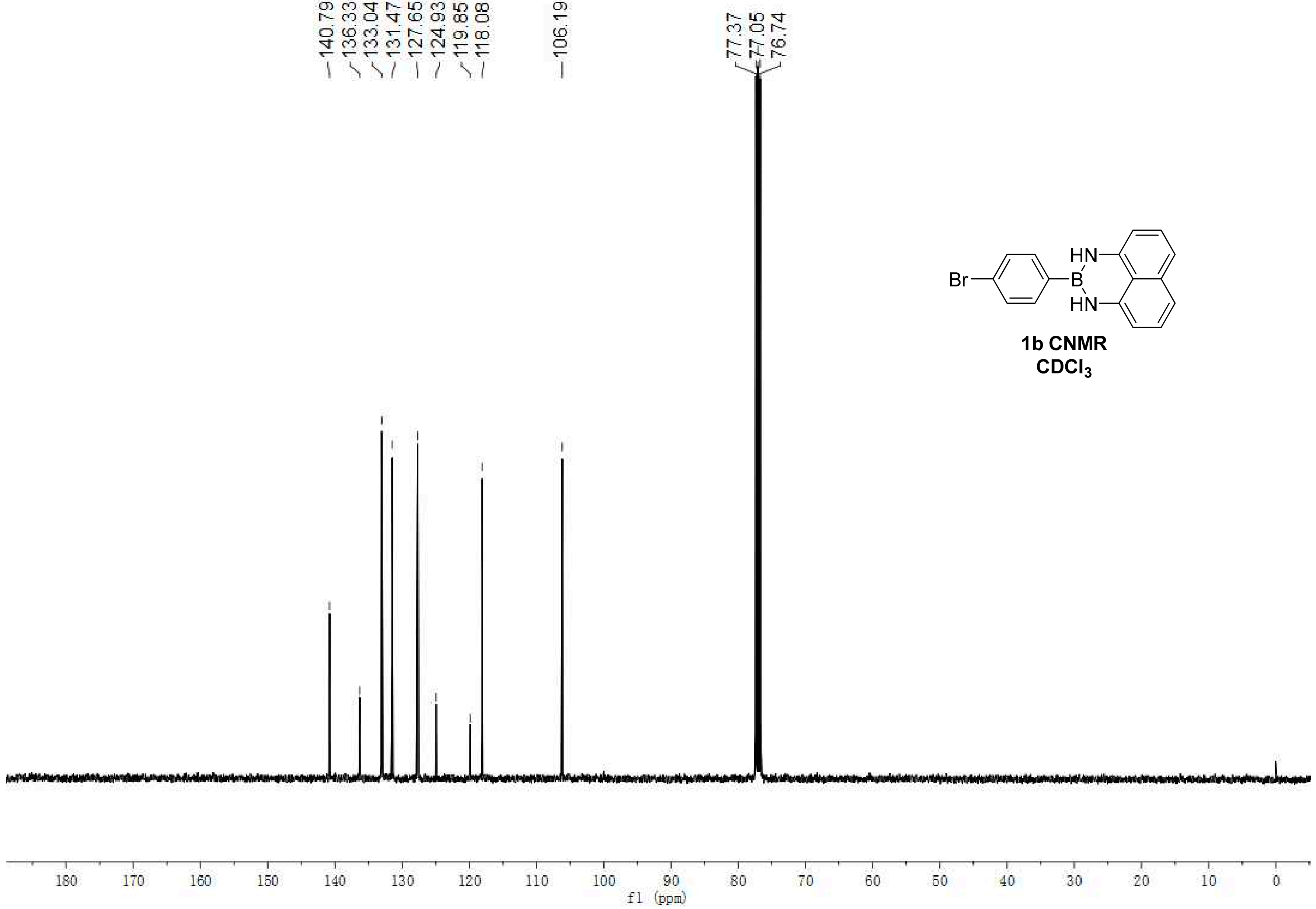
140.79
136.33
133.04
131.47
127.65
124.93
119.85
118.08
106.19
77.37
77.05
76.74
Br
HN
B
HN
1b CNMR
CDCl3
180
170
160
150
140
130
120
110
100
90
80
70
60
50
40
30
20
10
0
f1 (ppm)

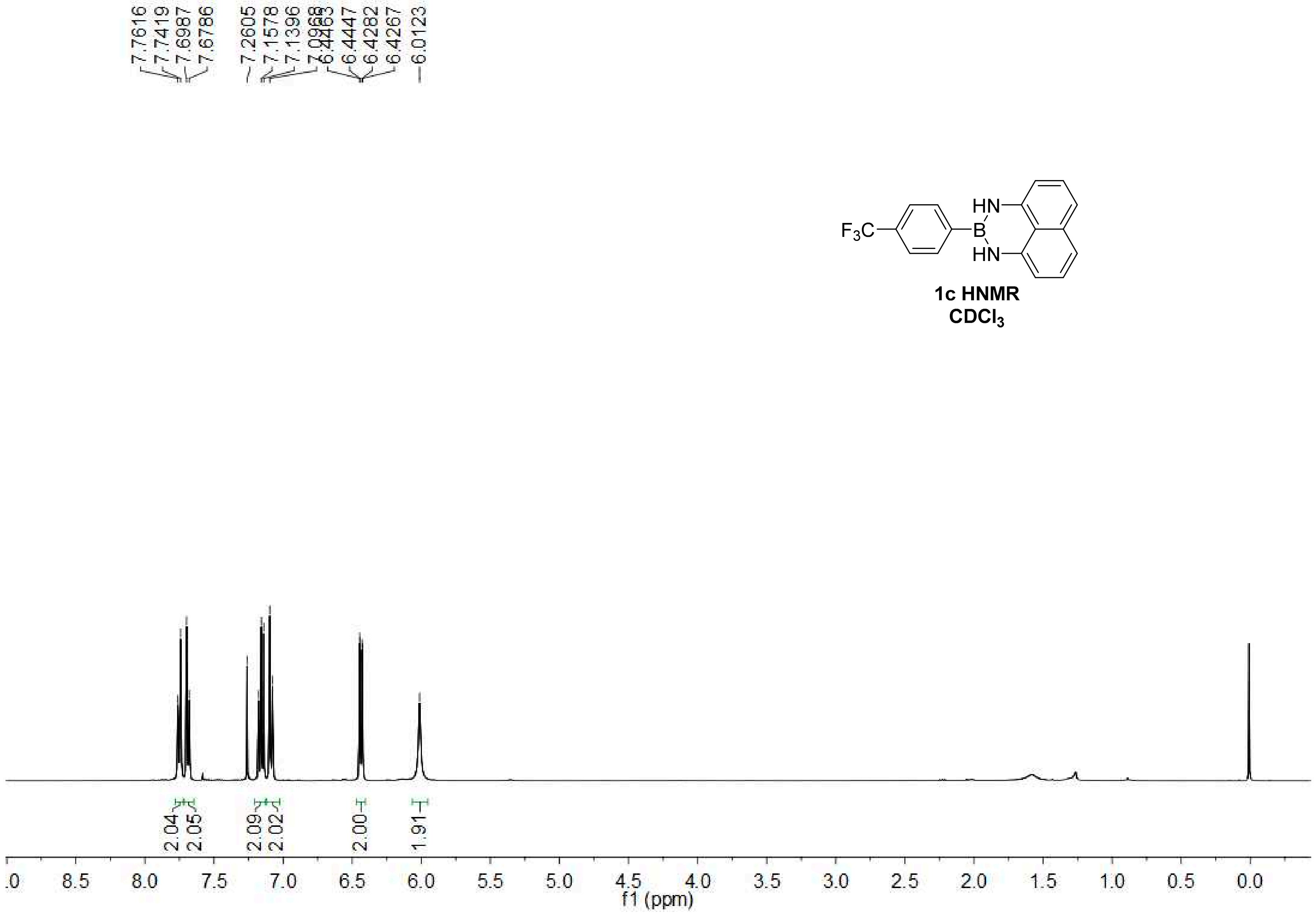

7.7616
7.7419
7.6987
7.6786
7.2605
7.1578
7.1396
6.4463
6.4447
6.4282
6.4267
6.0123
F3C
HN
B
HN
1c HNMR
CDCl3
2.04
2.05
2.09
2.02
2.00
1.91
8.5
8.0
7.5
7.0
6.5
6.0
5.5
5.0
4.5
4.0
3.5
3.0
2.5
2.0
1.5
1.0
0.5
0.0
f1 (ppm)

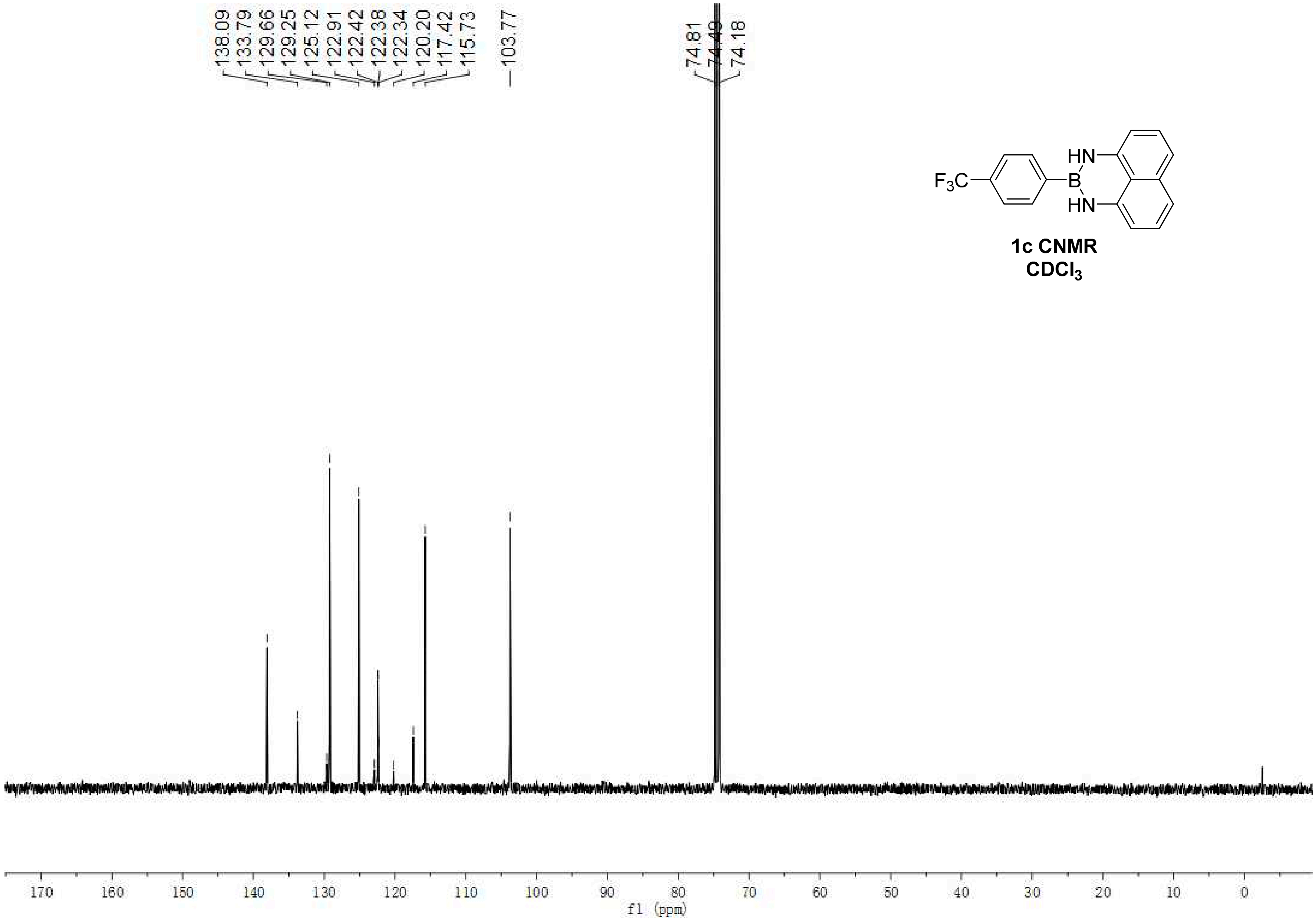
138.09
133.79
129.66
129.25
125.12
122.91
122.42
122.38
122.34
120.20
117.42
115.73
103.77
74.81
74.49
74.18
F3C
HN
B
HN
1c CNMR
CDCl3
170
160
150
140
130
120
110
100
90
80
70
60
50
40
30
20
10
0
f1 (ppm)

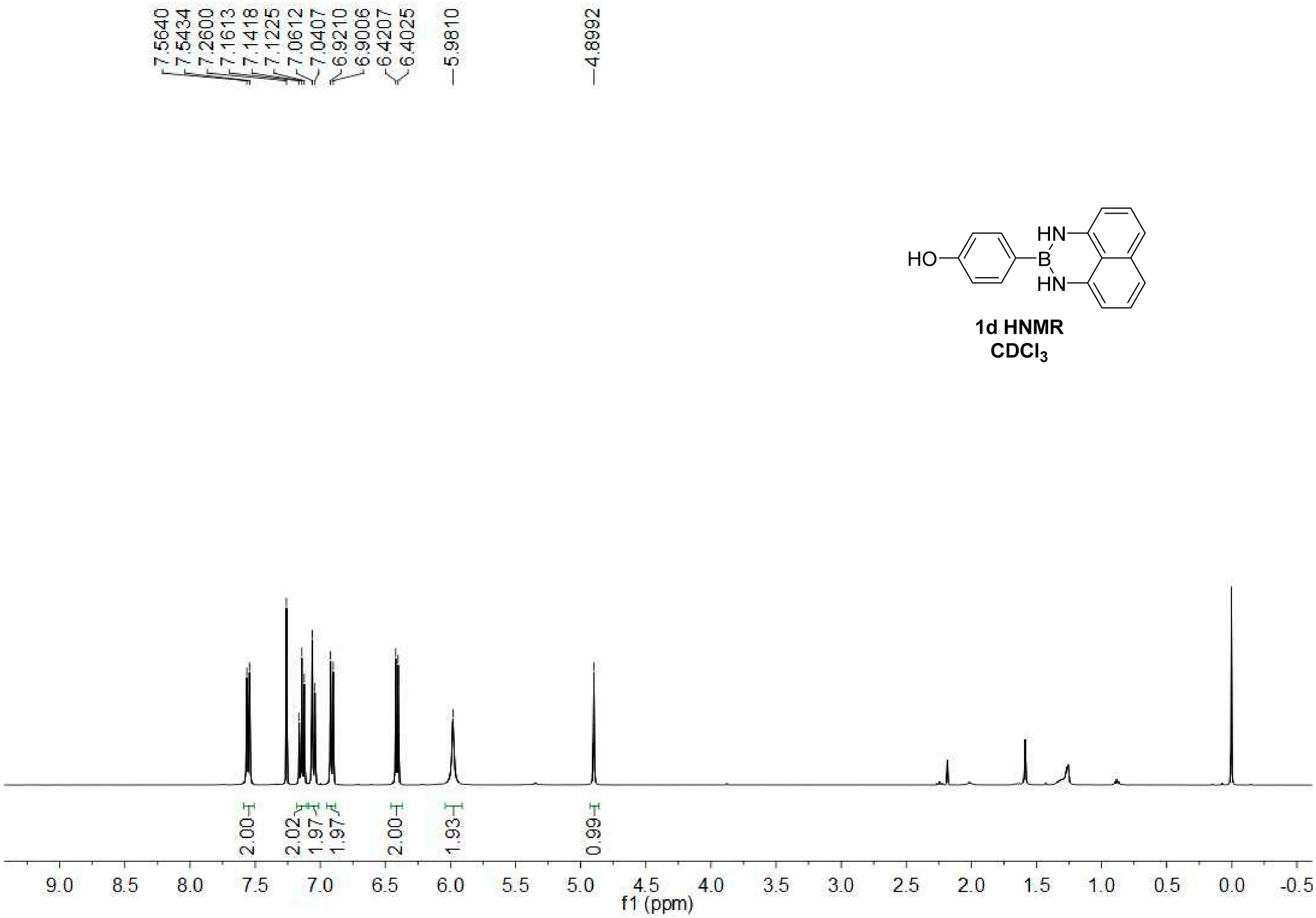
7.5640
7.5434
7.2600
7.1613
7.1418
7.1225
7.0612
7.0407
6.9210
6.9006
6.4207
6.4025
5.9810
4.8992
HO
HN
B
HN
1d HNMR
CDCl3
2.00
2.02
1.97
1.97
2.00
1.93
0.99
9.0 8.5 8.0 7.5 7.0 6.5 6.0 5.5 5.0 4.5 4.0 3.5 3.0 2.5 2.0 1.5 1.0 0.5 0.0 -0.5
f1 (ppm)

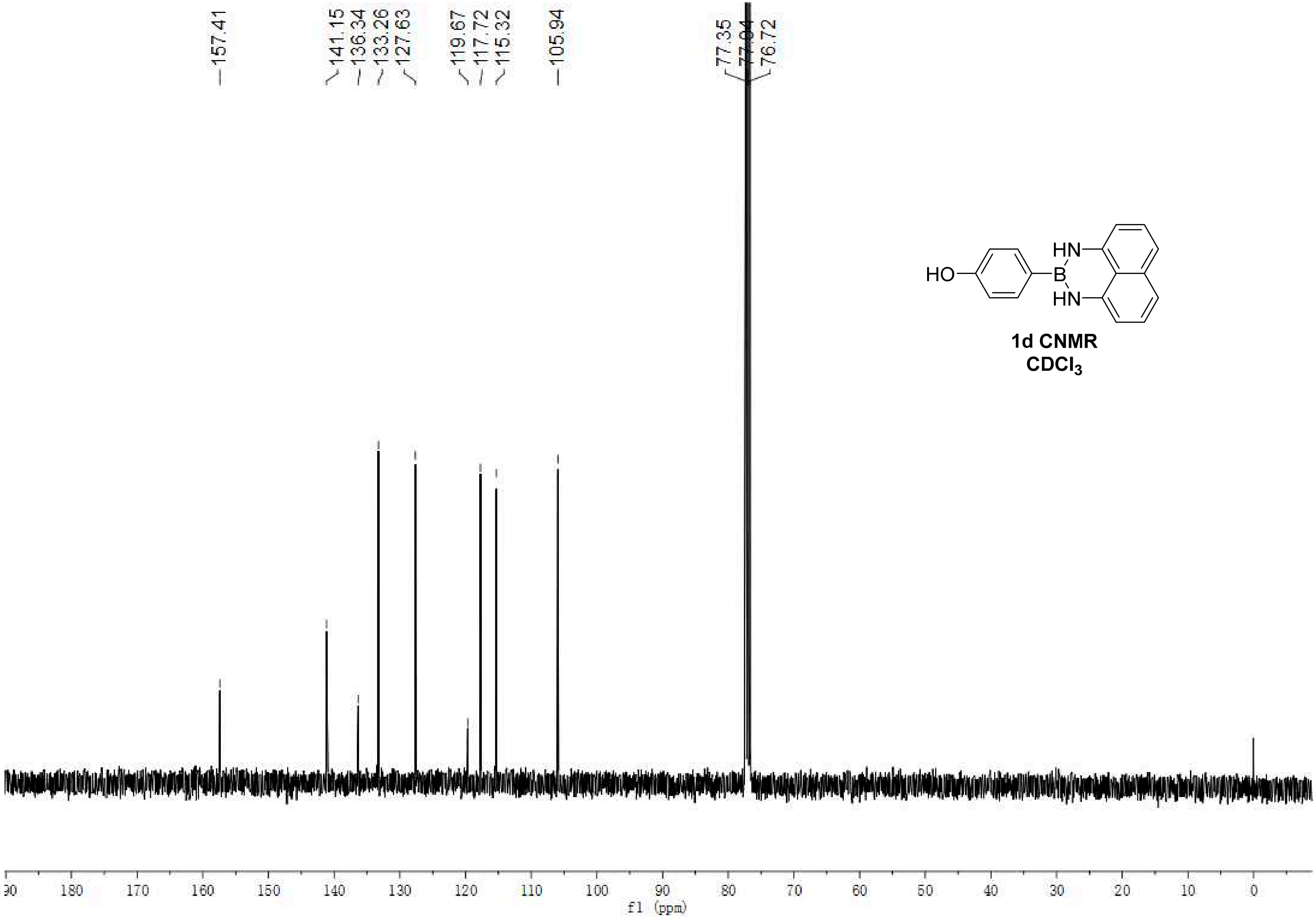
157.41
141.15
136.34
133.26
127.63
119.67
117.72
115.32
105.94
77.35
76.72
HO
HN
B
HN
1d CNMR
CDCl3
180
170
160
150
140
130
120
110
100
90
80
70
60
50
40
30
20
10
0
f1 (ppm)

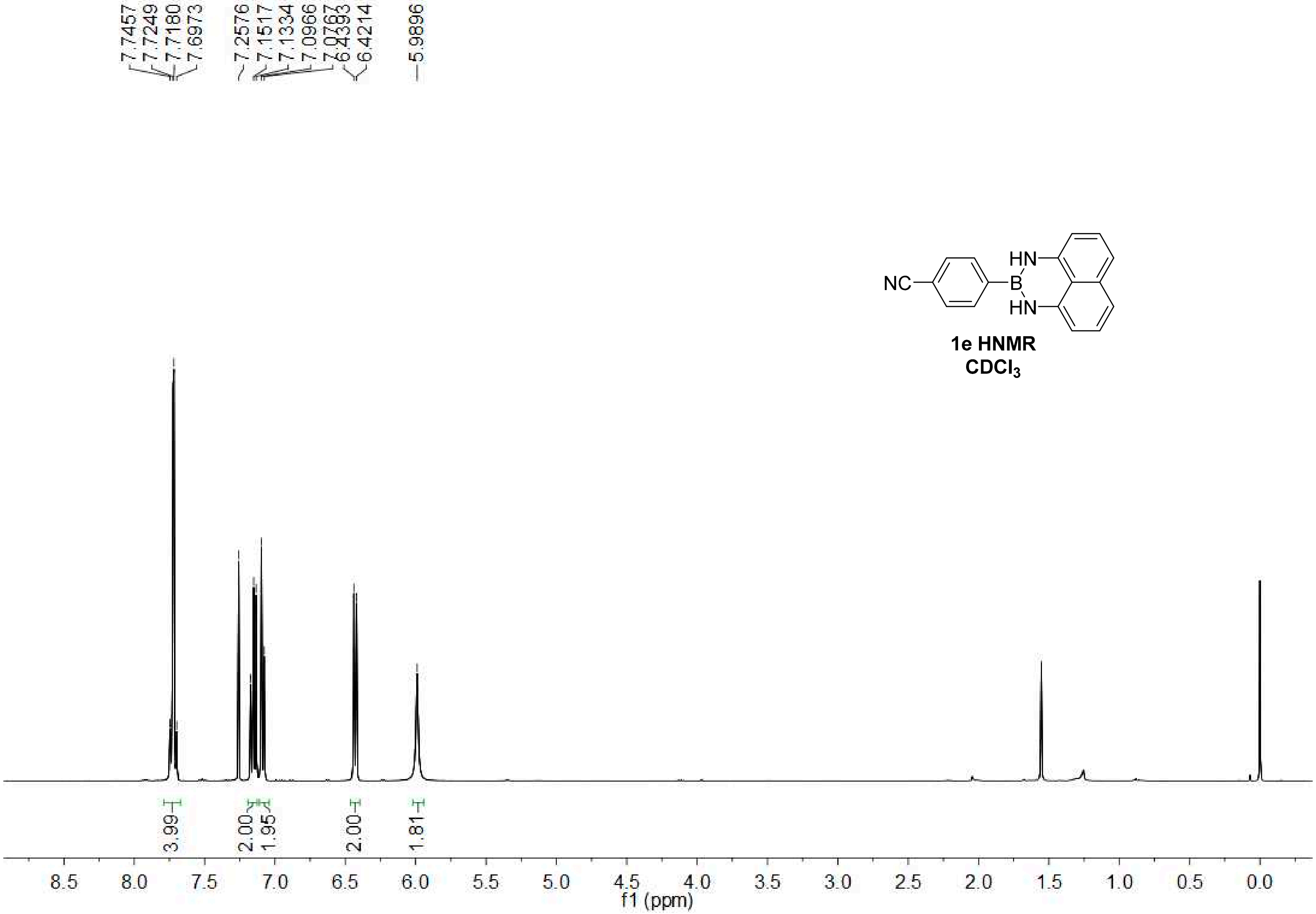
7.7457
7.7249
7.7180
7.6973
7.2576
7.1517
7.1334
7.0966
7.0767
6.4393
6.4214
5.9896
NC
HN
B
HN
1e HNMR
$CDCl_3$
3.99
2.00
1.95
2.00
1.81
8.5
8.0
7.5
7.0
6.5
6.0
5.5
5.0
4.5
4.0
3.5
3.0
2.5
2.0
1.5
1.0
0.5
0.0
f1 (ppm)

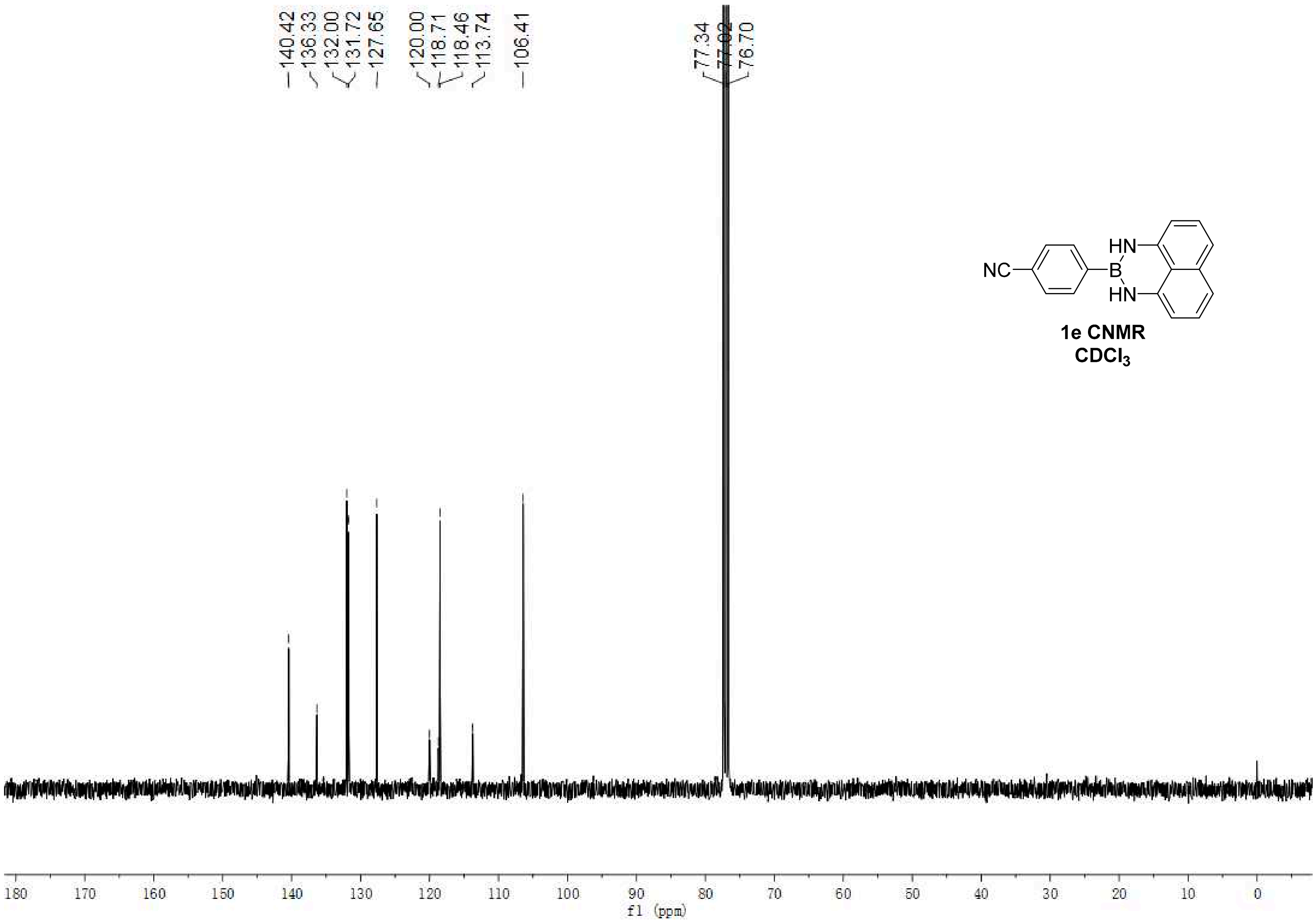
140.42
136.33
132.00
131.72
127.65
120.00
118.71
118.46
113.74
106.41
77.34
77.02
76.70
NC
HN
B
HN
1e CNMR
CDCl3
180
170
160
150
140
130
120
110
100
90
80
70
60
50
40
30
20
10
0
f1 (ppm)

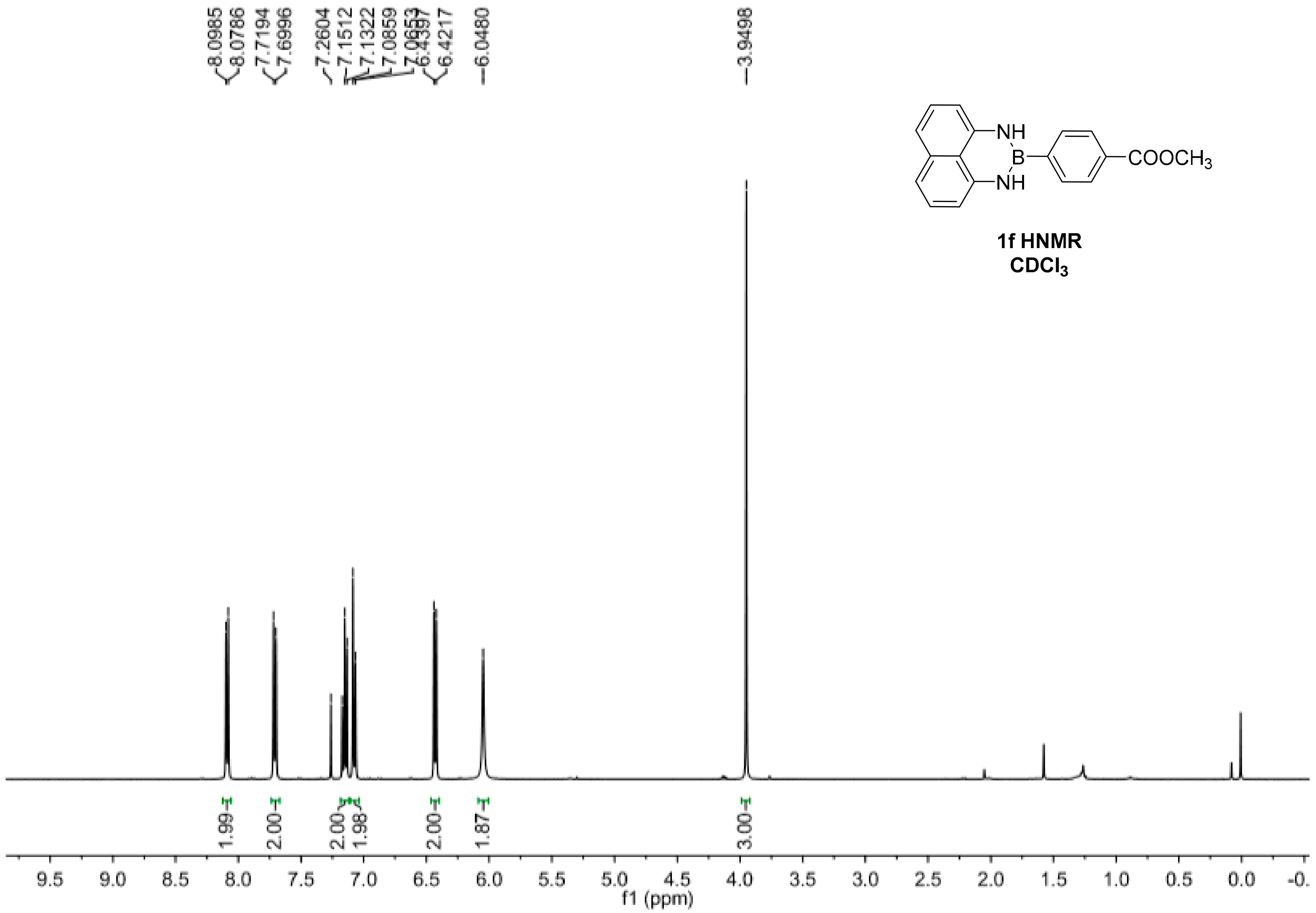
8.0985
8.0786
7.7194
7.6996
7.2604
7.1512
7.1322
7.0859
7.0653
6.4397
6.4217
6.0480
3.9498
NH
B
NH
COOCH3
1f HNMR
CDCl3
1.99
2.00
2.00
1.98
2.00
1.87
3.00
9.5
9.0
8.5
8.0
7.5
7.0
6.5
6.0
5.5
5.0
4.5
4.0
3.5
3.0
2.5
2.0
1.5
1.0
0.5
0.0
f1 (ppm)

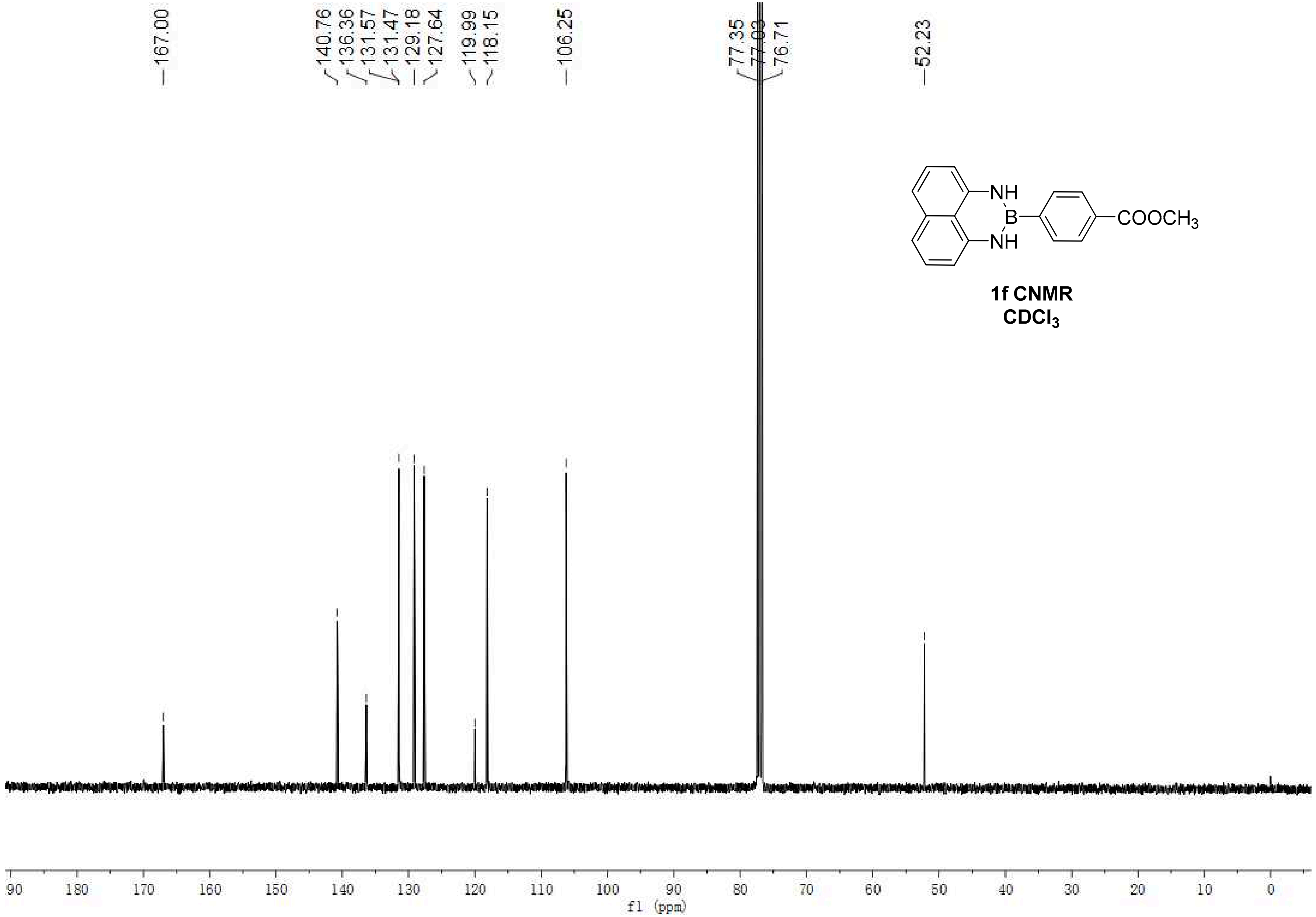
167.00
140.76
136.36
131.57
131.47
129.18
127.64
119.99
118.15
106.25
77.35
76.71
52.23
NH
B
NH
COOCH3
1f CNMR
CDCl3
90 180 170 160 150 140 130 120 110 100 90 80 70 60 50 40 30 20 10 0
f1 (ppm)

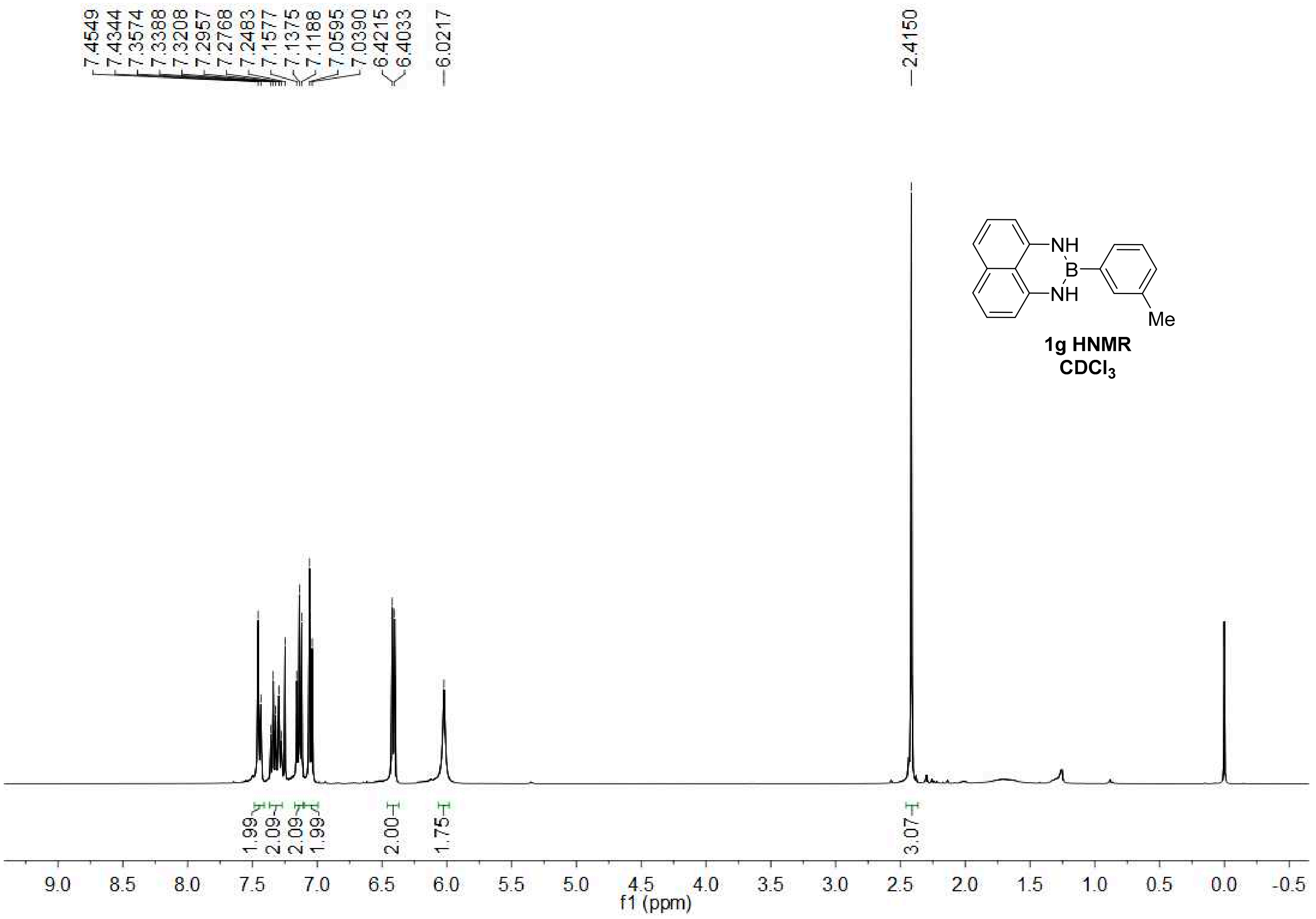
7.4549
7.4344
7.3574
7.3388
7.3208
7.2957
7.2768
7.2483
7.1577
7.1375
7.1188
7.0595
7.0390
6.4215
6.4033
6.0217
2.4150
NH
B
NH
Me
1g HNMR
CDCl3
1.99
2.09
2.09
1.99
2.00
1.75
3.07
9.0
8.5
8.0
7.5
7.0
6.5
6.0
5.5
5.0
4.5
4.0
3.5
3.0
2.5
2.0
1.5
1.0
0.5
0.0
-0.5
f1 (ppm)

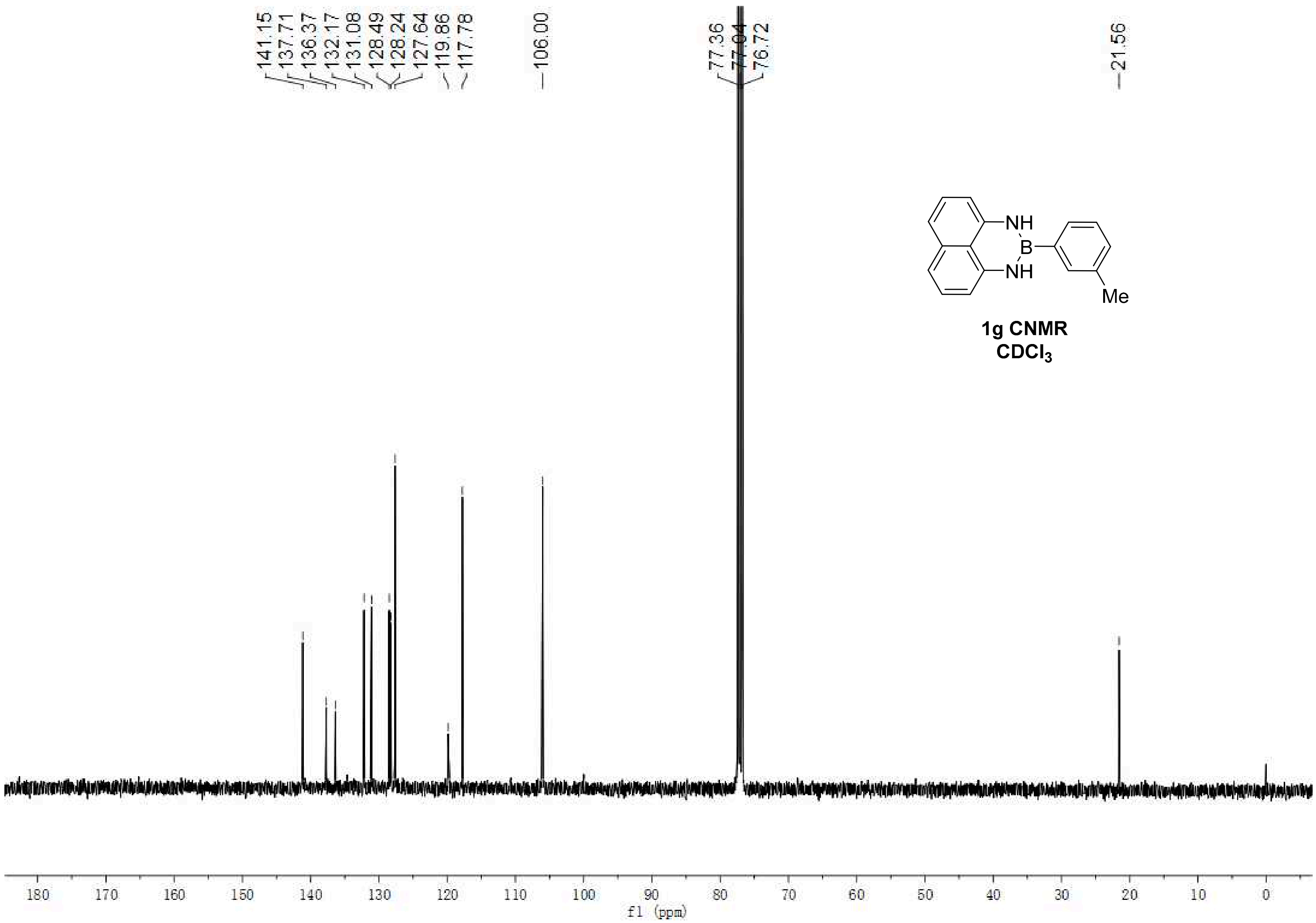
141.15
137.71
136.37
132.17
131.08
128.49
128.24
127.64
119.86
117.78
106.00
77.36
77.04
76.72
21.56
NH
B
NH
Me
1g CNMR
CDCl3
180
170
160
150
140
130
120
110
100
90
80
70
60
50
40
30
20
10
0
f1 (ppm)

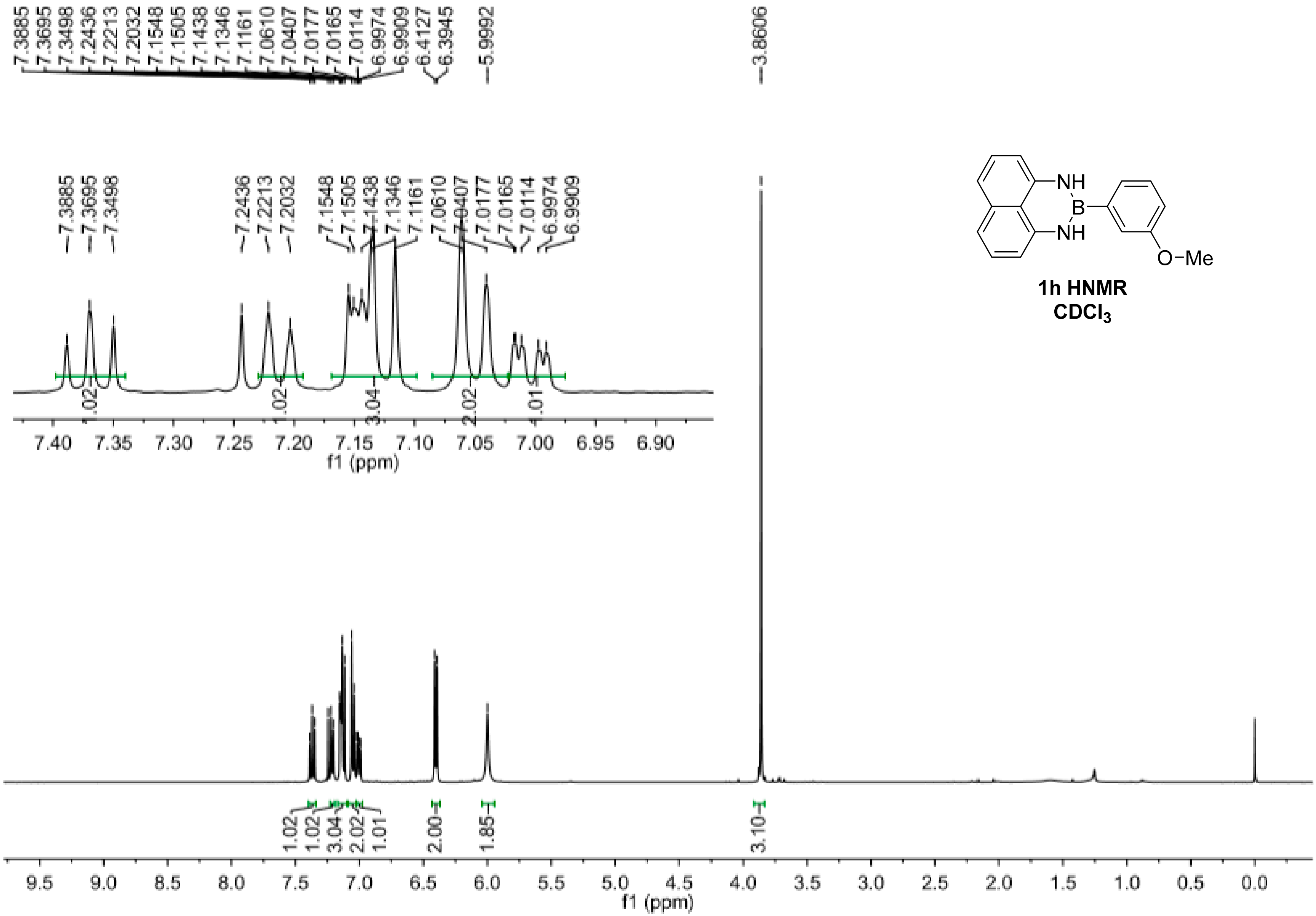
7.3885
7.3695
7.3498
7.2436
7.2213
7.2032
7.1548
7.1505
7.1438
7.1346
7.1161
7.0610
7.0407
7.0177
7.0165
7.0114
6.9974
6.9909
6.4127
6.3945
5.9992
3.8606
NH
B
NH
O−Me
1h HNMR
CDCl3
1.02
1.02
3.04
2.02
1.01
2.00
1.85
3.10
f1 (ppm)

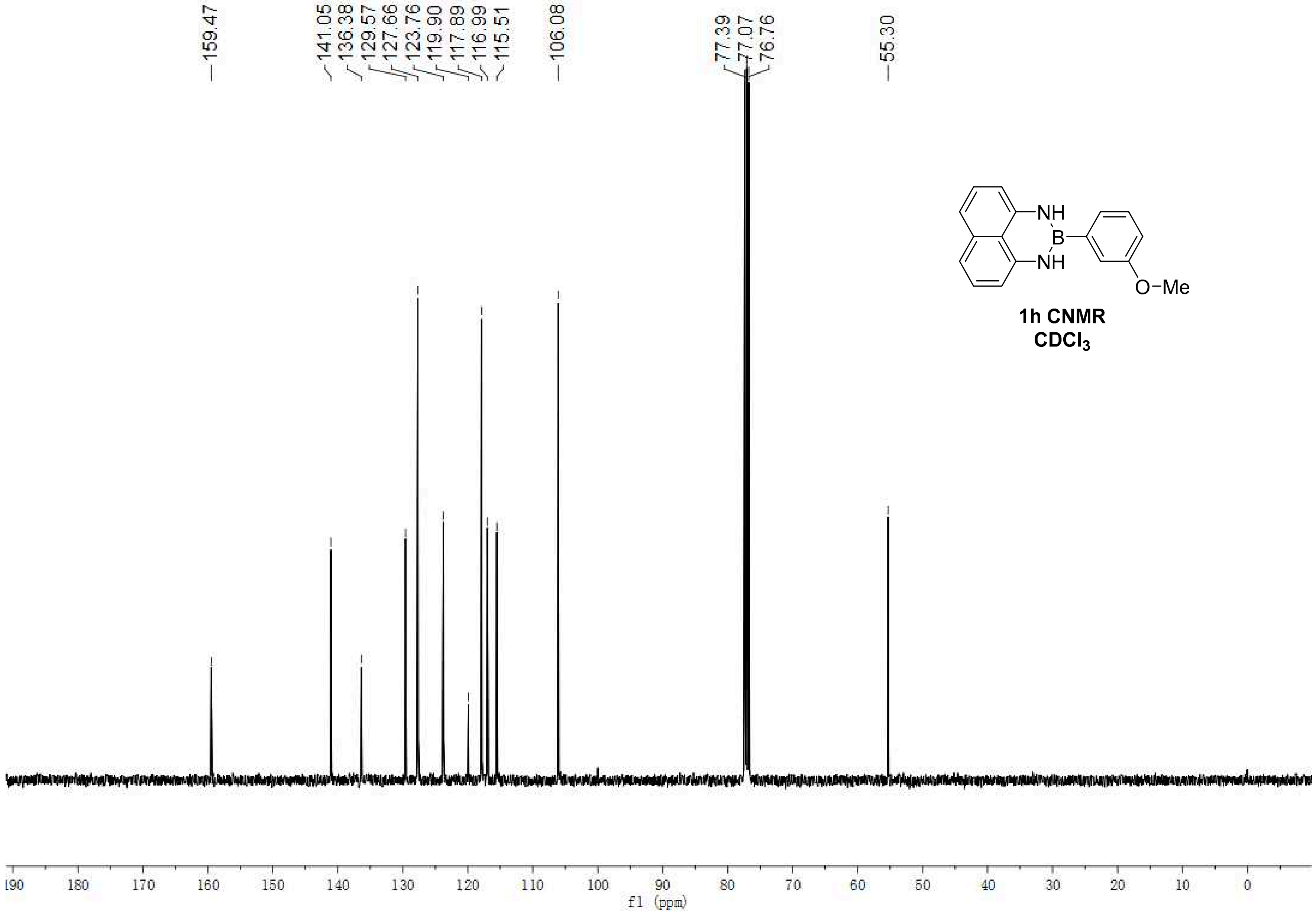
159.47
141.05
136.38
129.57
127.66
123.76
119.90
117.89
116.99
115.51
106.08
77.39
77.07
76.76
55.30
NH
B
NH
O−Me
1h CNMR
CDCl3
f1 (ppm)

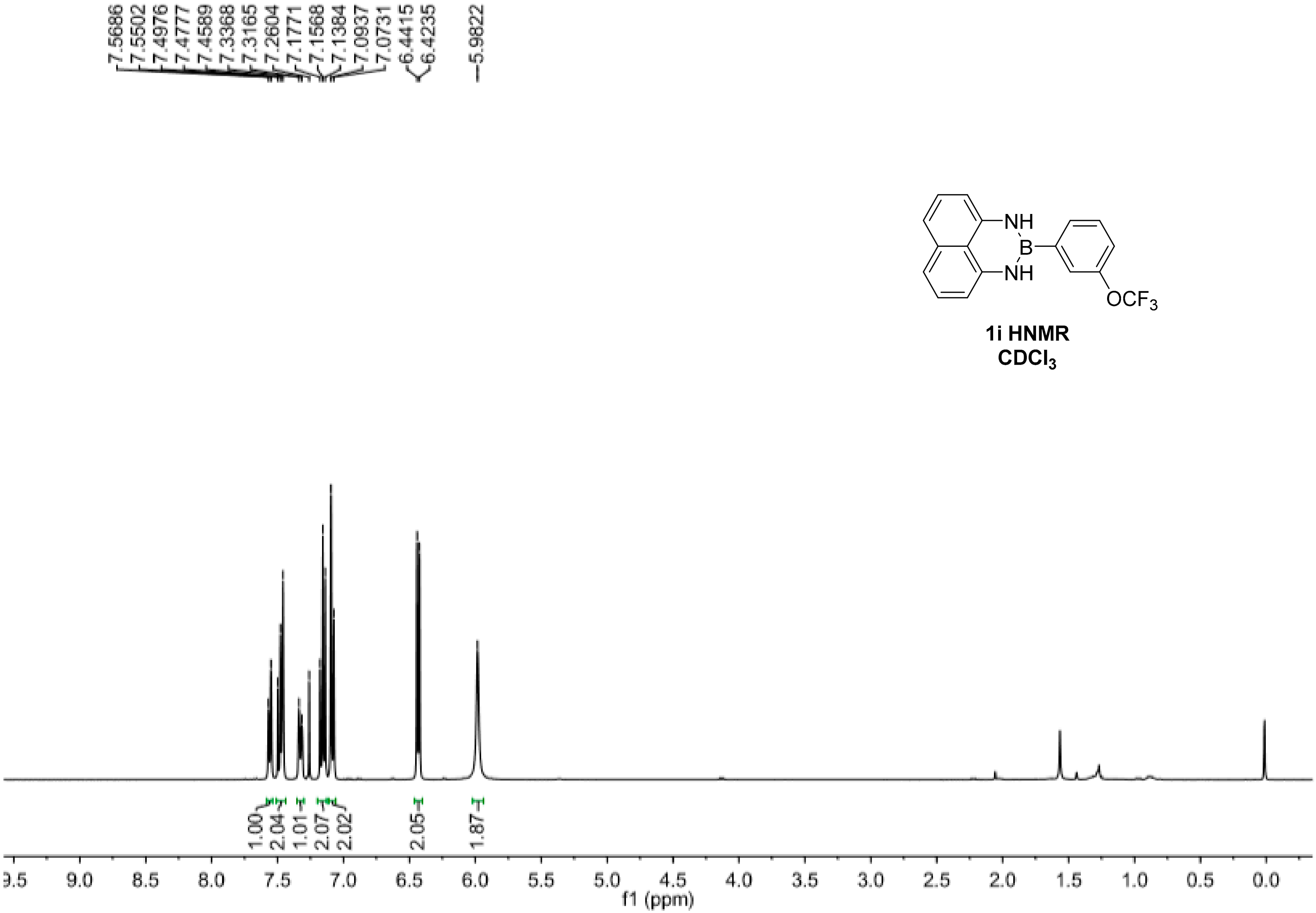
7.5686
7.5502
7.4976
7.4777
7.4589
7.3368
7.3165
7.2604
7.1771
7.1568
7.1384
7.0937
7.0731
6.4415
6.4235
5.9822
NH
B
NH
OCF3
1i HNMR
CDCl3
1.00
2.04
1.01
2.07
2.02
2.05
1.87
9.5
9.0
8.5
8.0
7.5
7.0
6.5
6.0
5.5
5.0
4.5
4.0
3.5
3.0
2.5
2.0
1.5
1.0
0.5
0.0
f1 (ppm)

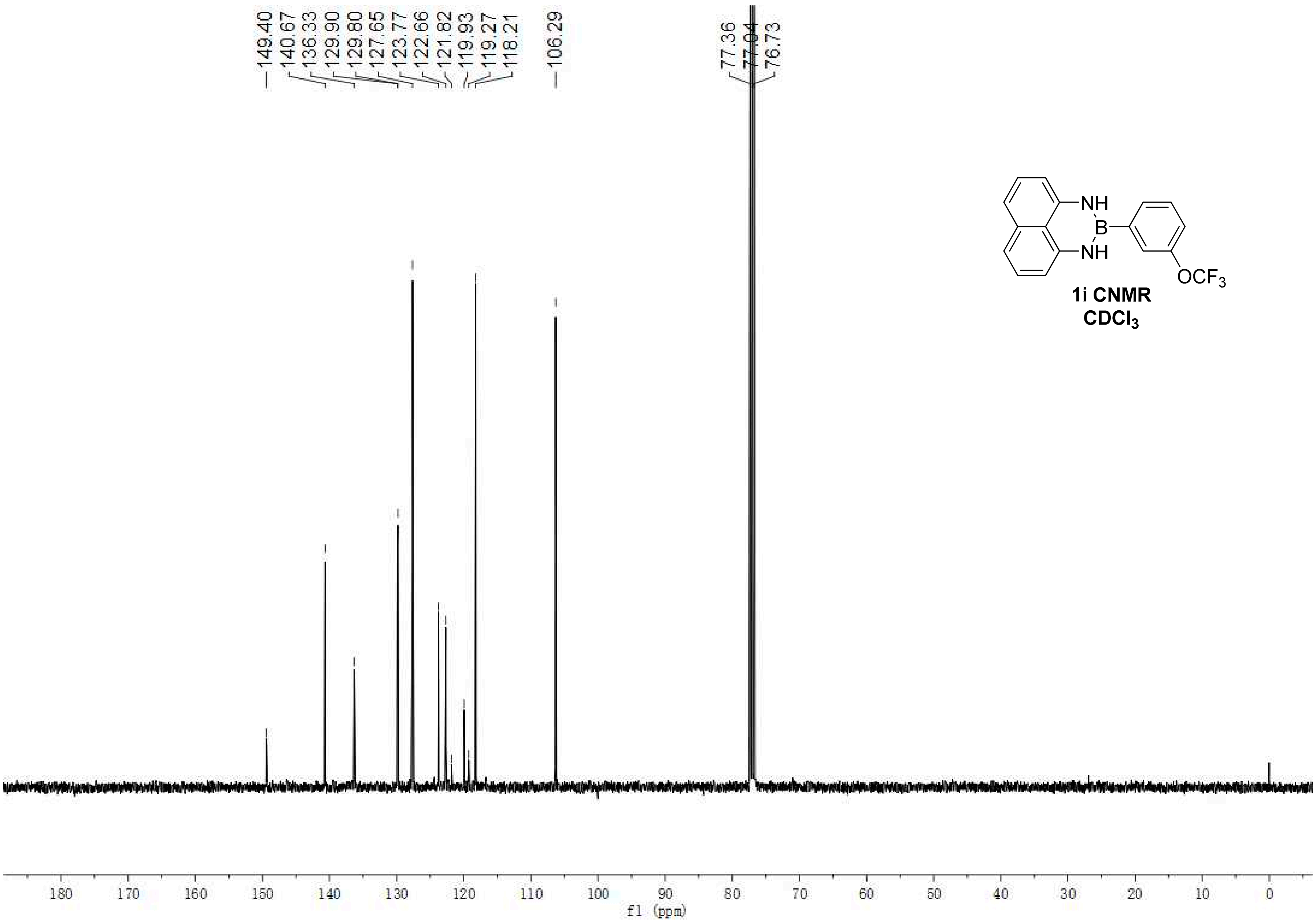
149.40
140.67
136.33
129.90
129.80
127.65
123.77
122.66
121.82
119.93
119.27
118.21
106.29
77.36
77.04
76.73
NH
B
NH
OCF3
1i CNMR
CDCl3
180 170 160 150 140 130 120 110 100 90 80 70 60 50 40 30 20 10 0
f1 (ppm)

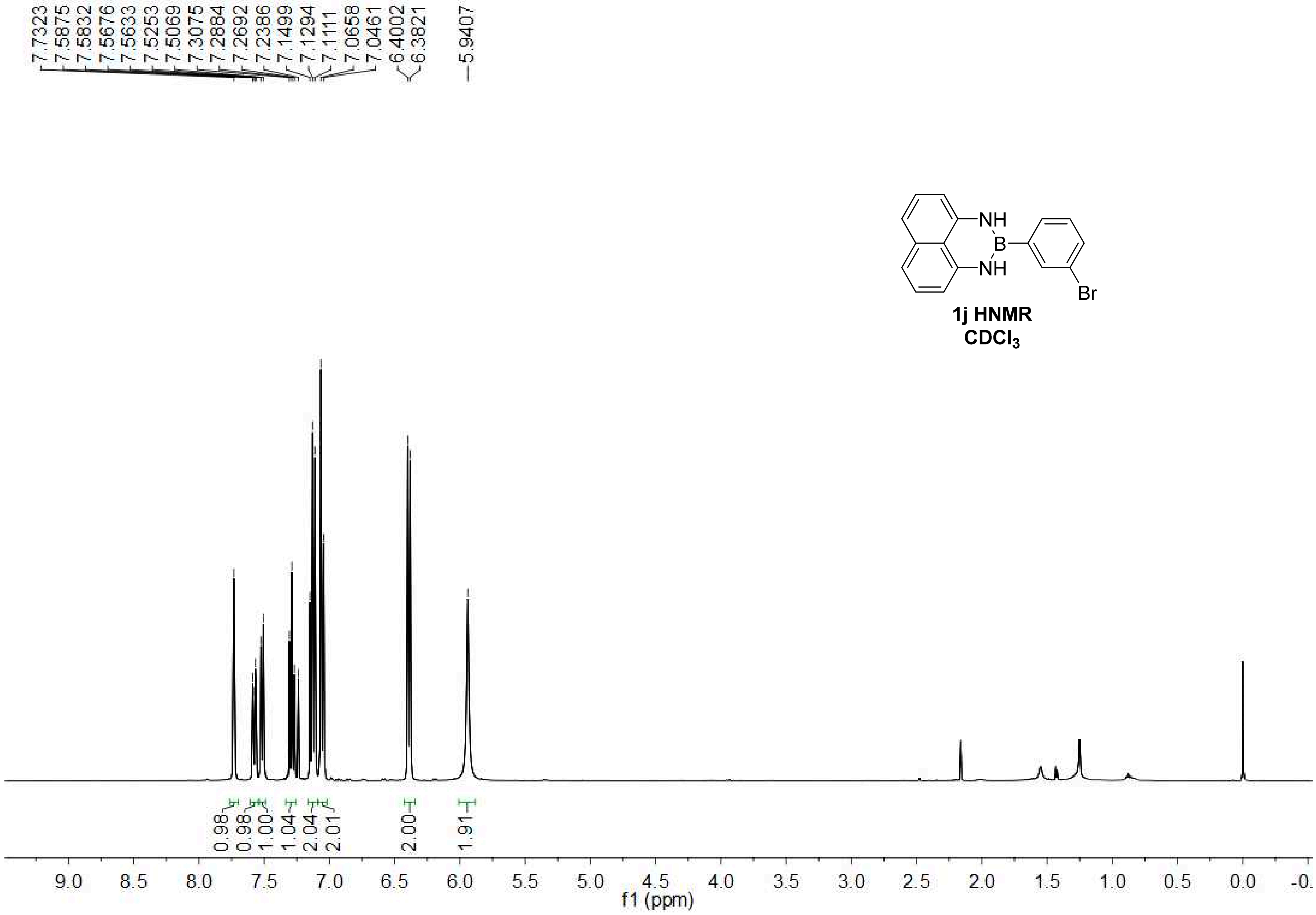
7.7323
7.5875
7.5832
7.5676
7.5633
7.5253
7.5069
7.3075
7.2884
7.2692
7.2386
7.1499
7.1294
7.1111
7.0658
7.0461
6.4002
6.3821
5.9407
NH
B
NH
Br
1j HNMR
CDCl3
0.98
0.98
1.00
1.04
2.04
2.01
2.00
1.91
9.0
8.5
8.0
7.5
7.0
6.5
6.0
5.5
5.0
4.5
4.0
3.5
3.0
2.5
2.0
1.5
1.0
0.5
0.0
f1 (ppm)

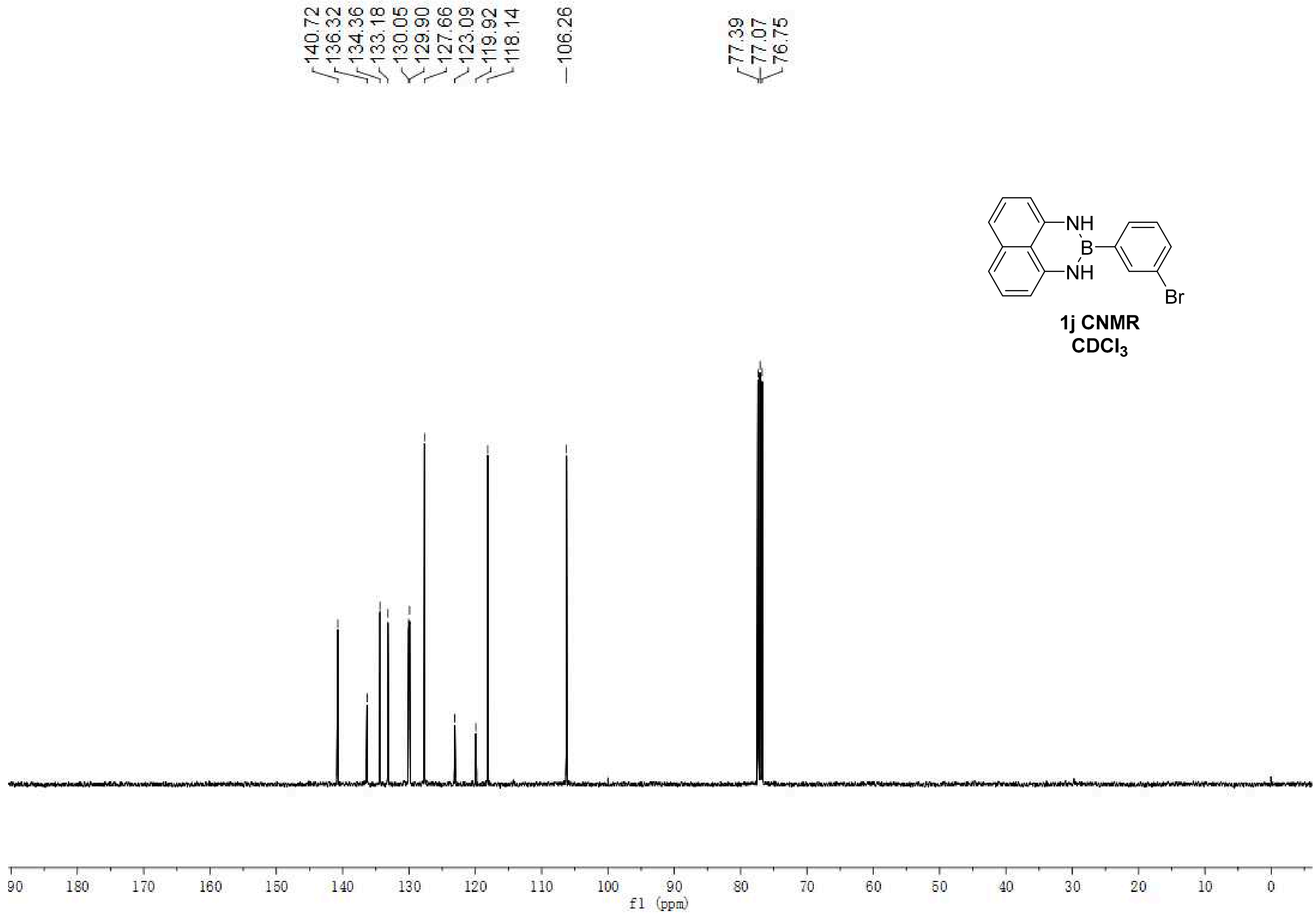
140.72
136.32
134.36
133.18
130.05
129.90
127.66
123.09
119.92
118.14
106.26
77.39
77.07
76.75
NH
B
NH
Br
1j CNMR
CDCl3
90 180 170 160 150 140 130 120 110 100 90 80 70 60 50 40 30 20 10 0
f1 (ppm)

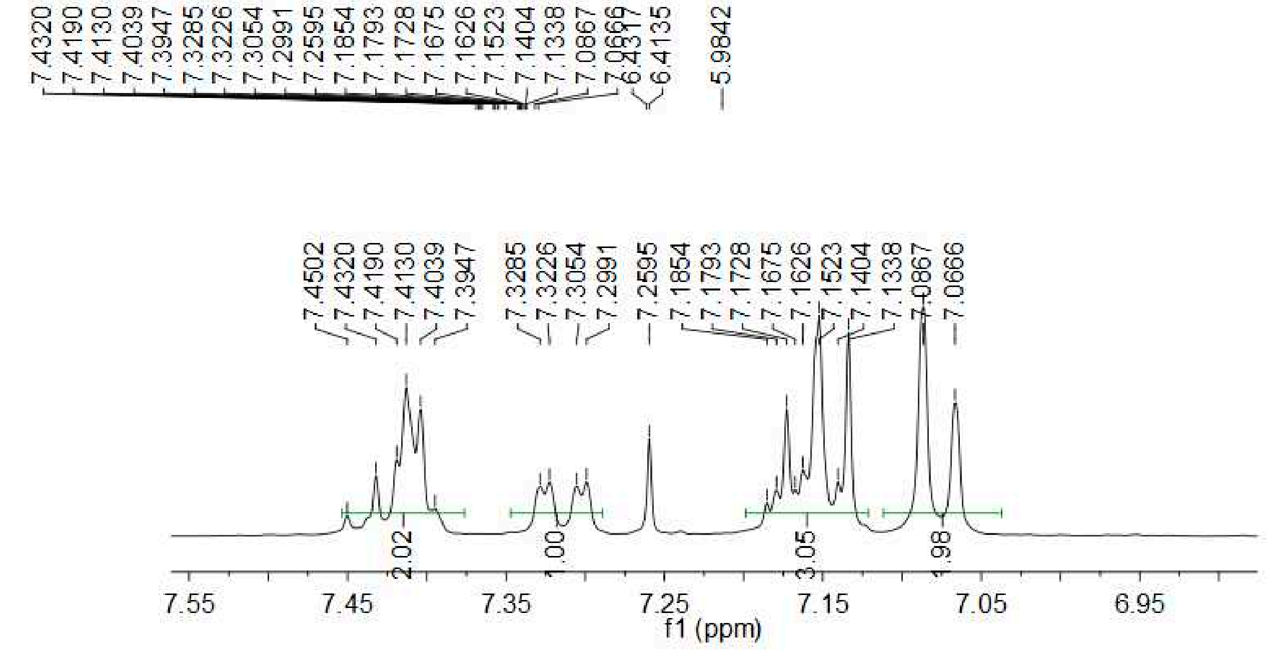

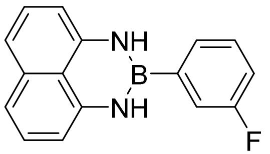

1k HNMR
$CDCl_3$

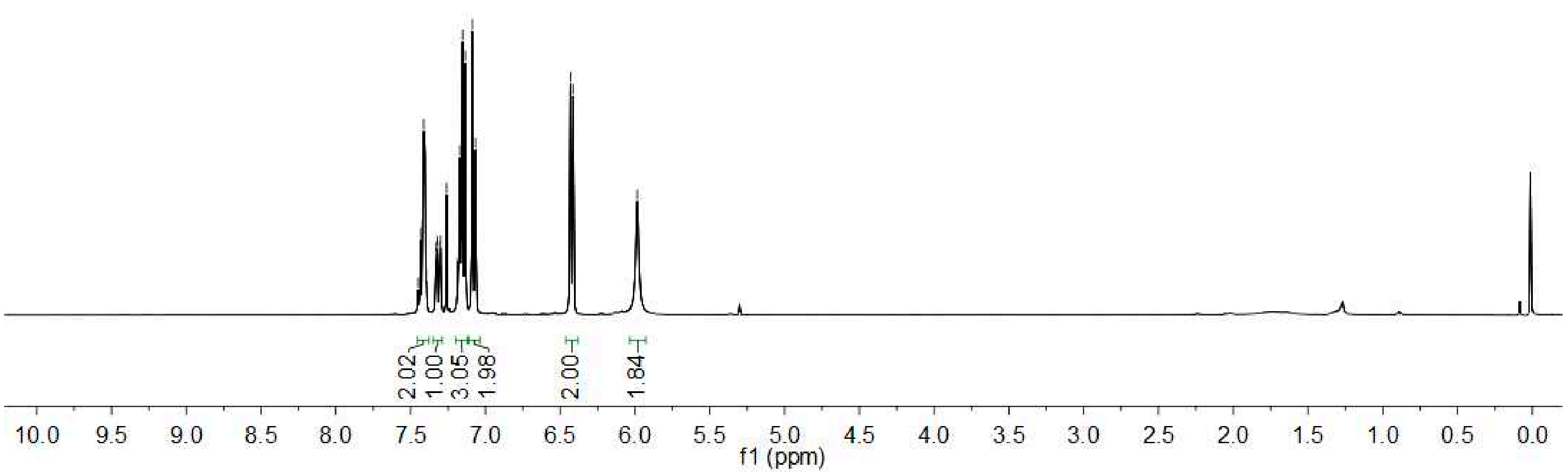

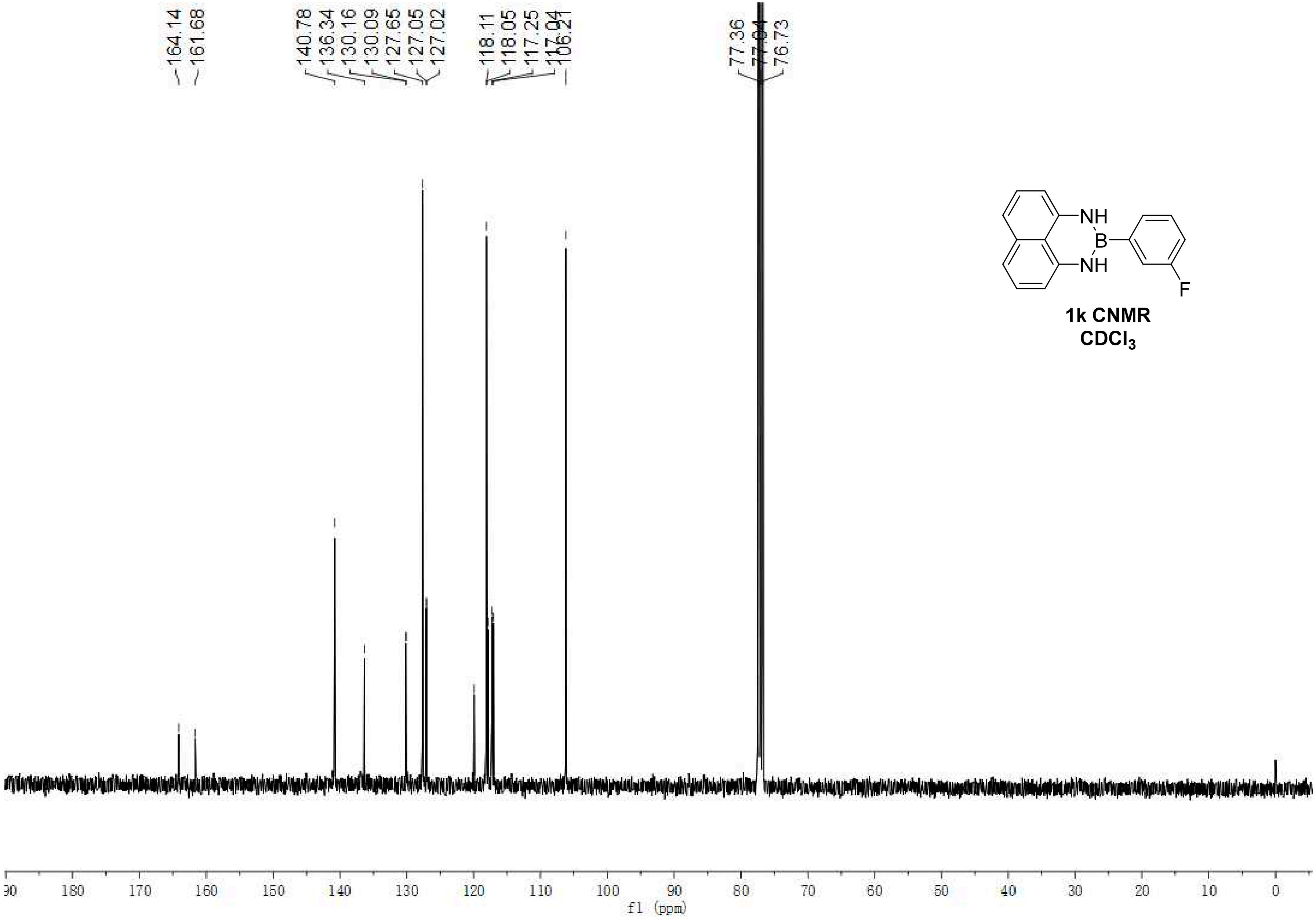
164.14
161.68
140.78
136.34
130.16
130.09
127.65
127.05
127.02
118.11
118.05
117.25
117.04
106.21
77.36
77.04
76.73
NH
B
NH
F
1k CNMR
CDCl3
90
180
170
160
150
140
130
120
110
100
90
80
70
60
50
40
30
20
10
0
f1 (ppm)

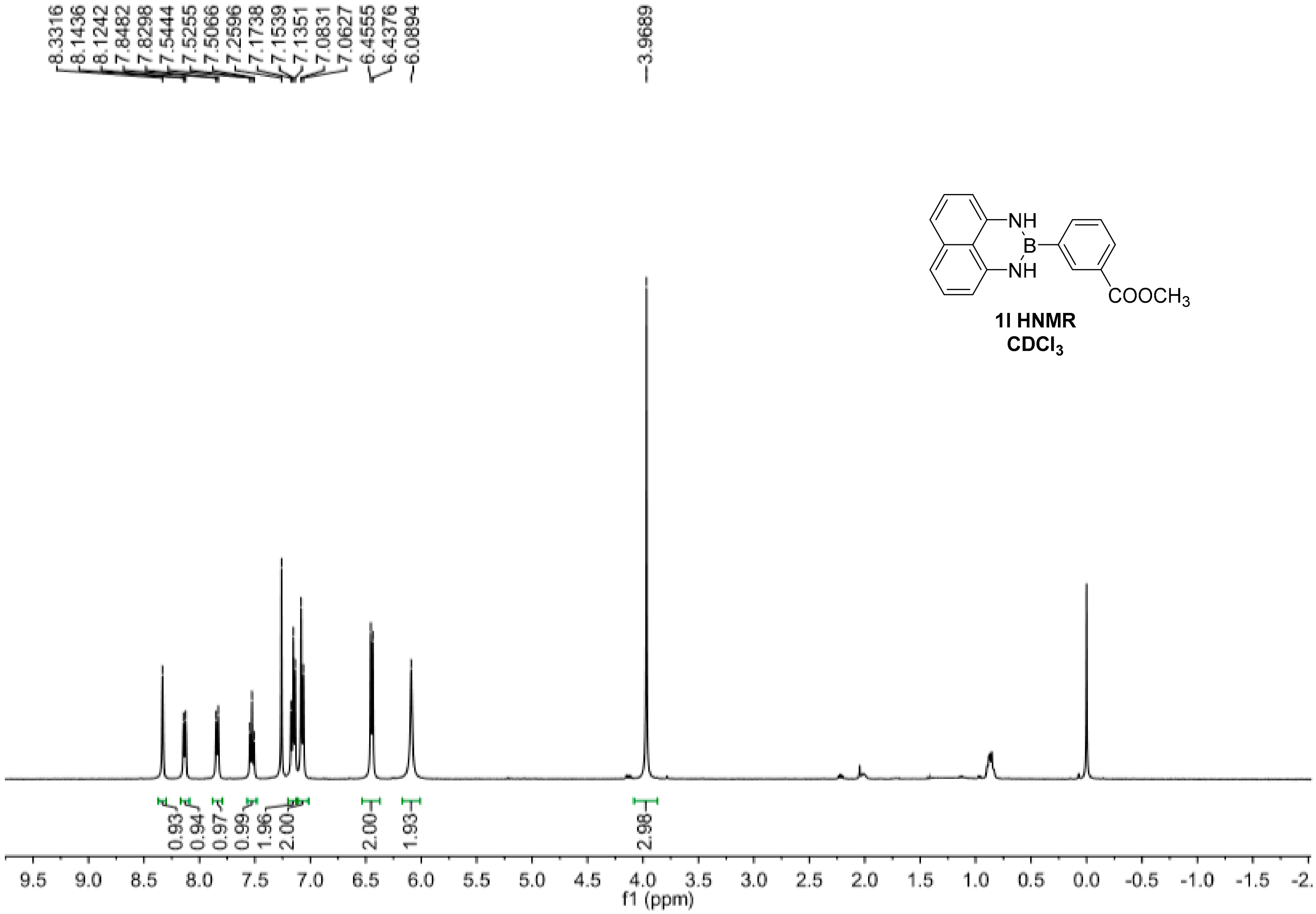
8.3316
8.1436
8.1242
7.8482
7.8298
7.5444
7.5255
7.5066
7.2596
7.1738
7.1539
7.1351
7.0831
7.0627
6.4555
6.4376
6.0894
3.9689
NH
B
NH
COOCH3
1l HNMR
CDCl3
0.93
0.94
0.97
0.99
1.96
2.00
2.00
1.93
2.98
f1 (ppm)

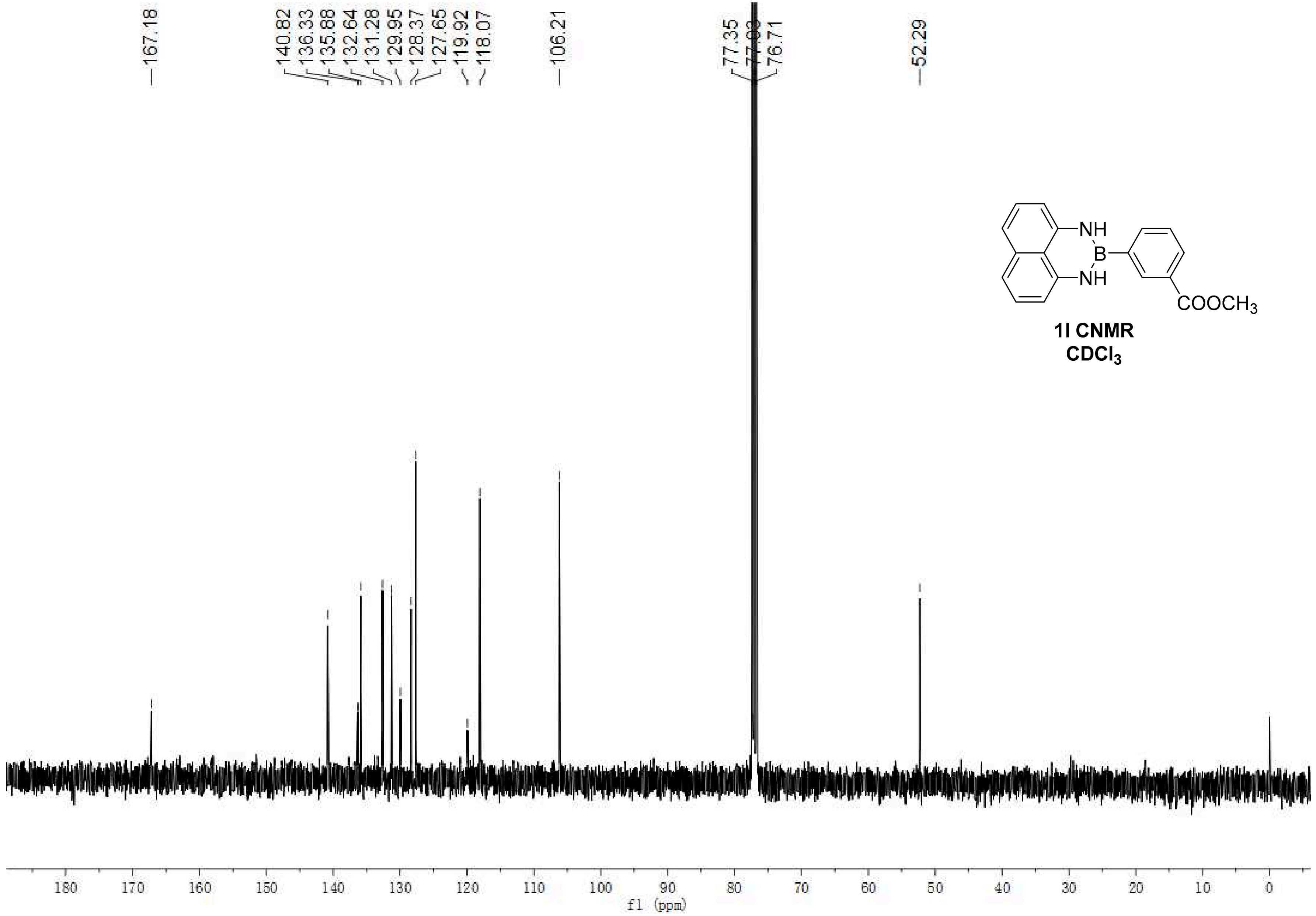
167.18
140.82
136.33
135.88
132.64
131.28
129.95
128.37
127.65
119.92
118.07
106.21
77.35
76.71
52.29
NH
B
NH
COOCH3
1l CNMR
CDCl3
180
170
160
150
140
130
120
110
100
90
80
70
60
50
40
30
20
10
0
f1 (ppm)

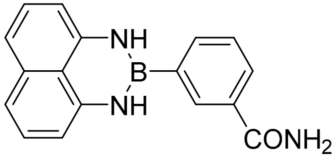

1m HNMR
$CDCl_3$

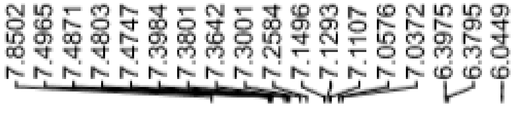

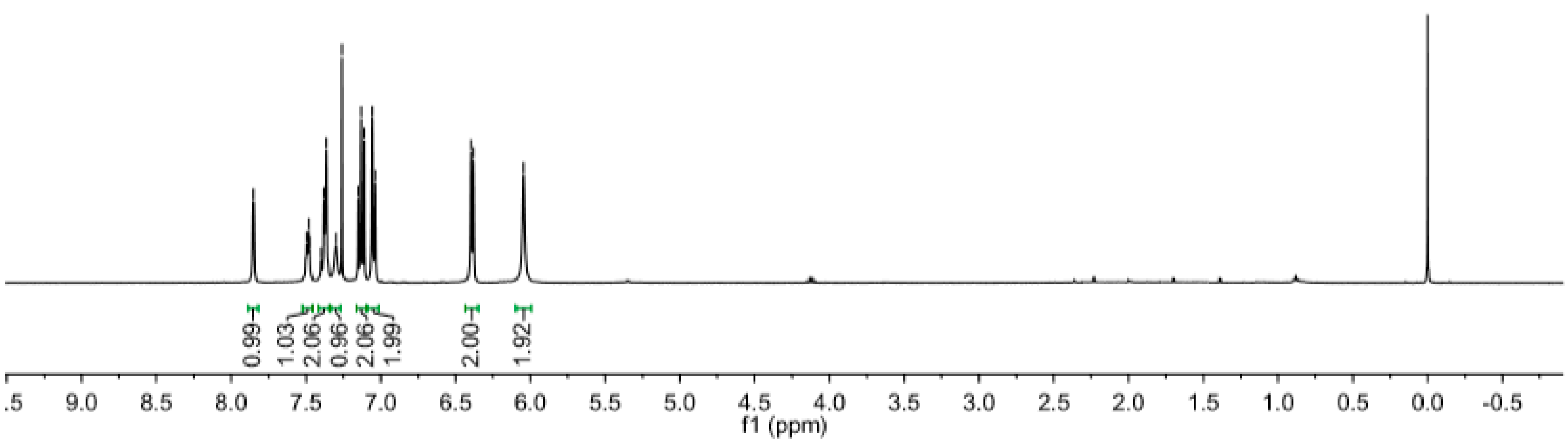

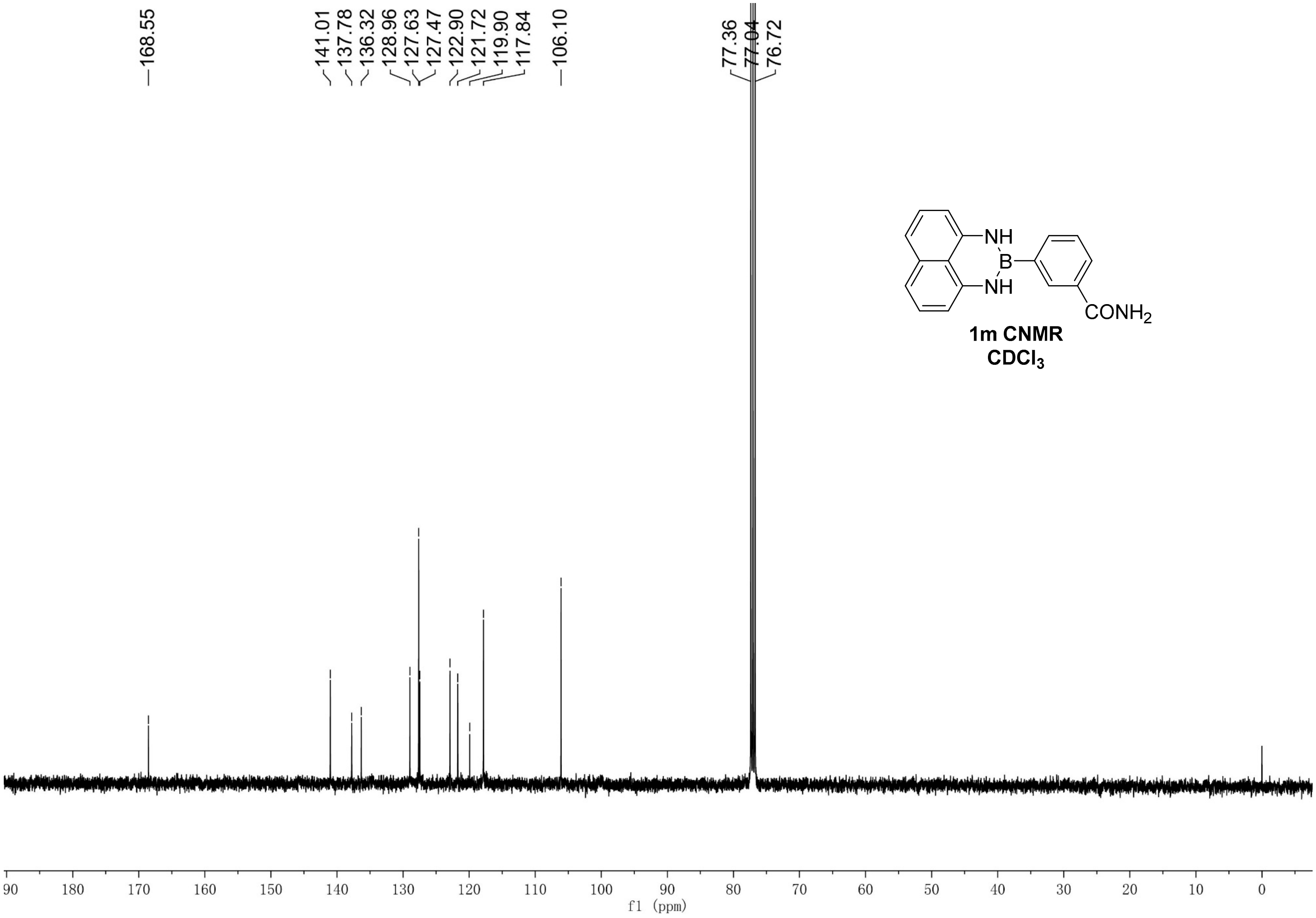
168.55
141.01
137.78
136.32
128.96
127.63
127.47
122.90
121.72
119.90
117.84
106.10
77.36
77.04
76.72
NH
B
NH
CONH2
1m CNMR
CDCl3
90
180
170
160
150
140
130
120
110
100
90
80
70
60
50
40
30
20
10
0
f1 (ppm)

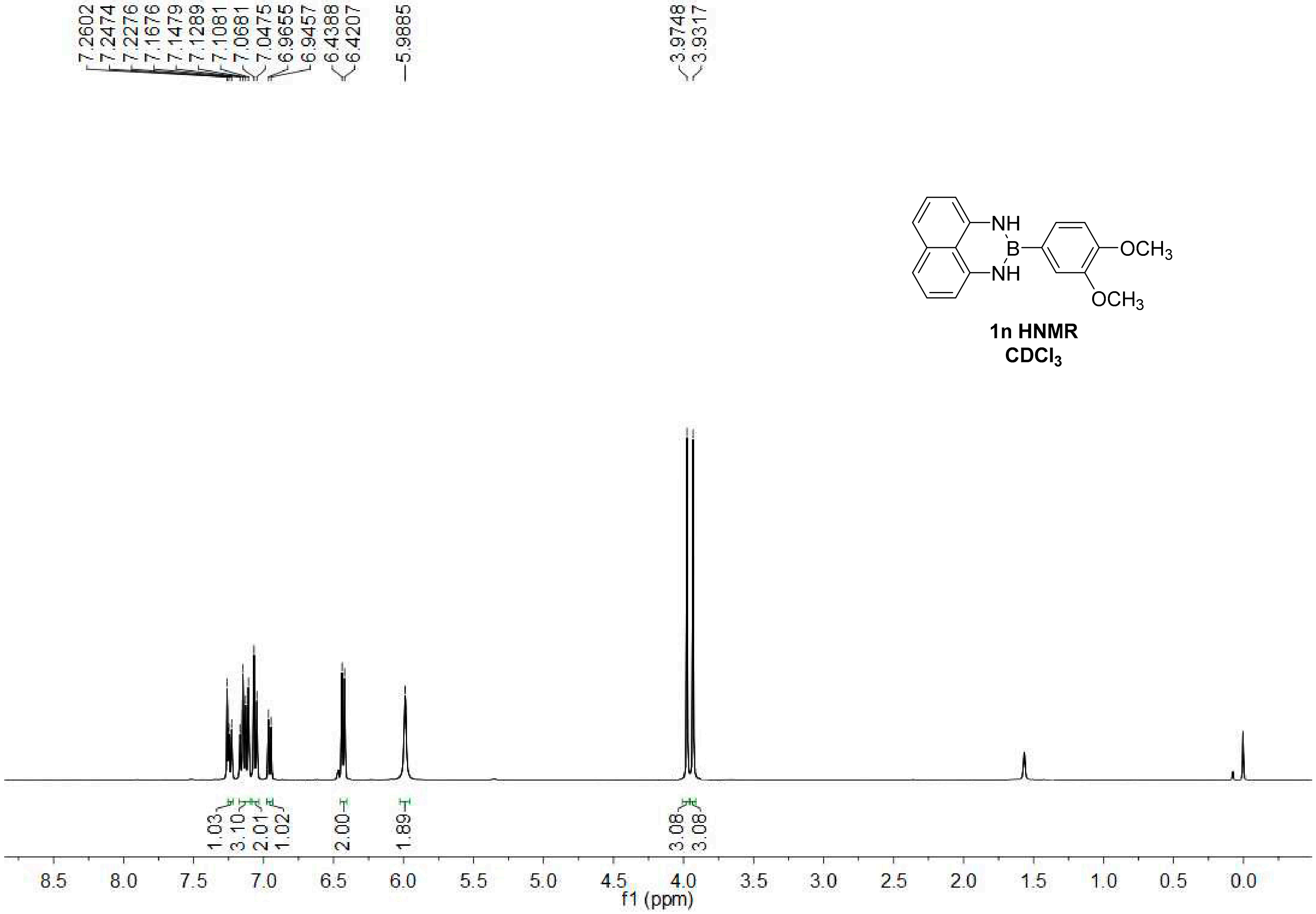
7.2602
7.2474
7.2276
7.1676
7.1479
7.1289
7.1081
7.0681
7.0475
6.9655
6.9457
6.4388
6.4207
5.9885
3.9748
3.9317
NH
B
NH
OCH3
OCH3
1n HNMR
CDCl3
1.03
3.10
2.01
1.02
2.00
1.89
3.08
3.08
8.5
8.0
7.5
7.0
6.5
6.0
5.5
5.0
4.5
4.0
3.5
3.0
2.5
2.0
1.5
1.0
0.5
0.0
f1 (ppm)

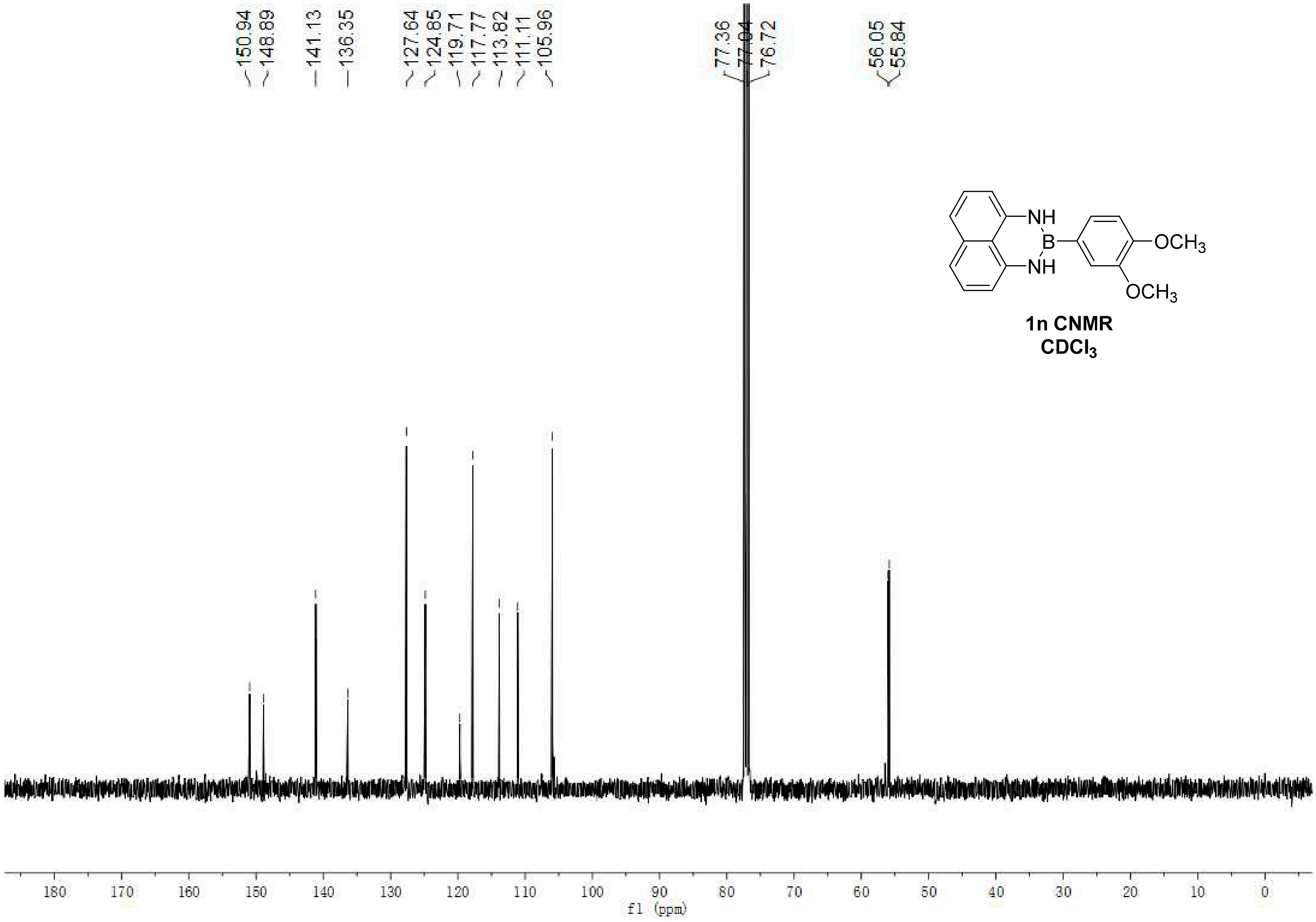
150.94
148.89
141.13
136.35
127.64
124.85
119.71
117.77
113.82
111.11
105.96
77.36
77.04
76.72
56.05
55.84
NH
B
NH
OCH3
OCH3
1n CNMR
CDCl3
180
170
160
150
140
130
120
110
100
90
80
70
60
50
40
30
20
10
0
f1 (ppm)

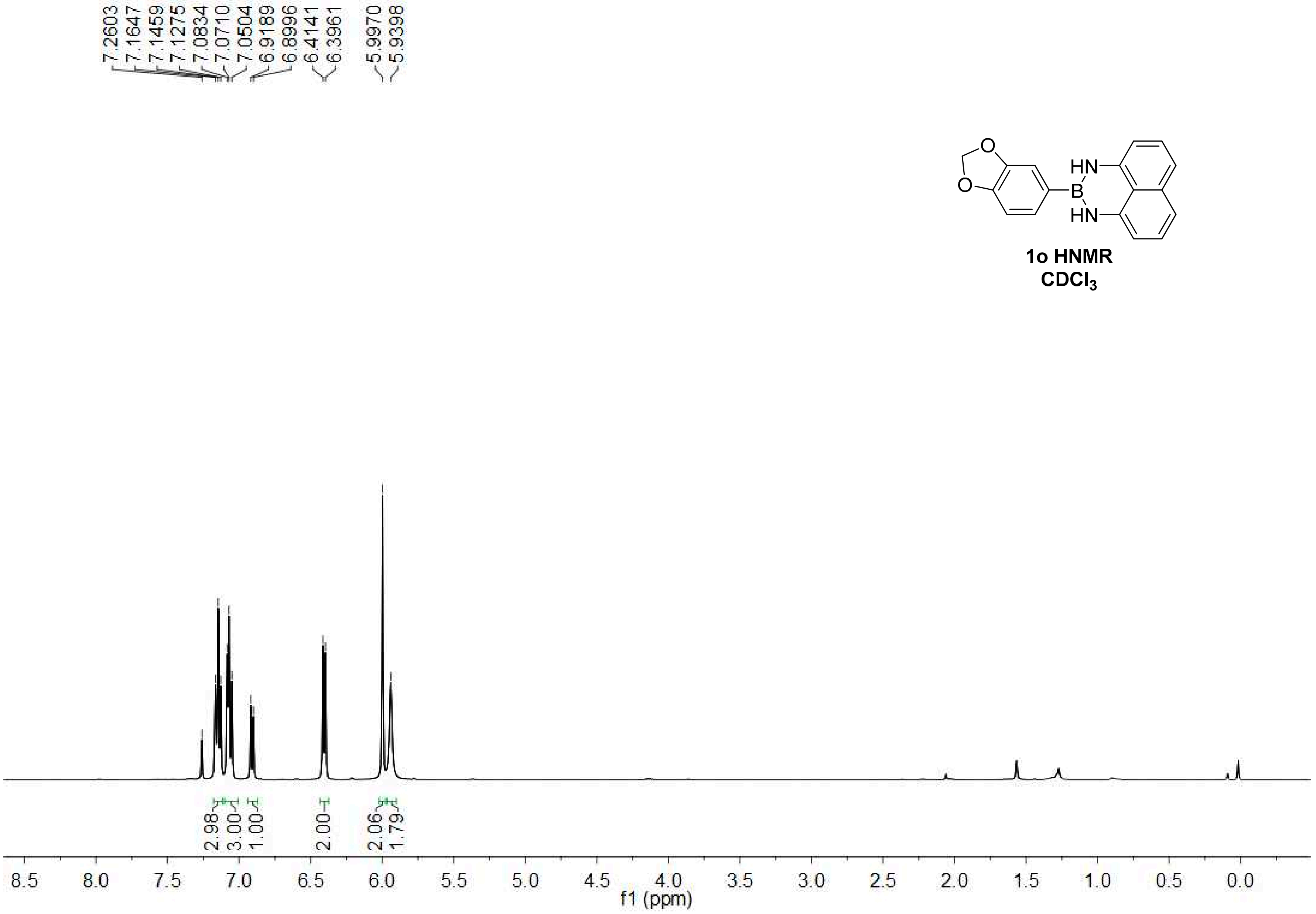

1o HNMR
$CDCl_3$

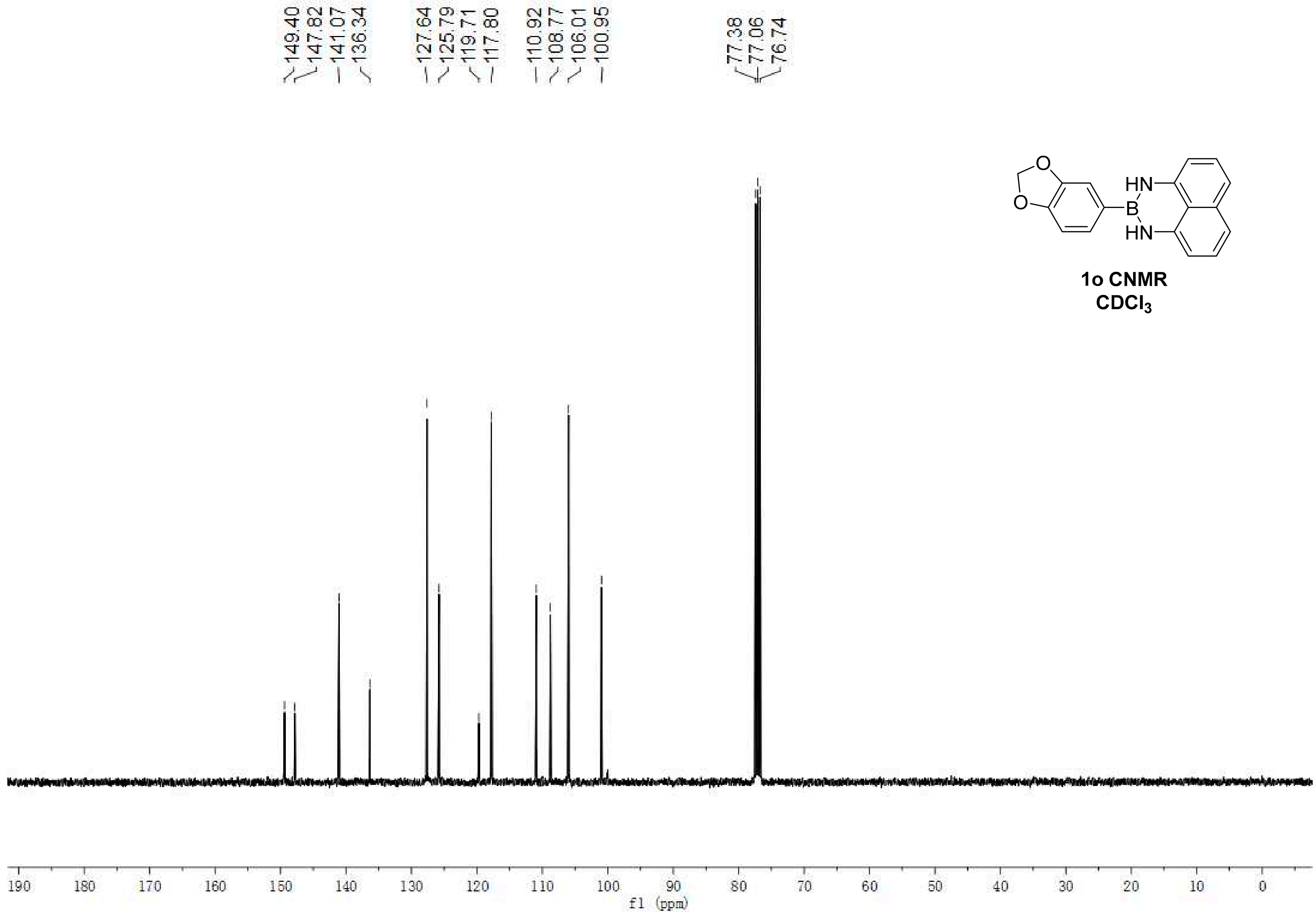

149.40
147.82
141.07
136.34
127.64
125.79
119.71
117.80
110.92
108.77
106.01
100.95
77.38
77.06
76.74
O
O
HN
B
HN
1o CNMR
CDCl3
190
180
170
160
150
140
130
120
110
100
90
80
70
60
50
40
30
20
10
0
f1 (ppm)

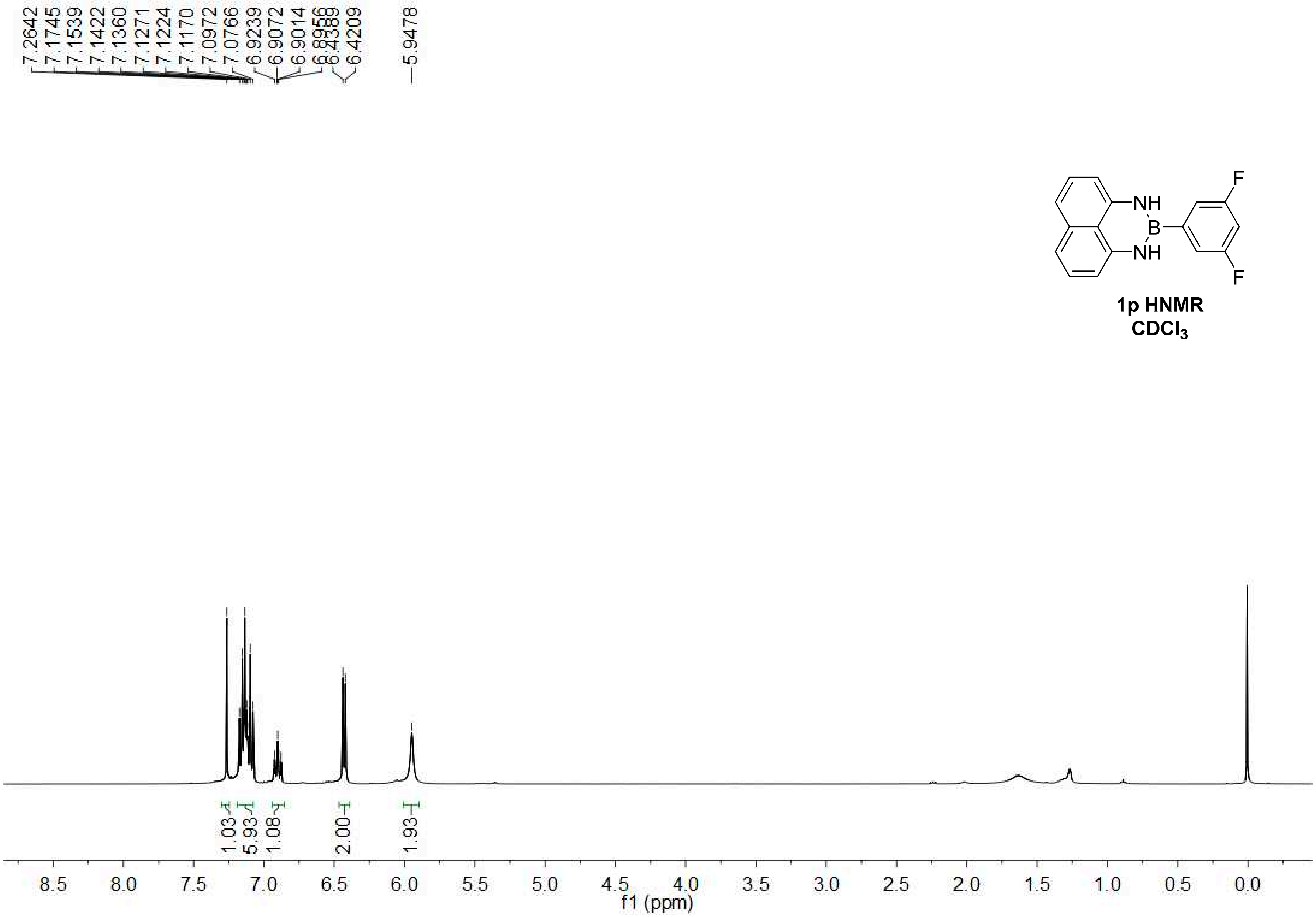

1p HNMR
$CDCl_3$

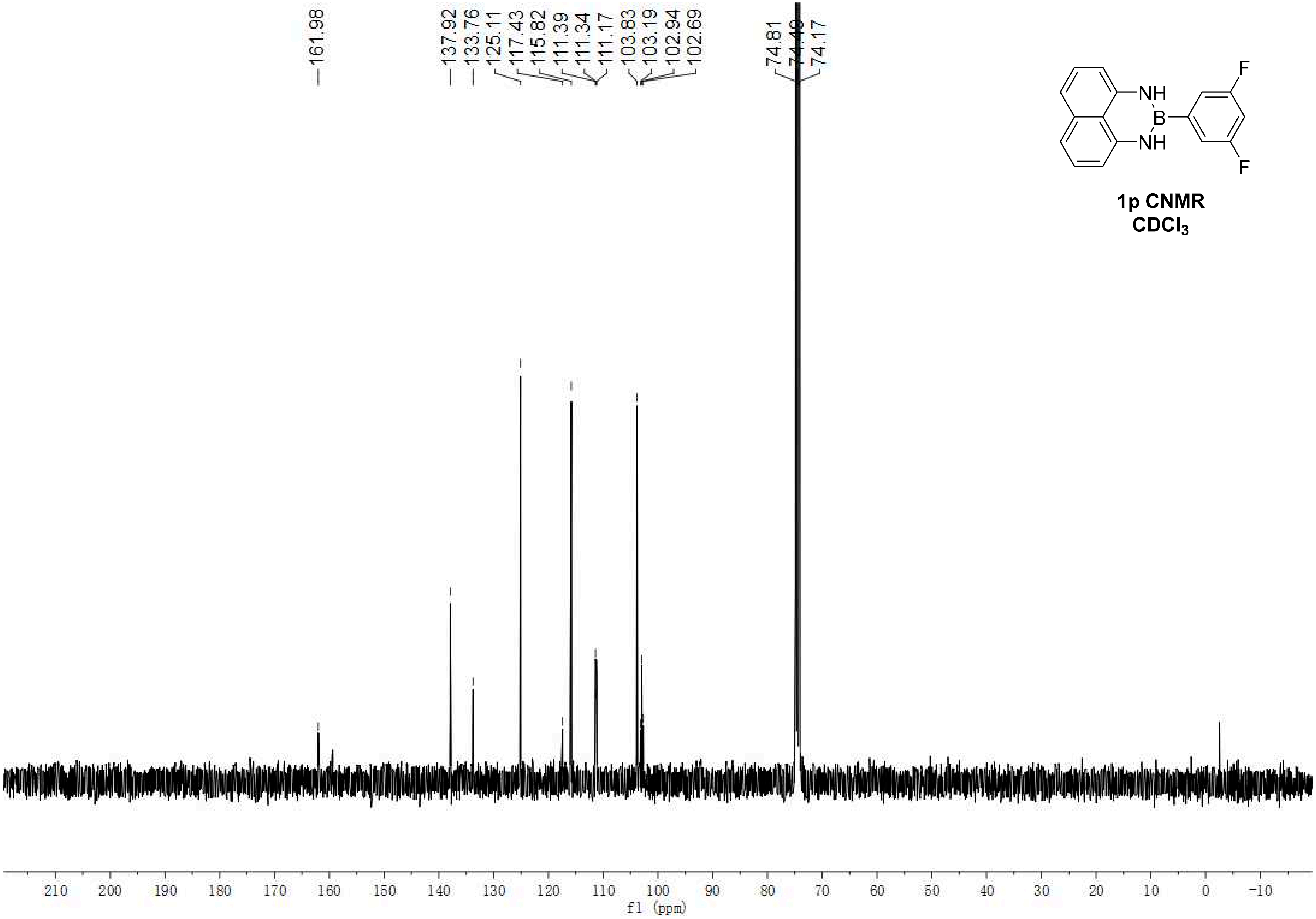

1p CNMR
$CDCl_3$

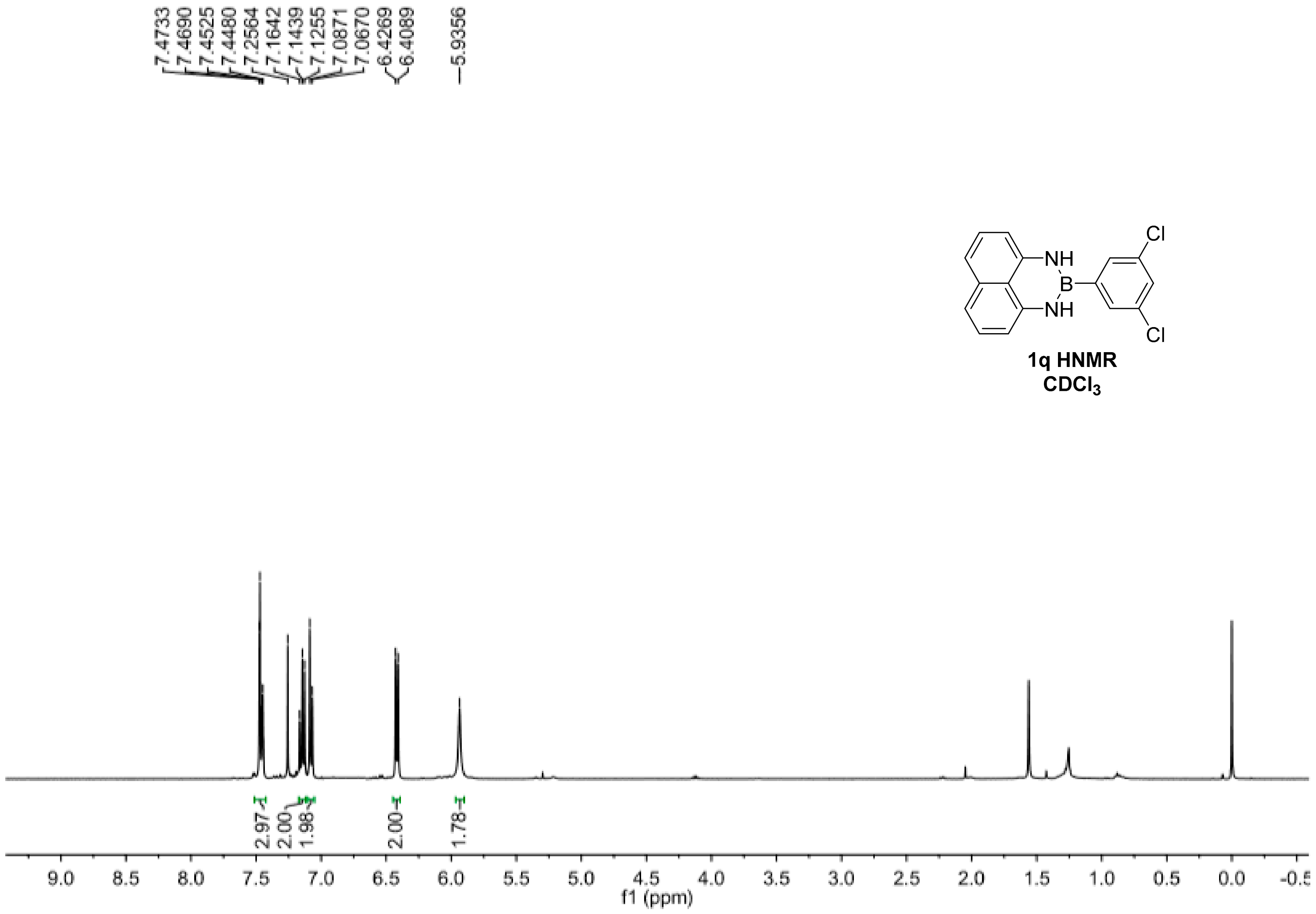
7.4733
7.4690
7.4525
7.4480
7.2564
7.1642
7.1439
7.1255
7.0871
7.0670
6.4269
6.4089
5.9356
NH
B
NH
Cl
Cl
1q HNMR
$CDCl_3$
2.97
2.00
1.98
2.00
1.78
9.0 8.5 8.0 7.5 7.0 6.5 6.0 5.5 5.0 4.5 4.0 3.5 3.0 2.5 2.0 1.5 1.0 0.5 0.0 -0.5
f1 (ppm)

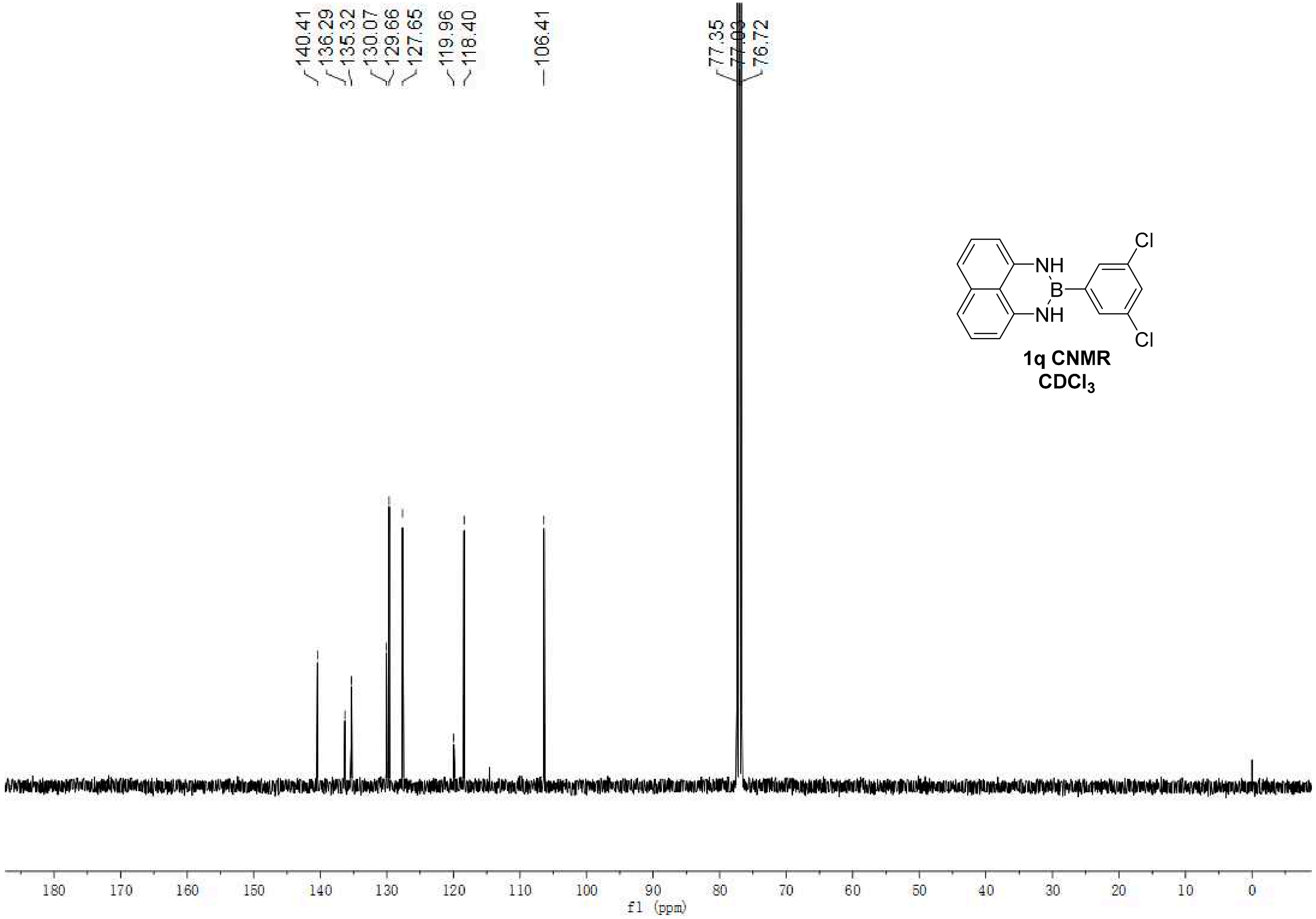

140.41
136.29
135.32
130.07
129.66
127.65
119.96
118.40
106.41
77.35
77.03
76.72
NH
B
NH
Cl
Cl
1q CNMR
CDCl3
180
170
160
150
140
130
120
110
100
90
80
70
60
50
40
30
20
10
0
f1 (ppm)

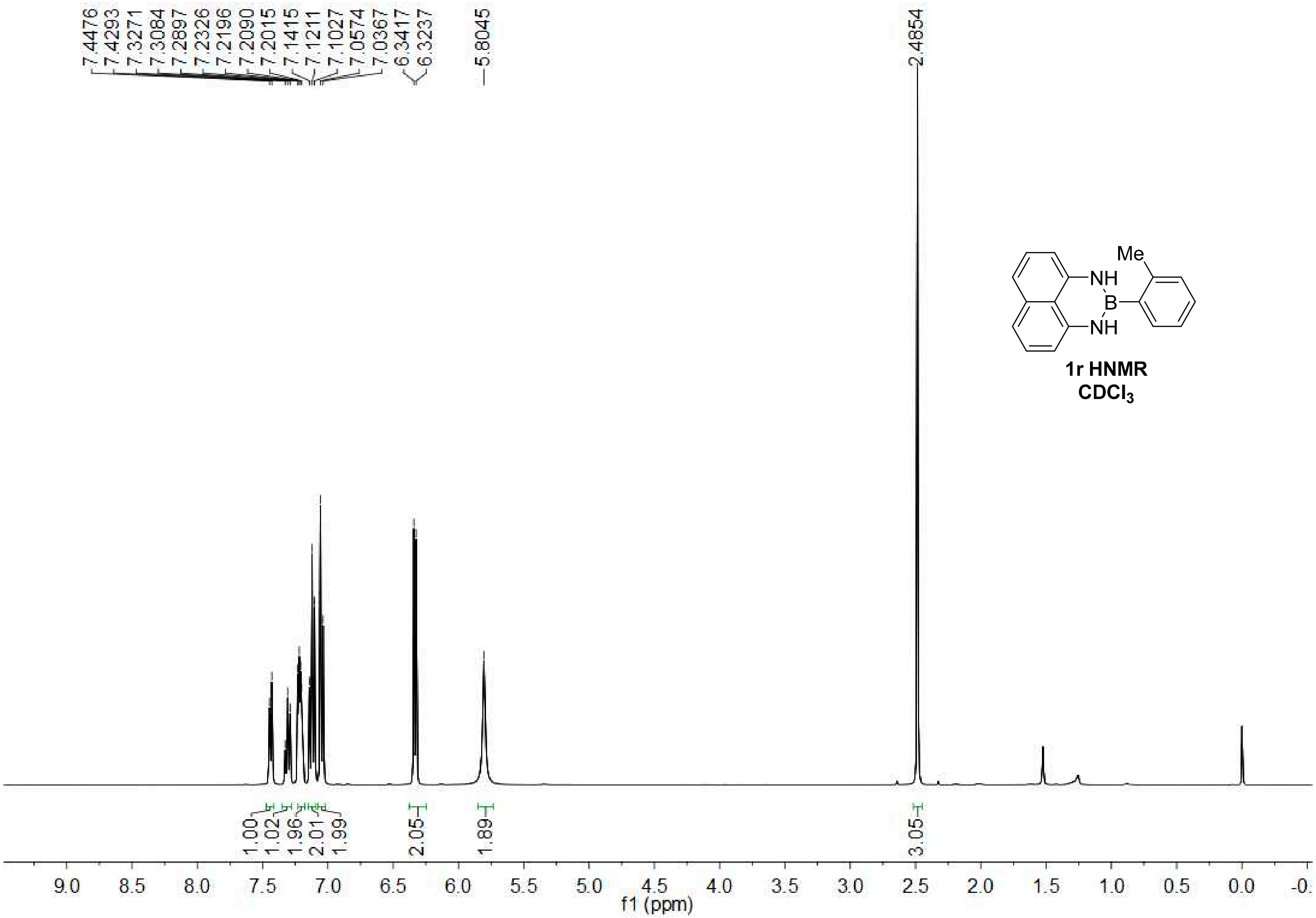
7.4476
7.4293
7.3271
7.3084
7.2897
7.2326
7.2196
7.2090
7.2015
7.1415
7.1211
7.1027
7.0574
7.0367
6.3417
6.3237
5.8045
2.4854
Me
NH
B
NH
1r HNMR
CDCl3
1.00
1.02
1.96
2.01
1.99
2.05
1.89
3.05
9.0
8.5
8.0
7.5
7.0
6.5
6.0
5.5
5.0
4.5
4.0
3.5
3.0
2.5
2.0
1.5
1.0
0.5
0.0
f1 (ppm)

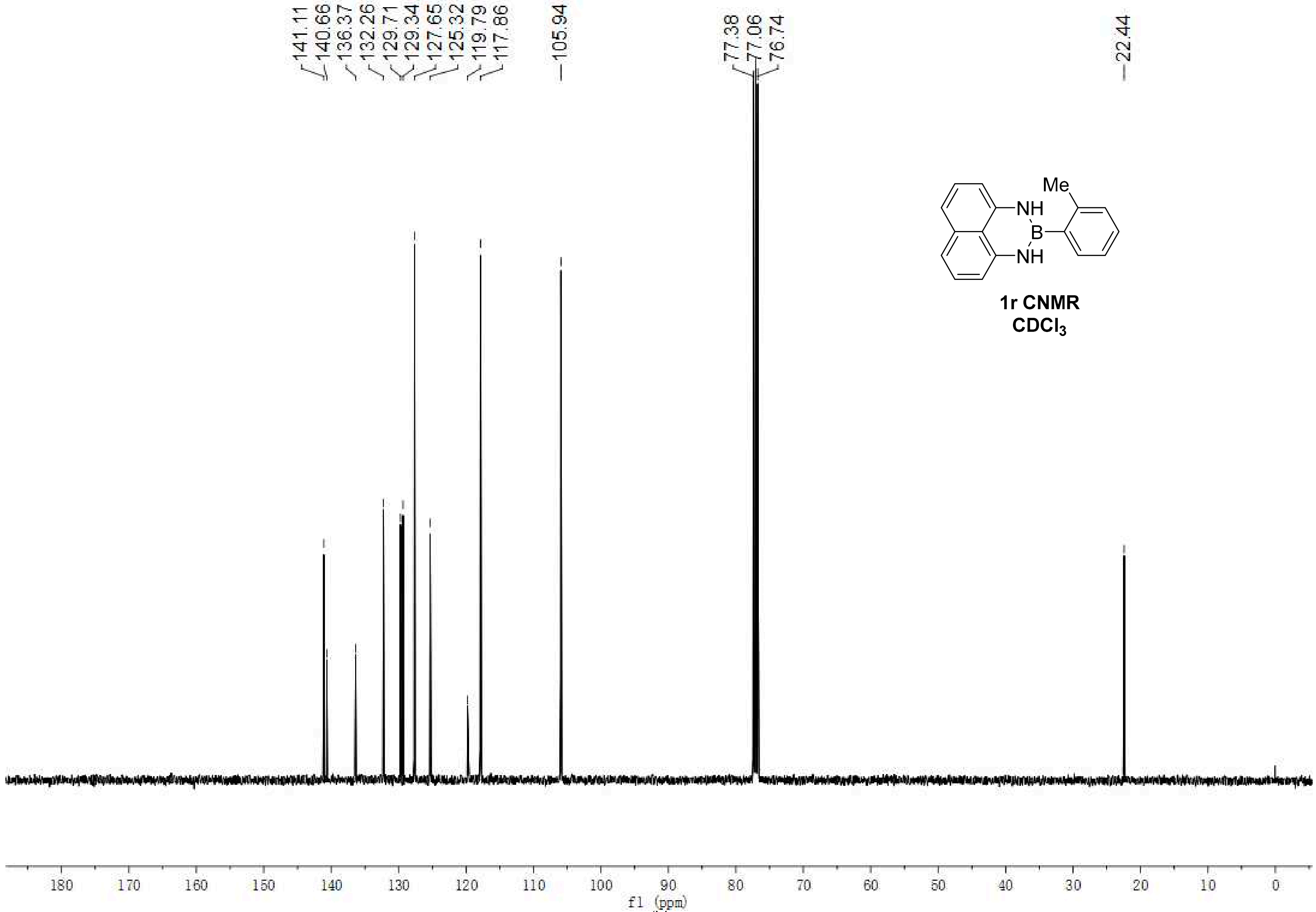
141.11
140.66
136.37
132.26
129.71
129.34
127.65
125.32
119.79
117.86
105.94
77.38
77.06
76.74
22.44
Me
NH
B
NH
1r CNMR
CDCl3
180 170 160 150 140 130 120 110 100 90 80 70 60 50 40 30 20 10 0
f1 (ppm)

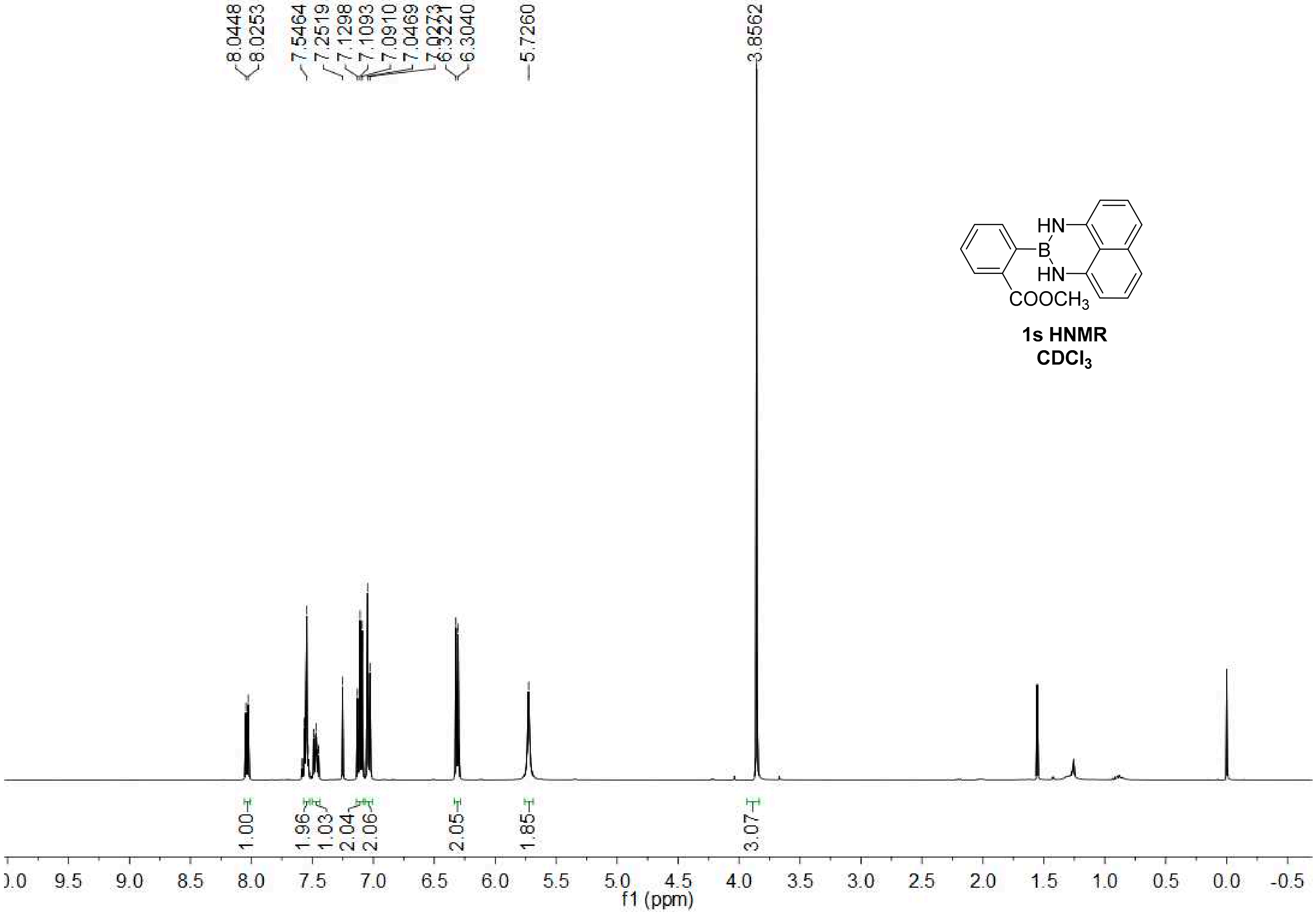
8.0448
8.0253
7.5464
7.2519
7.1298
7.1093
7.0910
7.0469
7.0273
6.3221
6.3040
5.7260
3.8562
HN
B
HN
COOCH3
1s HNMR
CDCl3
1.00
1.96
1.03
2.04
2.06
2.05
1.85
3.07
f1 (ppm)

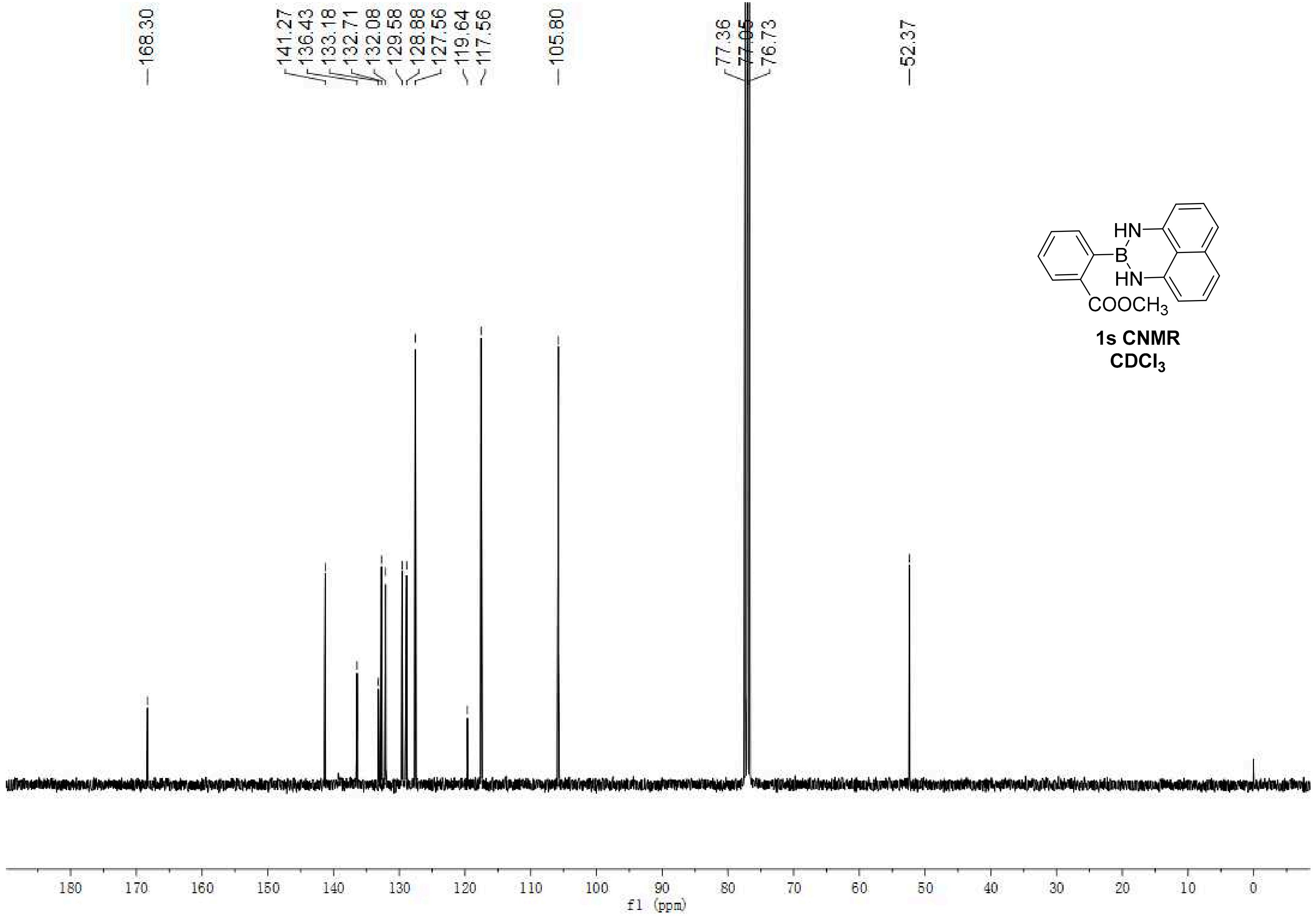
168.30
141.27
136.43
133.18
132.71
132.08
129.58
128.88
127.56
119.64
117.56
105.80
77.36
77.05
76.73
52.37
HN
B
HN
COOCH3
1s CNMR
CDCl3
180 170 160 150 140 130 120 110 100 90 80 70 60 50 40 30 20 10 0
f1 (ppm)

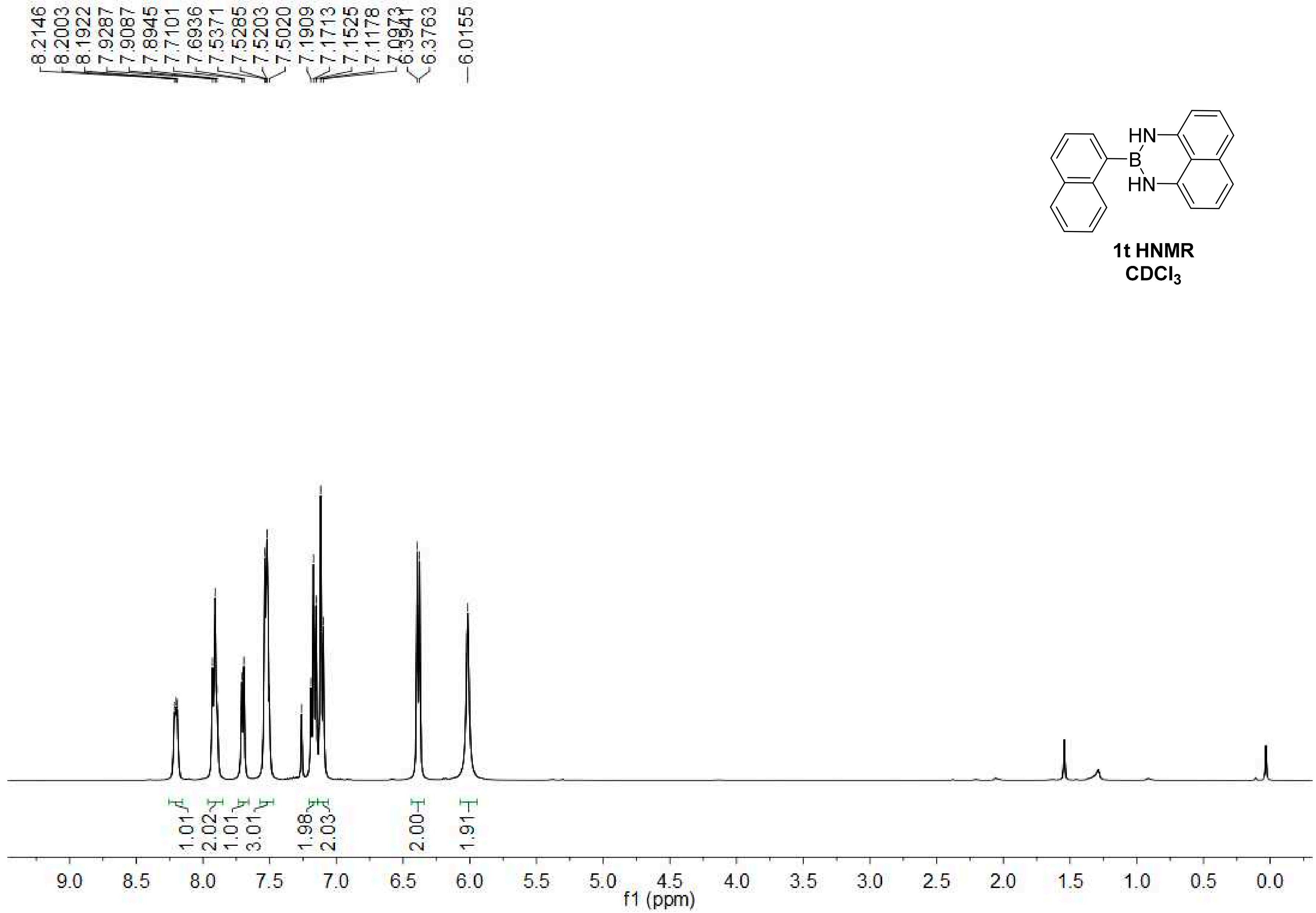

1t HNMR
$CDCl_3$

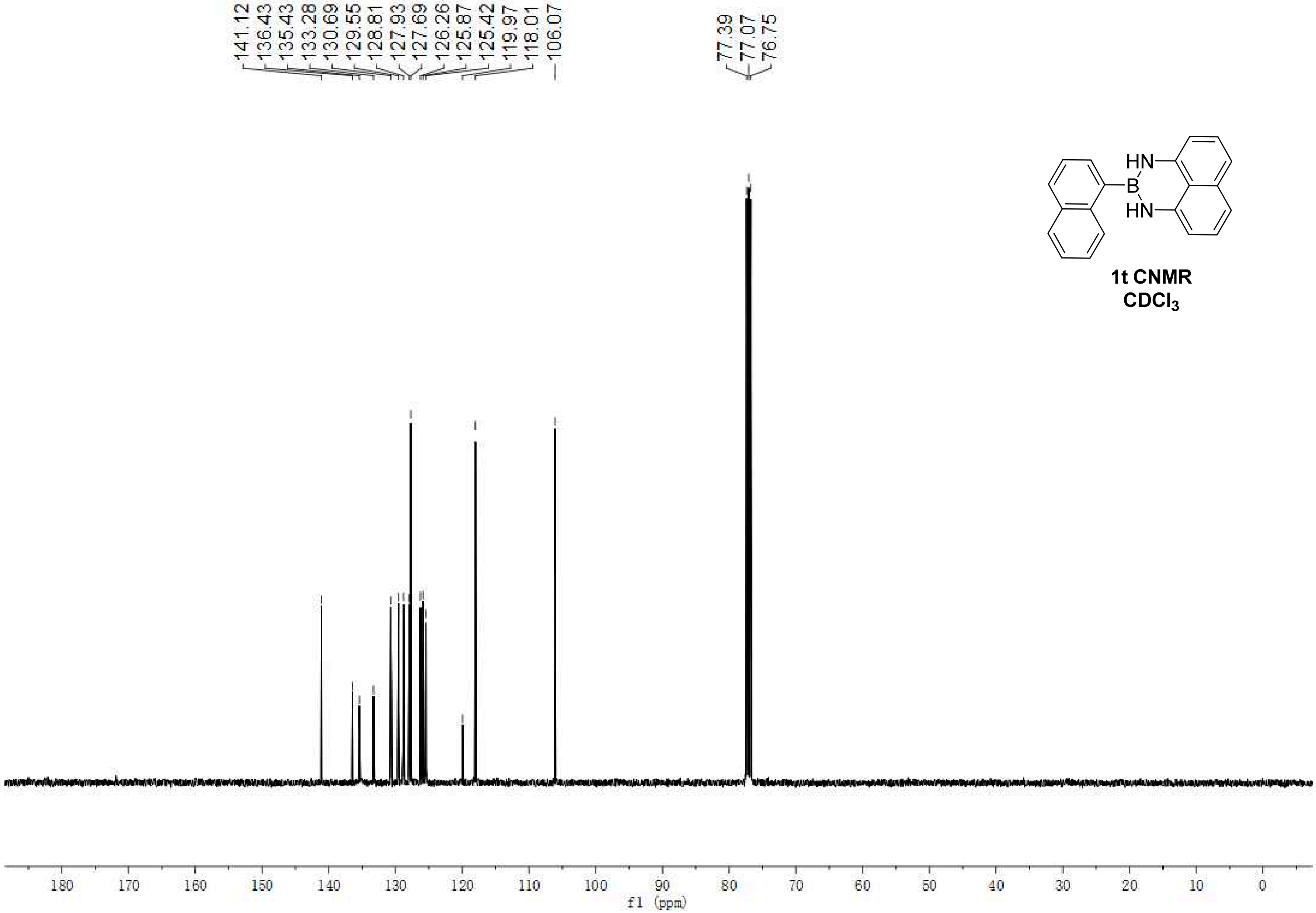

1t CNMR
$CDCl_3$

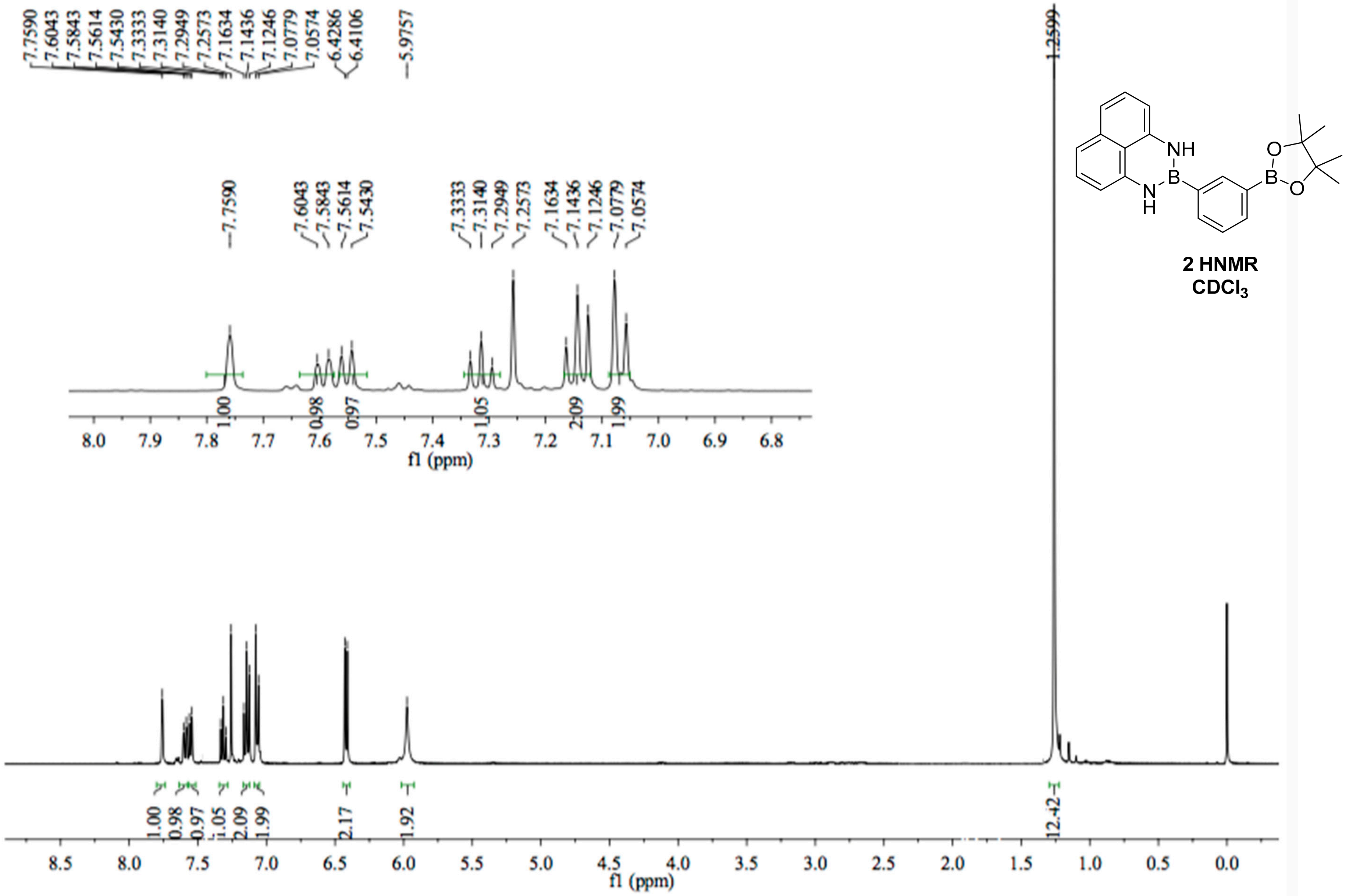
7.7590
7.6043
7.5843
7.5614
7.5430
7.3333
7.3140
7.2949
7.2573
7.1634
7.1436
7.1246
7.0779
7.0574
6.4286
6.4106
5.9757
1.2599
1.00
0.98
0.97
1.05
2.09
1.99
2.17
1.92
12.42
f1 (ppm)
NH
B
N
H
O
B
O
2 HNMR
CDCl3

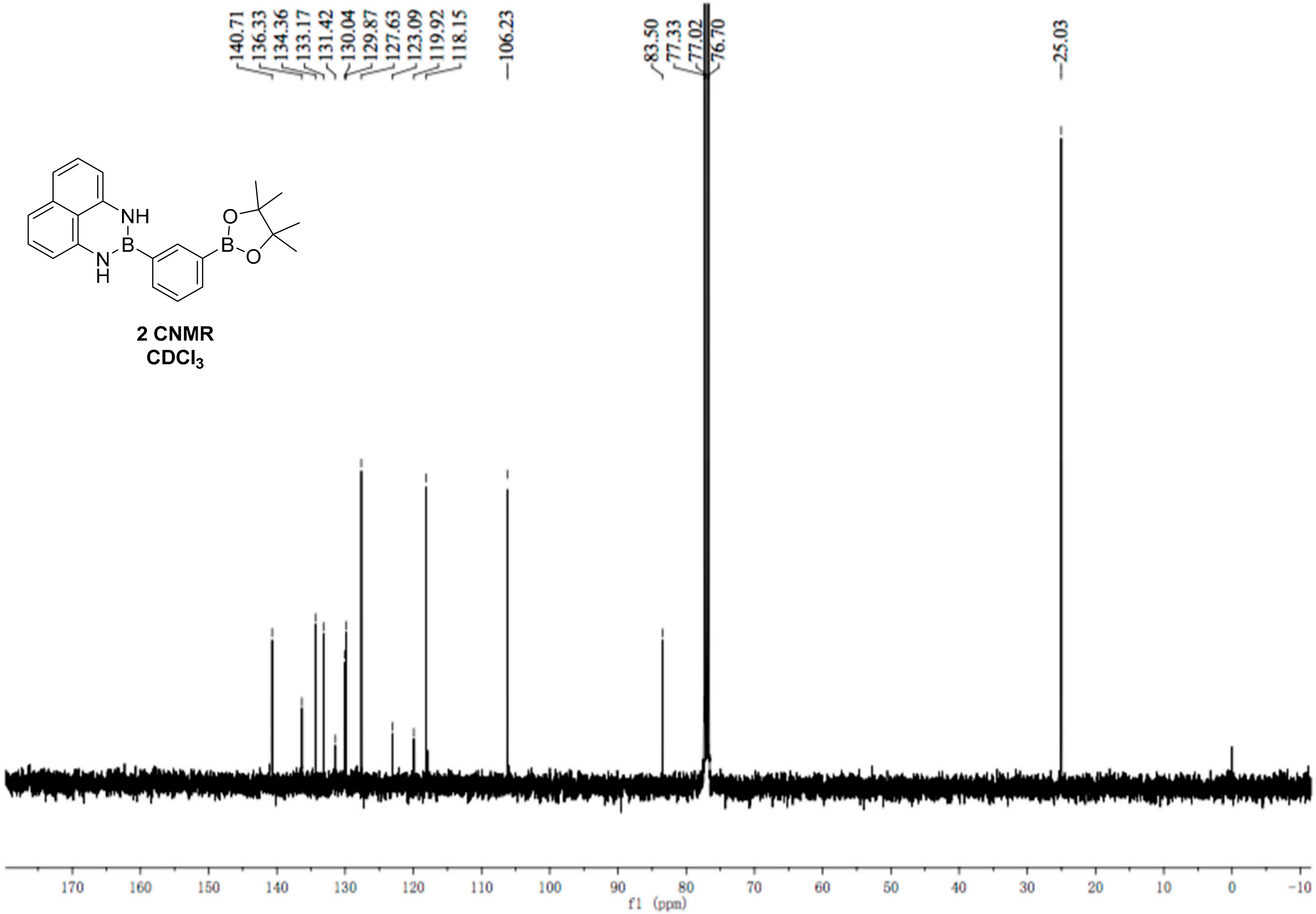
140.71
136.33
134.36
133.17
131.42
130.04
129.87
127.63
123.09
119.92
118.15
106.23
83.50
77.33
77.02
76.70
25.03
170 160 150 140 130 120 110 100 90 80 70 60 50 40 30 20 10 0 -10
f1 (ppm)

2 CNMR
$CDCl_3$

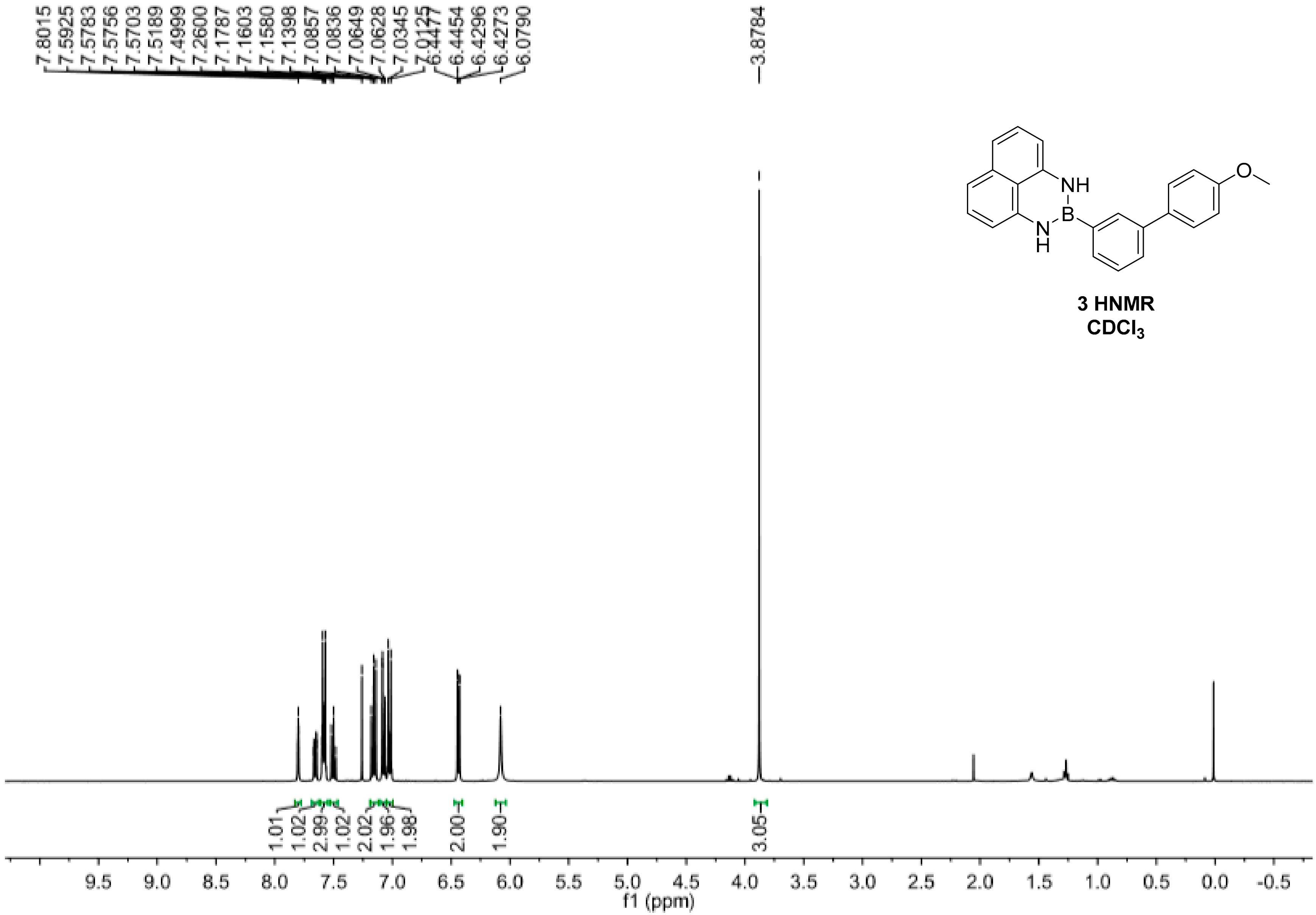
7.8015
7.5925
7.5783
7.5756
7.5703
7.5189
7.4999
7.2600
7.1787
7.1603
7.1580
7.1398
7.0857
7.0836
7.0649
7.0628
7.0345
7.0125
6.4477
6.4454
6.4296
6.4273
6.0790
3.8784
NH
B
N
H
O
3 HNMR
CDCl3
1.01
1.02
2.99
1.02
2.02
1.96
1.98
2.00
1.90
3.05
9.5 9.0 8.5 8.0 7.5 7.0 6.5 6.0 5.5 5.0 4.5 4.0 3.5 3.0 2.5 2.0 1.5 1.0 0.5 0.0 -0.5
f1 (ppm)

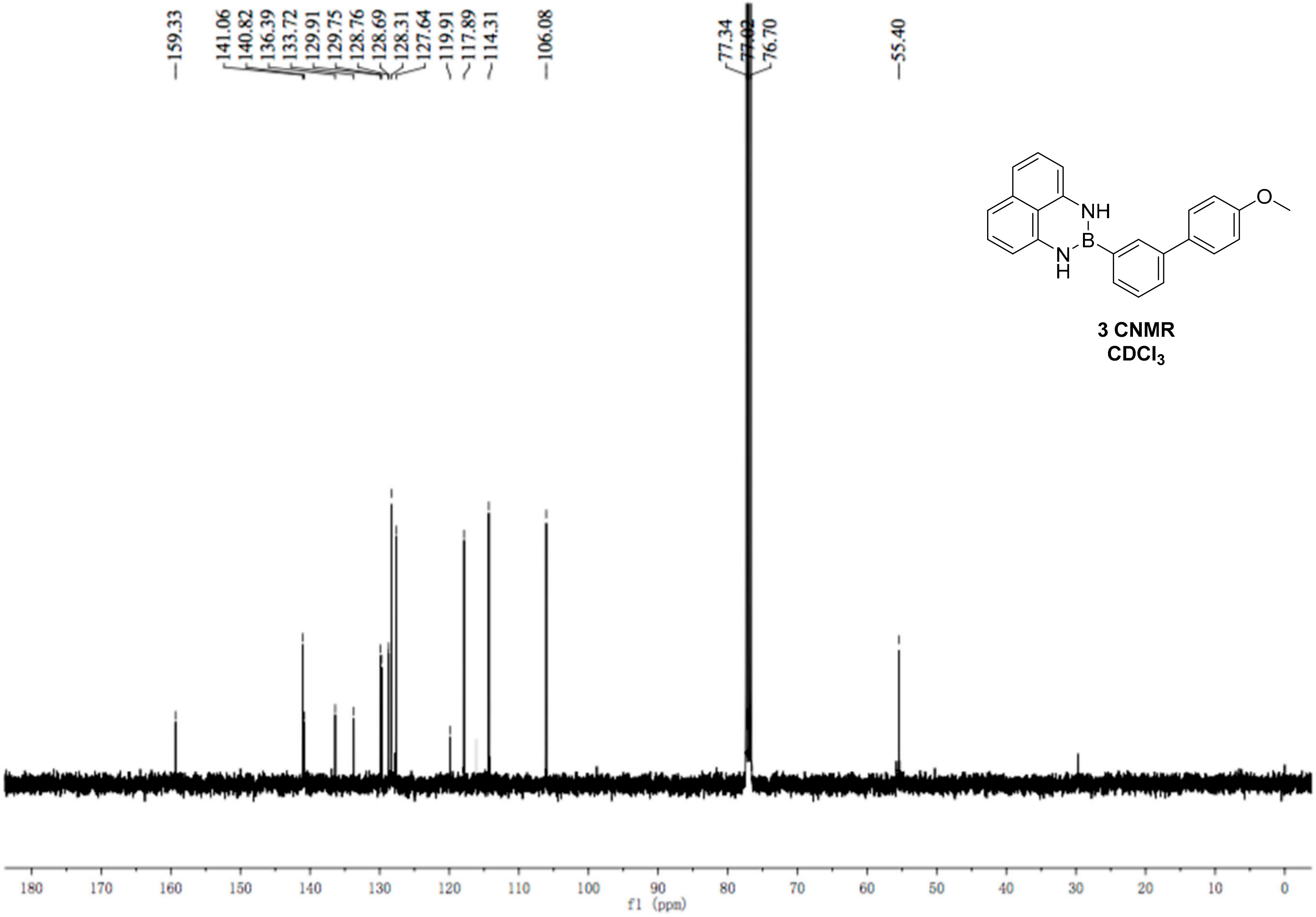
159.33
141.06
140.82
136.39
133.72
129.91
129.75
128.76
128.69
128.31
127.64
119.91
117.89
114.31
106.08
77.34
77.02
76.70
55.40
NH
B
N
H
O
3 CNMR
$CDCl_3$
180 170 160 150 140 130 120 110 100 90 80 70 60 50 40 30 20 10 0
f1 (ppm)

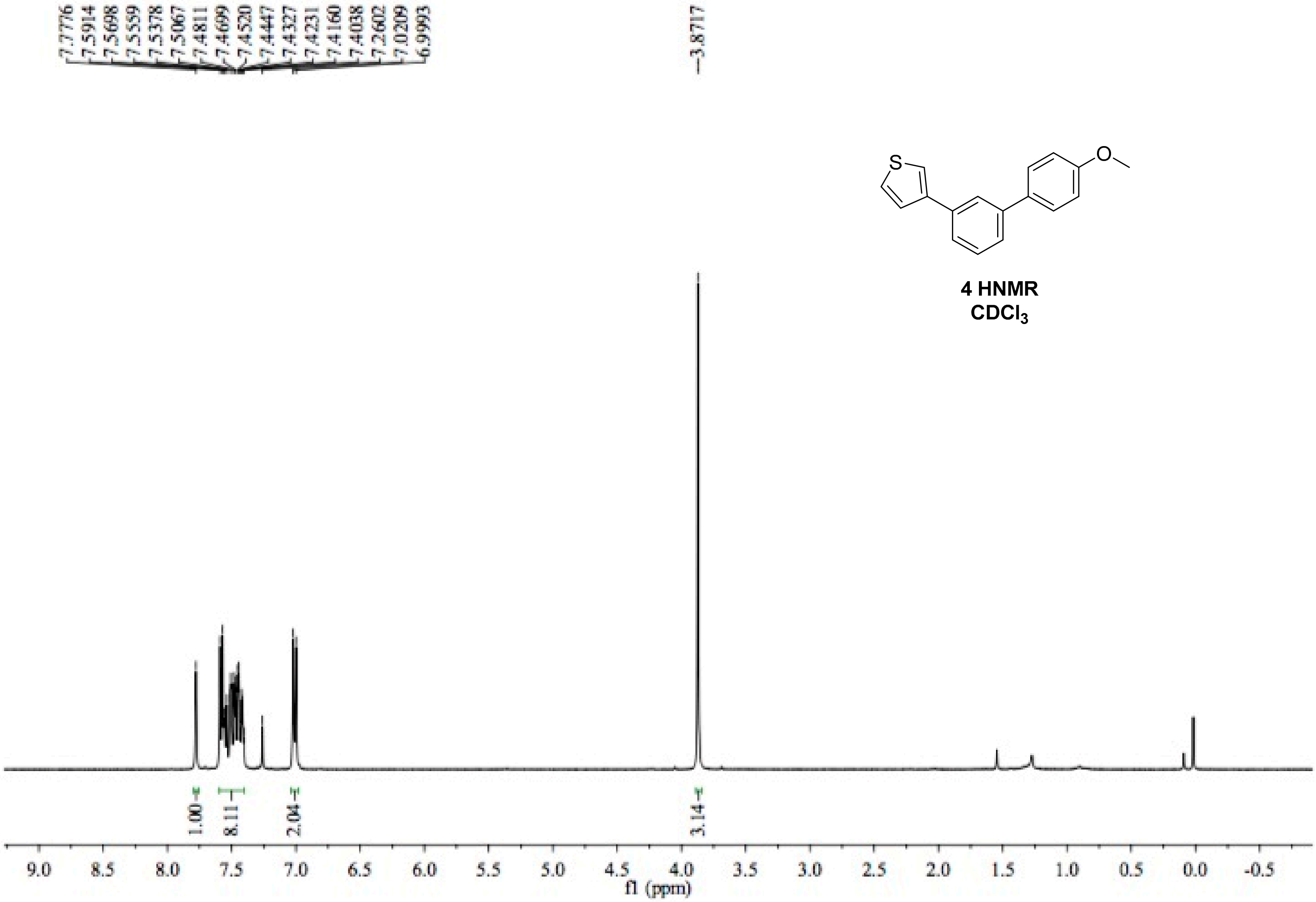
7.7776
7.5914
7.5698
7.5559
7.5378
7.5067
7.4811
7.4699
7.4520
7.4447
7.4327
7.4231
7.4160
7.4038
7.2602
7.0209
6.9993
3.8717
1.00
8.11
2.04
3.14
9.0 8.5 8.0 7.5 7.0 6.5 6.0 5.5 5.0 4.5 4.0 3.5 3.0 2.5 2.0 1.5 1.0 0.5 0.0 -0.5
f1 (ppm)
S
O

4 HNMR
$CDCl_3$

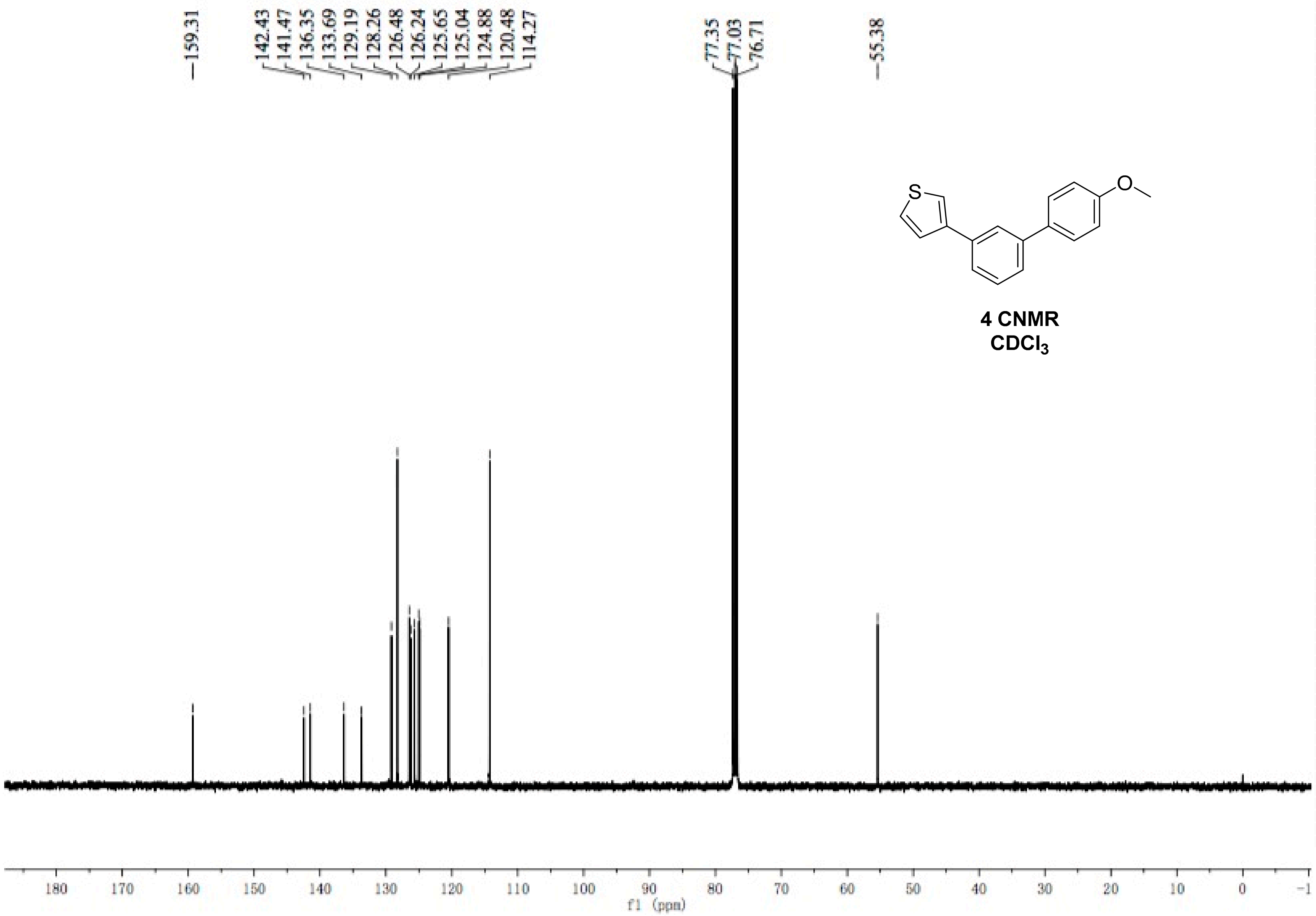
159.31
142.43
141.47
136.35
133.69
129.19
128.26
126.48
126.24
125.65
125.04
124.88
120.48
114.27
77.35
77.03
76.71
55.38
S
O
4 CNMR
$CDCl_3$
180 170 160 150 140 130 120 110 100 90 80 70 60 50 40 30 20 10 0 −1
f1 (ppm)
